# Comparison of DNA targeting CRISPR editors in human cells

**DOI:** 10.1186/s13578-023-00958-z

**Published:** 2023-01-16

**Authors:** Hongxin Huang, Weiqi Lv, Jinhe Li, Guanjie Huang, Zhihong Tan, Yongfei Hu, Shufeng Ma, Xin Zhang, Linxuan Huang, Ying Lin

**Affiliations:** 1 Cancer Research Institute, School of Basic Medical Sciences, Southern Medical University, Guangzhou, 510515 China; 2 Dermatology Hospital, Southern Medical University, Guangzhou, 510091 China; 3 Affiliated Cancer Hospital & Institute of Guangzhou Medical University, Guangzhou, 510095 China; 4 Department of Anesthesiology, The Third Affiliated Hospital of Southern Medical University, Guangzhou, 510630 China; 5 Department of Nephrology, Shenzhen Hospital, Southern Medical University, Shenzhen, 518110 China; 6 Affiliated Dongguan Hospital, Southern Medical University, (Dongguan People’s Hospital), Dongguan, 523058 China; 7 Experimental Education/Administration Center, School of Basic Medical Science, Southern Medical University, Guangzhou, 510515 China

**Keywords:** Cas12f1 nuclease, CRISPR-Cas system, DNA targeting, Specificity, Comparison

## Abstract

**Background:**

Profiling and comparing the performance of current widely used DNA targeting CRISPR systems provide the basic information for the gene-editing toolkit and can be a useful resource for this field. In the current study, we made a parallel comparison between the recently reported miniature Cas12f1 (Un1Cas12f1 and *As*Cas12f1) and the widely used Cas12a and Cas9 nucleases in mammalian cells.

**Results:**

We found that as a CRISPRa activator, Un1Cas12f1 could induce gene expression with a comparable level to that of Cas12a and Cas9, while as a DNA cleavage editor, Cas12f1 exhibited similar properties to Cas12a, like high specificity and dominantly induced deletions over insertions, but with less activity. In contrast, wild-type SpCas9 showed the highest activity, lowest specificity, and induced balanced deletions and insertions. Thus, Cas12f1 is recommended for gene-activation-based applications, Cas12a is for therapy applications, and wild-type Cas9 is for in vitro and animal investigations.

**Conclusion:**

The comparison provided the editing properties of the widely used DNA-targeting CRISPR systems in the gene-editing field.

**Supplementary Information:**

The online version contains supplementary material available at 10.1186/s13578-023-00958-z.

## Background

DNA-targeting CRISPR systems have been developed as powerful tools for basic research and clinical therapy, including programmable DNA editing, gene activation/suppression, live imaging, base editing, and primer editing [1, 2]. However, therapeutic delivery of these systems remains challenging, in part because their sizes exceed the packaging capacity (< 4.7 kb) of adeno-associated virus (AAV), the most widely used viral vector for gene delivery. To overcome this limitation, efforts have been made to explore the miniature Cas-nucleases, such as the *Sa*Cas9 (1053 amino acids) [3], *Cj*Cas9 (984 amino acids) [4], and Cas12j (700–800 amino acids) [5], et al. However, the low editing activity or the relatively large size of these nucleases keep the challenge incompletely solved.

Recently, it has been reported that the type V-F Cas12f (also known as Cas14) nuclease could serve as a hypercompact gene-editing tool in mammalian cells with optimized sgRNA or engineered nuclease mutant [6–10]. Cas12f forms a unique asymmetric dimer structure to bind crRNA and guide target DNA recognition and cleavage [11, 12]. The extremely small size (422 and 529 amino acids for *As*Cas12f1 and Un1Cas12f1, respectively, the two nucleases functional in mammalian cells) makes Cas12f1 an ideal tool for therapeutic editing. However, the editing features of these miniature nucleases in mammalian or human cells have not been well elucidated. For example, is the editing activity or specificity high enough for therapeutic purposes? Where is the cutting site in human cells? Does Cas12f1 induce more insertions or more deletions? Could it be repurposed for transcription activation?

In the current study, we analyzed the editing features of Cas12f1 and made a comparison between Cas12f1 and the other two widely used nucleases, Cas9 and Cas12a. Our data profiled the properties of current widely used DNA targeting CRISPR systems, which can be a guideline and a useful resource for the gene-editing field.

## Results and discussion

### The performance of the engineered CRISPR-Un1Cas12f1 systems

**Fig. 1:**
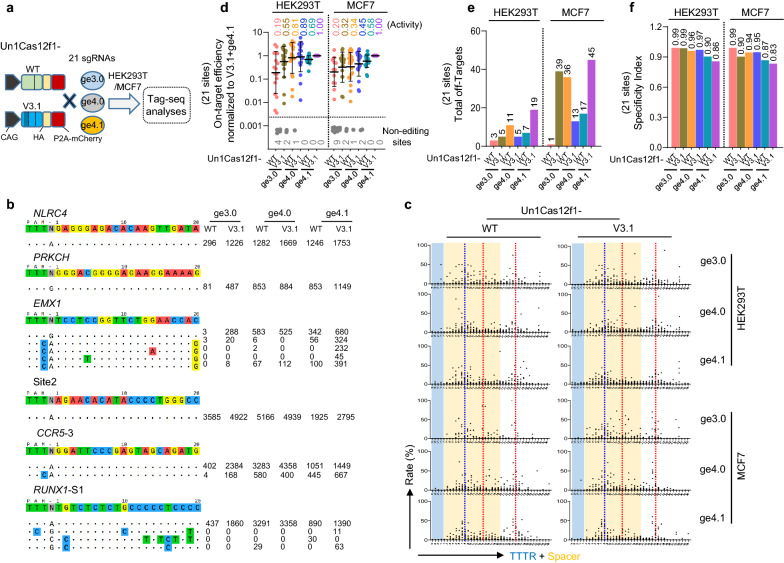
The performance of the CRISPR-Un1Cas12f1 systems in human cells.** a** Schematic of the editing ability analysis of the engineered CRISPR-Un1Cas12f1 systems, which contain two nucleases (also see Additional file 1: Fig. S1a) combined with three engineered sgRNA, ge3.0, ge4.0, and ge4.1. **b** Tag-seq-based comparative analysis of wild-type Un1Cas12f1 (WT), and Un1Cas12f1-V3.1 (V3.1, a reported high-active variant) combined with the engineered sgRNAs (ge3.0, ge4.0, and ge4.1) targeted to twenty-one sites in HEK293T cells (also see Additional file 1: Fig. S4). The sgRNA reference is shown on the top and the on-target and the off-target cleavages are displayed without or with mismatches to the sgRNA reference by color highlighting. Sequencing read counts are shown to the right of each site. **c** The characteristics of the Un1Cas12f1 induced double-strand-breaks (DSBs). Each point was calculated as the ratio of the read count at each break site to the total break read counts at this sgRNA, and then pooled within the twenty-one sgRNAs. Read counts were obtained from Tag-seq (Additional file 1: Fig. S5). x-axis shows the location of the sgRNA. The red dotted line indicates the reported in vitro DSB sites, while the blue dotted line indicates another potential breakpoint in vivo. **d** Normalization of on-target activity of the various CRISPR-Un1Cas12f1 systems to Un1Cas12f1-V3.1 + ge4.1 combination, value = (other system on-target reads)/(V3.1 + ge4.1 on-target reads). Grey points, sites without detectable editing. **e** Total number of off-target sites detected within the twenty-one sgRNAs. **f** Specificity Index assessment (value was calculated by the ratio of total on-target reads to the on-target reads plus the off-target reads within the twenty-one sites)

It has been reported that both protein engineering (V3.1 mutant of Un1Cas12f1, D143R/T147R/E151A/G297C) and sgRNA engineering (sgRNA variants, ge3.0, ge4.0, and ge4.1) could enhance gene editing of the Un1Cas12f1 system (Fig. 1a and Additional file 1: Fig. S1a–d) [8, 9]. However, the performance of their combinations has not been defined. The Tag-seq approach is a convenient and scalable method for genome-wide specificity assessment of CRISPR editors and can accurately identify and characterize Cas-induced double-strand breaks (DSBs) (Additional file 1: Fig. S1e) [13]. Thus, after confirming the comparable Un1Cas12f1-protein expression level by Western blotting (Additional file 1: Fig. S2), we employed Tag-seq to examine the editing abilities of the engineered Un1Cas12f1 combinations in HEK293T cells with twenty-one sgRNAs targeting eighteen genes (Fig. 1b and Additional file 1: Figs. S3, S4). Consistent with the previous in vitro assays [7], Tag-seq showed that Un1Cas12f1 induced two DSBs approximately at the region of 14th bp and 25th bp downstream of the TTTR (R = A/T) PAM (Fig. 1c and Additional file 1: Fig. S5). Surprisingly, the data also revealed that the region around the 8th bp could be a new cut site for in vivo editing (Fig. 1c), which was distinct from the reported in vitro assays [7]. Globally, the variant V3.1 Un1Cas12f1 displayed higher activity than WT Un1Cas12f1, and the engineered sgRNAs ge4.1 and ge4.0 were better than ge3.0 (Fig. 1d), both of which were consistent with the previous reports [8, 9]. In detail, the V3.1 combined with the ge4.1 sgRNA (V3.1 + ge4.1) displayed a robust editing ability, since it can edit all the twenty-one tested sites and was the most active combination. The V3.1 combined with the ge4.0 (V3.1 + ge4.0) performed better than that with the ge3.0 (V3.1 + ge3.0) (Fig. 1d). For the specificity, in line with the "trade-off" hypothesis that increased activity compromises specificity and *vice versa* [14, 15], the V3.1 + ge4.1 editor displayed the best efficiency but the lowest specificity, while the V3.1 + ge4.0 combination retained balanced in activity and specificity (Fig. 1d-f and Additional file 1: Fig. S4). Together, these data demonstrated that using a high-efficient Cas-nuclease variant combined with a better-engineered sgRNA could improve the performance of the Un1Cas12f1 system without compromised specificity. And similar results were observed in another cell line, MCF7 cells (Fig. 1d–f and Additional file 1: Fig. S5, S6). Thus, the V3.1 + ge4.1 and V3.1 + ge4.0 systems were selected for further study.

### Activity comparison of the DNA-targeting CRISPR editors

DNA-targeting CRISPR systems had been harnessed as versatile approaches for a variety of applications [1], however, these systems’ editing features, such as activity, specificity, cutting site, indel property, and transcriptional activation ability, have not been parallelly and comprehensively elucidated. Thus, we next focused on several common DNA-targeting CRISPR systems, including CRISPR-*Sp*Cas9 [16], CRISPR-Cas12a (*As*Cas12a and *Lb*Cas12a) [17], CRISPR-Un1Cas12f1 (V3.1 + ge4.1 and V3.1 + ge4.0) [8, 9], and CRISPR-*As*Cas12f1 (another Cas12f1 editor) [7, 10, 18, 19], to compare their gene-editing performance.

**Fig. 2:**
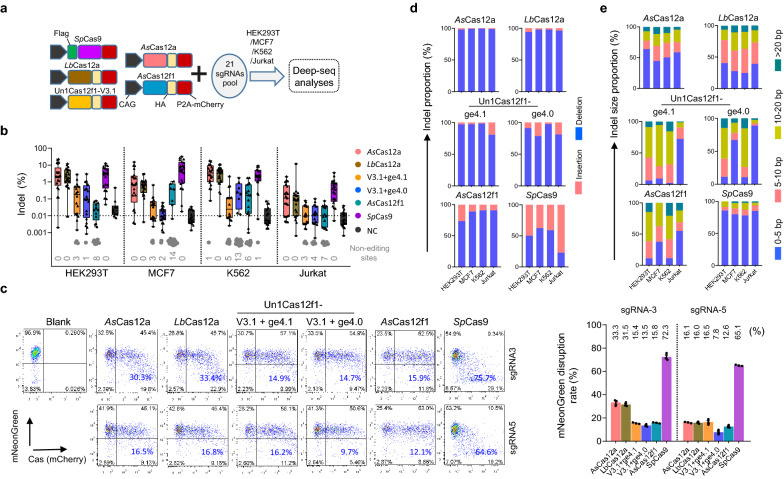
Activity comparison of the DNA targeting CRISPR systems in human cells.** a** Schematic of the editing performance analysis by Deep-seq among the DNA targeting CRISPR editors (CRISPR-*As*Cas12a, CRISPR-*Lb*Cas12a, CRISPR-Un1Cas12f1, CRISPR-*As*Cas12f1, and CRISPR-*Sp*Cas9 systems). **b** The editing activities of the DNA targeting systems with twenty-one sgRNAs in HEK293T, MCF7, K562, and Jurkat cells were revealed by Deep-seq (also see Additional file 1: Fig. S7). The targeted sgRNAs for Cas12 and Cas9 share a common spacer sequence. Grey points, sites without detectable editing. **c** FACS revealed the editing activities of the DNA targeting systems by disruption of mNeonGreen expression in a HEK293T-KI reporter cell line. The editing efficiency (values were shown with blue) was determined as the proportion of GFP negative cells within the Cas-nucleases transfected cells (mCherry-positive). n. sgRNA3/5, sgRNAs targeting mNeonGreen. Mean values are presented with SEM, n = 3 independent experiments (also see Additional file 11: Fig. S8, another two replicates). **d** The proportion of the insertions and deletions induced by the DNA targeting systems within the twenty-one sites. **e** The proportion of the indel size induced by the DNA targeting systems within the tewnty-one sites

First, we intended to examine the editing activities of these six DNA editors, because the editing efficiency was a key concern for a CRISPR-Cas gene-editing tool. After confirming the similar Cas-protein expression level and transfection efficiency (Additional file 1: Figs. S2, S3), the comparison was performed by Deep-seq experiment in HEK293T cells (Fig. 2a). To enable a better comparison of the on-target activities of the miniature Cas12f1 with that of widely-used Cas9 and Cas12a, we designed twenty-one endogenous human gene targeting sites that contained overlapping spacers for both Cas9 and Cas12a/Cas12f1 nucleases, where the sequences started with the TTTR motif (locations should be at − 1 to − 4) and ended with the NGG PAM (locations should be at + 20 to + 23). And these sites were chosen to have variable numbers of predicted off-target sites in the genome, as estimated using Cas-OFFinder [20] (Additional file 2: Table S1). Consistent with the recently reported study [21], Deep-seq results exhibited that the *Sp*Cas9 enzyme was the most active one in all the tested Cas-nucleases, followed by Cas12a, and then Cas12f1 (Fig. 2b and Additional file 1: Fig. S7). However, for the Un1Cas12f1, although the best engineered Un1Cas12f1/gRNA combination was used, its activity was much lower than that of *Sp*Cas9 and Cas12a (Fig. 2b). Consistently, the disruption of mNeonGreen expression in a HEK293T knock-in reporter cell line also revealed the poor editing activity of Un1Cas12f1 and *As*Cas12f1(Fig. 2c and Additional file 1: Fig. S8). These observations were quite different from the previous reports [9, 21]. We speculated that the possible reasons might come from the transfection method. In the recently reported work [21], the authors determined the editing efficiency within 2–7 days after 3 days of puromycin selection by single sgRNA transfection (thus the transfection efficiency was ~ 100%), while we calculated the cutting activity 2 days post-transfection by employing a scalable method that the experiment was administrated by pooling all the twenty-one sgRNAs within a single transfection (the total amount of the input DNA was the same as a single guide and the transfection efficiency was ~ 30–70%, Additional file 1: Fig. S3). In terms of inducing indels, Deep-seq data showed that *Sp*Cas9 was more likely to produce balanced insertions and deletions because it led to blunt end breaks [16, 22], while Cas12f1 and Cas12a tended to induce deletions since they generated sticky end breaks (Fig. 2d, e and Additional file 1: Fig. S7) [7–10, 17, 19, 23]. Apart from HEK293T cells, we also performed the experiments in other human cell lines, such as MCF7, K562, and Jurkat cells (Fig. 2b, d, e and Additional file 1: Fig. S7), and similar results were obtained.

### Specificity comparison of the DNA-targeting CRISPR editors

**Fig. 3:**
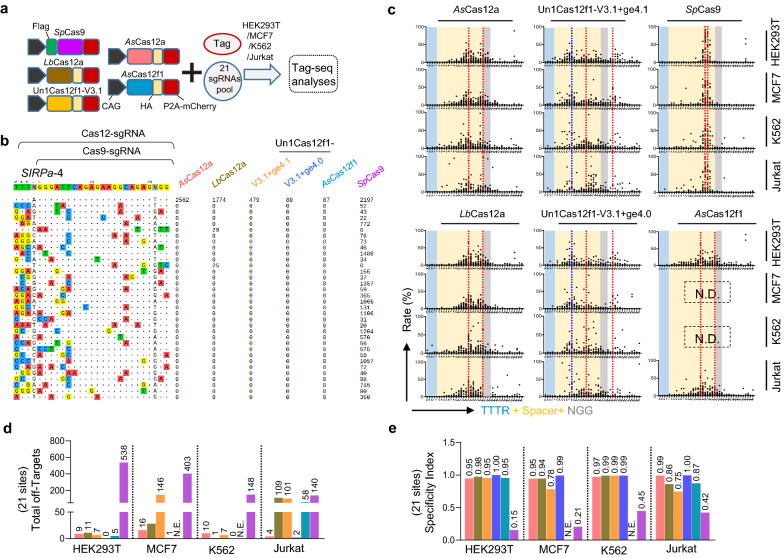
Specificity comparison of the DNA targeting CRISPR systems in human cells.** a** Schematic of the editing specificity analysis by Tag-seq among the DNA targeting CRISPR editors (CRISPR-*As*Cas12a, CRISPR-*Lb*Cas12a, CRISPR-Un1Cas12f1, CRISPR-*As*Cas12f1, and CRISPR-*Sp*Cas9 systems). **b** Tag-seq-based comparative analysis of DNA targeting CRISPR systems with twenty-one sgRNAs (also see Additional file 1: Figs. S9–S12). The targeted sites for Cas12 and Cas9 share a common spacer sequence as shown at the top. As for Cas12a/Cas12f1, the sgRNA reference is the full sequence. As for SpCas9, the sgRNA sequence begins after the TTTR and ends with its NGG PAM. **c** The characteristics of the DNA targeting CRISPR systems induced DSBs revealed by Tag-seq. x-axis showing the location of the sgRNA. The red dotted line indicates the expected DSB sites, while the blue dotted line indicates the new potential breakpoint. **d** Total number of off-target sites detected with the twenty-one sgRNAs. N.E., no editing detected. **e** Specificity Index assessment (value was calculated by the ratio of total on-target reads to the on-target reads plus the off-target reads within the twenty-one sites)

As the off-target effect is another major concern of the CRISPR-Cas systems for therapeutic applications, we then compared the targeting accuracy of these six editors. Thus we again performed Tag-seq assays with the above twenty-one sgRNAs to compare their specificities in genome-editing (Fig. 3a, b and Additional file 1: Figs. S3 and S9). As a result, for the cutting features, Tag-seq showed that the break sites induced by *Sp*Cas9 were at 3^rd^-4^th^ bp upstream of its NGG PAM, and that Cas12a (*As*Cas12a and *Lb*Cas12a) displayed multiple staggered breaks peaking at around 14th and 20th bp downstream of the TTTR PAM (Fig. 3c). Consistently, Un1Cas12f1 exhibited three potential breaks at around 8th, 14th, and 25th bp downstream of the TTTR PAM sequence (Fig. 3c). *As*Cas12f1 induced similar cleavage signals to Cas12a, approximately peaking at 14th and 20th bp, which was consistent with the previous report [10] (Fig. 3c). Among these six tested Cas-nucleases, Tag-seq data revealed that *Sp*Cas9 was much less specific than Cas12a and Cas12f1, which triggered hundreds of off-target cleavages at other loci (Fig. 3d, e), indicating a weakness in targeting accuracy, and this conclusion was also supported by the recently reported research that the Un1Cas121 and Cas12a had a higher editing safety [21]. Similar results were observed in MCF7, K562, and Jurkat cell lines (Fig. 3c-e and Additional file 1: Fig. S10–S12).

### Gene activation comparison of the DNA-targeting CRISPR editors

**Fig. 4:**
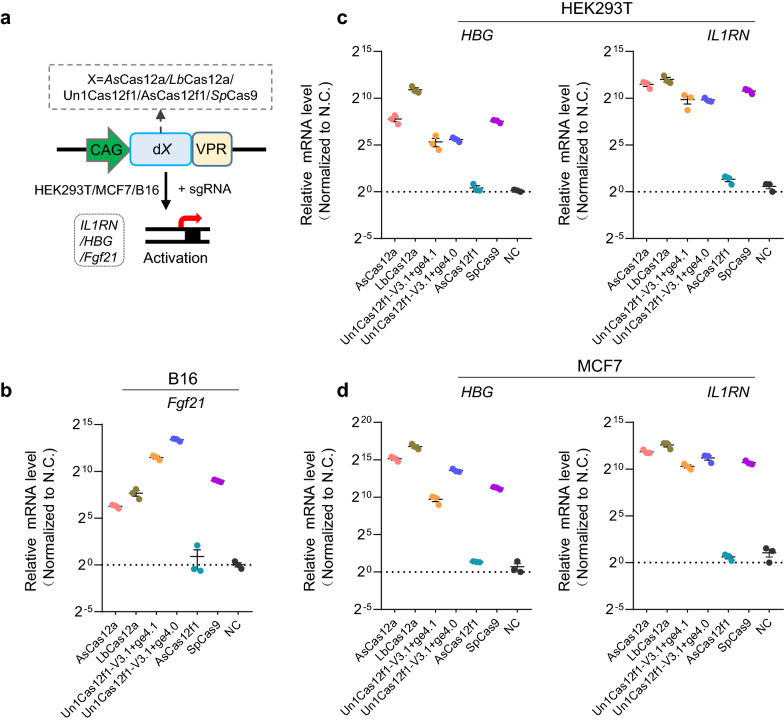
Gene activation comparison of the DNA targeting CRISPRa systems in mammalian cells. **a** Schematic of the gene activation system based on DNA targeting CRISPR systems. VPR, synthetic VP64-p65-Rta activation domain. **b–d** qPCR analyses of the transcriptional activation levels with DNA targeting CRISPRa guided by a single sgRNA targeting each promoter region of *Fgf21* mouse B16 cells (**b**), and *IL1RN* and *HBG* in human cells HEK293T (**c**) and MCF7 (**d**), respectively. Mean values are presented with SEM, n = 3 independent experiments

Since the CRISPR-based activation (CRISPRa) system is a useful technique, which holds great promise for clinical therapy applications [24–26], we finally determined the transcriptional activation abilities of these six CRISPRa activators. We constructed these DNA-targeting CRISPRa by fusing the DNase-inactive Cas-nuclease to the synthetic VPR (VP64-p65-Rta) activation domain and then tested their transcriptional activation of *IL1RN* and *HBG* in human cells HEK293T and MCF7, and *Fgf21* in mouse B16 cell line (Fig. 4a). As a result, we found that similar to the Cas12a and Cas9 CRISPRa systems, dUn1Cas12f-V3.1 combined with the ge4.1 or the ge4.0 sgRNA could induce *IL1RN*, *HBG, and Fgf21* expression with a comparable level in all the tested cells (Fig. 4b–d), which was consistent with the previous study [8], demonstrating its ability in transcriptional activation. Nevertheless, the dAsCas12f1-VPR system exhibited undetectable activity (Fig. 4b–d), indicating a low capacity for gene regulation, and thus further engineering for improvement was required. These data suggested that the miniature CRISPR-Un1Cas12f1 system was also a powerful CRISPRa platform, which could be an alternative tool for gene activation.

The hypercompact Cas-nucleases provide a great promise for programmable DNA modulation, especially for in vivo therapeutic applications. The Cas12f1 nuclease is one of these miniature enzymes, which can be easily packaged in an AAV vector for gene therapy delivery [9]. However, in terms of cleaving genomic DNA, our data and the recently reported study [21] together revealed that this editor’s efficiency was generally lower than that of the widely used Cas9 and Cas12a (Fig. 2b, c). Therefore, to promote and extend the applications of the miniature Cas12f1 nucleases in genomic cleavage, further engineering aiming to increase the activity of Un1Cas12f1, as well as *As*Cas12f1, is an urgent requirement. On the other hand, we also examined the transcription activation ability by using DNase-inactive Un1Cas12f1 (dUn1Cas12f1) fused with the transcription activator VPR. Consistent with the original report [8], our results showed that the Un1Cas12f1 nuclease could induce robust endogenous gene expression to a comparable extent with that of *Sp*Cas9 and Cas12a (Fig. 4), demonstrating its potential for gene-regulated therapy. Therefore, we highly recommend using these DNA-targeting editors according to the fitness of their unique properties to the intended scenarios. Generally, to disrupt genes in cell lines or animal models, we will recommend the *Sp*Cas9 enzyme since the *Sp*Cas9 is still the most active nuclease to date and the activity is generally a priority over the specificity in these experiments. In contrast, for the experiments related to disrupting genes for therapy purposes, we will recommend the Cas12a effectors since the specificity (and thus the safety) is a priority over the activity and the Cas12a nucleases retain a relatively high targeting accuracy and also display efficient editing ability. As for Cas12f1, we will recommend it to be used as an approach for gene activation-related therapy since it has a small size that can be easily packaged in the AAV vector and enable robust gene activation. For more detailed comparisons among these DNA-targeting editors, please refer to Table 1.

**Table 1:** Comparison of the commonly used DNA targeting CRISPR editors

	Cas9	Cas12a	Un1Cas12f1	AsCas12f1
PAM	NGG	TTTV	TTTR	NTTR
Size	SpCas9 (1368 aa)	AsCas12a (1307 aa); LbCas12a (1228 aa)	529 aa	422 aa
Cutting site	-3th, -4th bp	14th, 20th	8th, 14th, and 25th	14th, 20th
Break site	Blunt end	Sticky end	Sticky end	Sticky end
Specificity	+	+++	+++	+++
Activity	+++	+	+	+
CRISPR-based activation	+++	+++	+++	-
Application scenarios	Disrupt genes in cells or animal models	Disrupt genes for therapy purposes	Gene-activation-based therapy	Need to improve

## Conclusion

In summary, the parallel comparison of the commonly used DNA-targeting CRISPR editors provides basic information for the gene-editing toolkit, which can be a guideline and a helpful resource for this field.

## Methods

### Plasmids construction

All the Cas-nucleases expressing plasmids used in this study were constructed by standard PCR and molecular cloning into a plasmid containing a CAG promoter, a Flag/3xHA tag, a Cas-protein CDS expression cassette, and a P2A-mcherry reporter via Gibson Assembly. Except for the *Sp*Cas9 plasmid where the Flag tag was designed at its N-terminus, other vectors of Cas-protein used the 3xHA tags were constructed at their C- terminus. All sgRNAs targeting the genes of interest were designed through https://benchling.com/. sgRNA expression plasmids were constructed via digesting the sgRNA backbone plasmids that contained a human U6 promoter and a Cas-nuclease’s corresponding sgRNA scaffold sequences using BsmB I or Bbs I endonuclease (NEB) and then ligated the oligonucleotide duplexes into this cut backbone. The Cas-nuclease’s corresponding sgRNA scaffold sequences were listed in the Additional file 2. All the plasmids were confirmed by Sanger sequencing and all the sgRNAs oligonucleotides used in this study were shown in Additional file 2: Table S2.

### Cell culture

HEK293T and B16 cells were maintained in Dulbecco’s Modified Eagle’s Medium (DMEM, Life Technologies) at 37 °C in a 5% CO2 humidified incubator. MCF7, Jurkat, and K562 cells were maintained in RPMI 1640 medium (Life Technologies) at 37 °C in a 5% CO2 humidified incubator. All growth media were supplemented with 2 mM L-glutamine (Life Technologies), 100 U/mL penicillin, 100 µg/mL streptomycin (Life Technologies), and 10% fetal bovine serum. All the cell lines in this study were cultured in no more than 10 passages.

### Cell transfection and genomic DNA extraction

Generally, the HEK293T, MCF7, and B16 cells were transfected by the PEI method. For each transfection, approximately 2.0 × 10^5^/5.0 × 10^5^/1.0 × 10^6^cells were seeded in the 24/12/6-well plate, and the next day when cells grew up to 60% ~ 70%, the transfection was performed by PEI reagent with 250/500/1000 ng of Cas-nuclease expression plasmid and 250/500/1000 ng of the sgRNA-encoding plasmid per well in a 24/12/6-well plate. For the Jurkat and K562 cells, approximately 1.0 × 10^6^cells were electroporated by the Lonza Nucleofector Kit method with 1200 ng of Cas-nuclease expression plasmid and 800 ng of the sgRNA-encoding plasmids for each test. All cells in each well were harvested 3 days post-transfection and the genomic DNA was extracted by TIANamp Genomic DNA Kit (TIANGEN Biotech Co., Ltd., Beijing, China) following the manufacturer’s instructions.

### Quantitative real‑time PCR

Total RNA from the transfected cells was isolated using Trizol Reagent (Thermo Fisher, USA) following the manufacturer’s instructions. Total RNA (1 μg) was reverse transcribed into cDNA and then quantitative real-time PCR was performed using a LightCycler 96 System (Roche, Switzerland). Relative gene expression was calculated using the 2^−ΔΔCt^ method after normalizing to GAPDH expression. The activation sgRNA used in this study and the qPCR primers were listed in Additional file 2: Table S2.

### Tag-seq analysis

Tag-seq can parallelly profile the off-target cleavages induced by Cas-nuclease at various sites in a single experiment and thus is a convenient and cost-efficient method for comparing the specificity among different nucleases, which was performed and described as previously reported [13, 27]. Briefly, for the HEK293T and MCF7, ~ 6.0 × 10^5^ cells were transfected by PEI with 10 pmol Tag, 600 ng of Cas nuclease, and 600 ng pooled sgRNAs (21 guides) per well in a 12-well plate. For the Jurkat and K562, ~ 1.0 × 10^6^ cells were transfected by Amaxa Cell Line Nucleofector Kit V (VCA-1003, Lonza, Switzerland) following the manufacturer’s instructions (2D) with 20 pmol Tag, 1200 ng of Cas nuclease, and 1000 ng pool sgRNAs (21 guides). Three days post-transfection genomic DNA was extracted for one-step libraries preparation by the Fragmentation, End Preparation, and dA-Tailing Module and Adapter Ligation Module kit (Vazyme Biotech Co., Ltd., Nanjing, China). The L and R libraries were constructed by PCR with library preparation primers, which were followed by sequencing (NovaSeq platform, Novogene, Beijing, China) and analysis with a Tag-seq bioinformatics pipeline. To profile the off-target effects induced by the Cas-nuclease at 21 sites in parallel, the 21 sgRNA sequences were parallelly listed in a format of “sgRNA-nameTTTNN_20_NGGTTTN” in the “sgrna. lst” file and the parameter in “config. docker. text” file was set as follows:

# parameter for detecting potential cutting sites.

MinSupportReadCount, 1; MinCuttingEventCount, 2

# off-target detection parameters.

MaxMismatch, 6; MaxGap, 1; MaxGapMismatch, 3.

The Tag-seq pipeline is available at https://github.com/zhoujj2013/Tag-seq and https://doi.org/10.5281/zenodo.4679460.

### Deep-seq analysis

Deep-seq was used to assess the editing activities of the DNA-targeting CRISPR editors. For HEK293T and MCF7, cells were transfected by PEI with 600 ng of Cas nuclease, and 600 ng pool sgRNAs (21 guides) per well in a 12-well plate. For the Jurkat and K562, cells were transfected by Amaxa Cell Line Nucleofector Kit V (VCA-1003, LONZA, Switzerland) following the manufacturer’s instructions (2D) with 1200 ng of Cas nuclease, and 1000 pool sgRNAs (21 guides). Two days post-transfection genomic DNA was extracted for deep-seq libraries preparation. Briefly, the primers with forward and reverse indexes were used to amplify the genomic regions in the first-round PCR. Then, equal amounts of the first PCR products were mixed and subjected to a second round of PCR with the P5- and P7-containing primers to generate the sequencing libraries. Paired-end sequencing was performed using the NovaSeq platform (Novogene, Beijing, China). Indel frequency was calculated as the ratio of (read counts with indel sequence)/(total sequencing read counts). The deep-seq primers were listed in Additional file 2: Table S3.

### Western blotting

For detecting the expression of the Cas-proteins, HEK293T cells were transfected with the Cas-protein encoding plasmids using the PEI method. 2 days post-transfection cells were harvested and lysed in a 2 × SDS loading buffer, and then boiled for 10 min. Lysates were resolved through SDS/PAGE and transferred onto a nitrocellulose membrane which was blocked using 5% non-fat milk and sequentially incubated with primary antibodies (anti-HA or anti-Flag, sigma, USA, anti-GADPH, Proteintech, China) and an HRP-conjugated horse anti-mouse IgG secondary antibody (CST, USA). All the probed proteins were finally detected through chemiluminescence following the manufacturer’s instructions (Pierce, USA).

### FACS analysis

All flow cytometry analyses were performed using FlowJo software (TreeStar, USA). To detect transfection efficiency, cells were harvested at the ending time and were determined as the proportion of the mCherry-positive (Cas-protein-P2A-mCherry cassette). To compare the editing activities of the DNA-targeting editors, cells were harvested two days post-transfection and the cleavage efficiency was determined as the proportion of GFP negative cells within the Cas-nucleases transfected cells (mCherry-positive).

### Activity and specificity scoring

For the comparison of the performance among CRISPR-Cas systems, Tag-seq reads were used for calculating the targeting specificity and the editing activity, which was analyzed similarly to our previous study [27]. For the engineered CRISPR-Un1Cas12a systems comparison, editing activity scores were calculated as the mean ratio of the on-target reads across all the tested sites, normalized to the V3.1 + ge4.1 combination. The specificity Index was calculated as the ratio of the on-target reads to the on-target reads plus the off-target reads across all the tested sites.

## Supplementary Information

**Additional file 1: Figure S1.** The schematic of the engineered CRISPR-Un1Cas12f1 system and the Tag-seq method. a The structures of the Un1Cas12f1 plasmids, where the sequences of the Un1Cas12f1-nucleases contained the G297C mutation was from the previous report^8^. b-d The structures and detailed sequences of the engineered sgRNAs, in which the colored sequences were the differences among these three sgRNAs. The ge4.0-sgRNA (c) was generated by the deletion of the red sequences in ge3.0-sgRNA (b), and the ge4.1-sgRNA (d) was generated by the deletion of the blue sequences in ge4.0-sgRNA. e The workflow of Tag-seq, a streamlined sequencing method, which has a broad spectrum of applications, like tracing DNA double-strand breaks induced by CRISPR tools, profiling CRISPR-based off-targets, evaluation of gene editing events, and profiling molecular characteristics in on- and off-target sites etc. **Figure S2.** The expression of the Cas nucleases. Western blot showing the expression levels of the Cas-protein nucleases. Exception for the SpCas9 that fusing with the anti-Flag at the N-terminus, the other nuclease were detected by the anti-HA which fused to the C-terminus. Blank, HEK293T without transfection. **Figure S3.** Detection of the transfection efficiency by FACS. The transfection were administrated by PEI-based method (for HEK293T and MCF7 cells) and Lonza kit (2D, for Jurkat and K562 cells) with the plasmids expression of Cas-protein (fusing a P2A mCherry reporter) and the pooled sgRNAs, and an Tag-oligo DNA, and the transfection efficiency was determined by FACS with the mCherry reporter. **Figure S4.** Specificity comparison of the engineered CRISPR-Un1Cas12f1 system by Tag-seq in HEK293T cells. HEK293T cells were transfected by PEI method with the plasmids expressing Un1Cas12f1-WT or -V3.1 and a pooled twenty-one sgRNAs (containing the ge3.0, ge4.0, or ge4.1), and the Tag-oligo DNA sequence. Genomic DNA was harvested three days post-transfection for libraries construction and Tag-seq analysis. Read counts represented a measure of cleavage frequency at a given site, mismatched positions within the spacer or PAM are highlighted in color (also see Fig. 1b). **Figure S5.**Characteristics of the distributions of Tag-oligo integration at break sites induced by DNA-targeting CRISPR systems in various cell lines.Sequencing reads are mapped back to the reference (Human hg19) for visualization of the localization of the break sites.The targeted sequences are shown with the spacer sequence downstream from the TTTR PAM site on the x axis. **Figure S6.** Specificity comparison of the engineered CRISPR-Un1Cas12f1 system by Tag-seq in MCF7 cells. MCF7 cells were transfected by PEI method with the plasmids expressing Un1Cas12f1-WT or -V3.1 and a pooled twenty-one sgRNAs (containing the ge3.0, ge4.0, or ge4.1), and the Tag-oligo DNA sequence. Genomic DNA was harvested three days post-transfection for libraries construction and Tag-seq analysis. Read counts represented a measure of cleavage frequency at a given site, mismatched positions within the spacer or PAM are highlighted in color. **Figure S7.** Activity comparison of the DNA editing CRISPR system by Deep-seq in 4 cell lines. Deep-seq revealed the editing activities of the DNA CRISPR editors by targeting twenty-one sites in various cell lines, including HEK293T, MCF7, K562, and Jurkat. Cells were transfected with the plasmids expressing DNA-editing nucleases, the corresponding sgRNAs that pooled with twenty-one guides. Two days post-transfection genomic DNA was harvested for Deep-seq libraries construction and editing efficiency analyses. **Figure S8.** Activity comparison of the DNA editing CRISPR system by FACS in HEK293T-KI-mNeonGreen reporter cells. Another two replicates for the Fig. 3c. The editing efficiency (values were showed with blue) was determined as the proportion of GFP negative cells within the Cas-nucleases transfected cells (mCherry-positive) by FACS. mNeonGree-sgRNA3/5, DNA editing CRISPR targeting mNeonGreen site 3/5. Red sequences showing the PAM of Cas12f/Cas12a, Blue sequences showing the PAM of Cas9. n=3 independent experiments. **Figure S9.** Specificity comparison of the DNA editing CRISPR systems in HEK293T cells. HEK293T cells were transfected by PEI method with the plasmids expressing DNA-editing nucleases, the corresponding sgRNAs that pooled with twenty-one guides, and the Tag-oligo DNA sequence. Genomic DNA was harvested three days post-transfection for libraries construction and Tag-seq analysis. Read counts represented a measure of cleavage frequency at a given site, mismatched positions within the spacer or PAM are highlighted in color (also see Fig. 2b). The targeted sites for Cas12a, Cas12f1 and SpCas9 share with a common spacer sequence as shown in the top. **Figure 10.** Specificity comparison of the DNA editing CRISPR systems in MCF7 cells. MCF7 cells were transfected by PEI method with the plasmids expressing DNA-editing nucleases, the corresponding sgRNAs that pooled with twenty-one guides, and the Tag-oligo DNA sequence. Genomic DNA was harvested three days post-transfection for libraries construction and Tag-seq analysis. Read counts represented a measure of cleavage frequency at a given site, mismatched positions within the spacer or PAM are highlighted in color. The targeted sites for Cas12a, Cas12f1 and SpCas9 share with a common spacer sequence as shown in the top. **Figure 11.** Specificity comparison of the DNA editing CRISPR systems in K562 cells. K562 cells were co-transfected by Lonza electroporation method with the plasmids expressing DNA-editing nucleases, the corresponding sgRNAs that pooled with twenty-one guides, and the Tag-oligo DNA sequence. Genomic DNA was harvested three days post-transfection for libraries construction and Tag-seq analysis. Read counts represented a measure of cleavage frequency at a given site, mismatched positions within the spacer or PAM are highlighted in color. The targeted sites for Cas12a, Cas12f1 and SpCas9 share a common spacer sequence as shown in the top. **Figure S12.** Specificity comparison of the DNA editing CRISPR systems in Jurkat cells. Jurkat cells were transfected by Lonza electroporation method with the plasmids expressing DNA-editing nucleases, the corresponding sgRNAs that pooled with twenty-one guides, and the Tag-oligo DNA sequence. Genomic DNA was harvested three days post-transfection for libraries construction and Tag-seq analysis. Read counts represented a measure of cleavage frequency at a given site, mismatched positions within the spacer or PAM are highlighted in color. The targeted sites for Cas12a, Cas12f1 and SpCas9 share a common spacer sequence as shown in the top.

**Additional file 2: Table S1.** Summary of potential mismatched sites in the reference human genome for the 21sgRNAs examined by Tag-seq. **Table S2.** The sgRNAs and primers used in this study. **Table S3.** Deep-seq primers for this study.

## Data Availability

All the next-generation sequencing data related to this study have been deposited in NCBI (Bioproject PRJNA799250). And other data that support the findings of this study are available from the corresponding author upon reasonable request.
