# Supplementary material for: Comparison of DNA targeting CRISPR editors in human cells
Source: Cell Biosci. 2023 Jan 16;13:11. doi: 10.1186/s13578-023-00958-z (PMC9844007; doi:10.1186/s13578-023-00958-z)
Supplement: Supplementary file 1 — Additional file 1: Figure S1. The schematic of the engineered CRISPR-Un1Cas12f1 system and the Tag-seq method. a The structures of the Un1Cas12f1 plasmids, where the sequences of the Un1Cas12f1-nucleases contained the G297C mutation was from the previous report8. b-d The structures and detailed sequences of the engineered sgRNAs, in which the colored sequences were the differences among these three sgRNAs. The ge4.0-sgRNA (c) was generated by the deletion of the red sequences in ge3.0-sgRNA (b), and the ge4.1-sgRNA (d) was generated by the deletion of the blue sequences in ge4.0-sgRNA. e The workflow of Tag-seq, a streamlined sequencing method, which has a broad spectrum of applications, like tracing DNA double-strand breaks induced by CRISPR tools, profiling CRISPR-based off-targets, evaluation of gene editing events, and profiling molecular characteristics in on- and off-target sites etc. Figure S2. The expression of the Cas nucleases. Western blot showing the expression levels of the Cas-protein nucleases. Exception for the SpCas9 that fusing with the anti-Flag at the N-terminus, the other nuclease were detected by the anti-HA which fused to the C-terminus. Blank, HEK293T without transfection. Figure S3. Detection of the transfection efficiency by FACS. The transfection were administrated by PEI-based method (for HEK293T and MCF7 cells) and Lonza kit (2D, for Jurkat and K562 cells) with the plasmids expression of Cas-protein (fusing a P2A mCherry reporter) and the pooled sgRNAs, and an Tag-oligo DNA, and the transfection efficiency was determined by FACS with the mCherry reporter. Figure S4. Specificity comparison of the engineered CRISPR-Un1Cas12f1 system by Tag-seq in HEK293T cells. HEK293T cells were transfected by PEI method with the plasmids expressing Un1Cas12f1-WT or -V3.1 and a pooled twenty-one sgRNAs (containing the ge3.0, ge4.0, or ge4.1), and the Tag-oligo DNA sequence. Genomic DNA was harvested three days post-transfection for libraries construction [file 13578_2023_958_MOESM1_ESM.pdf]

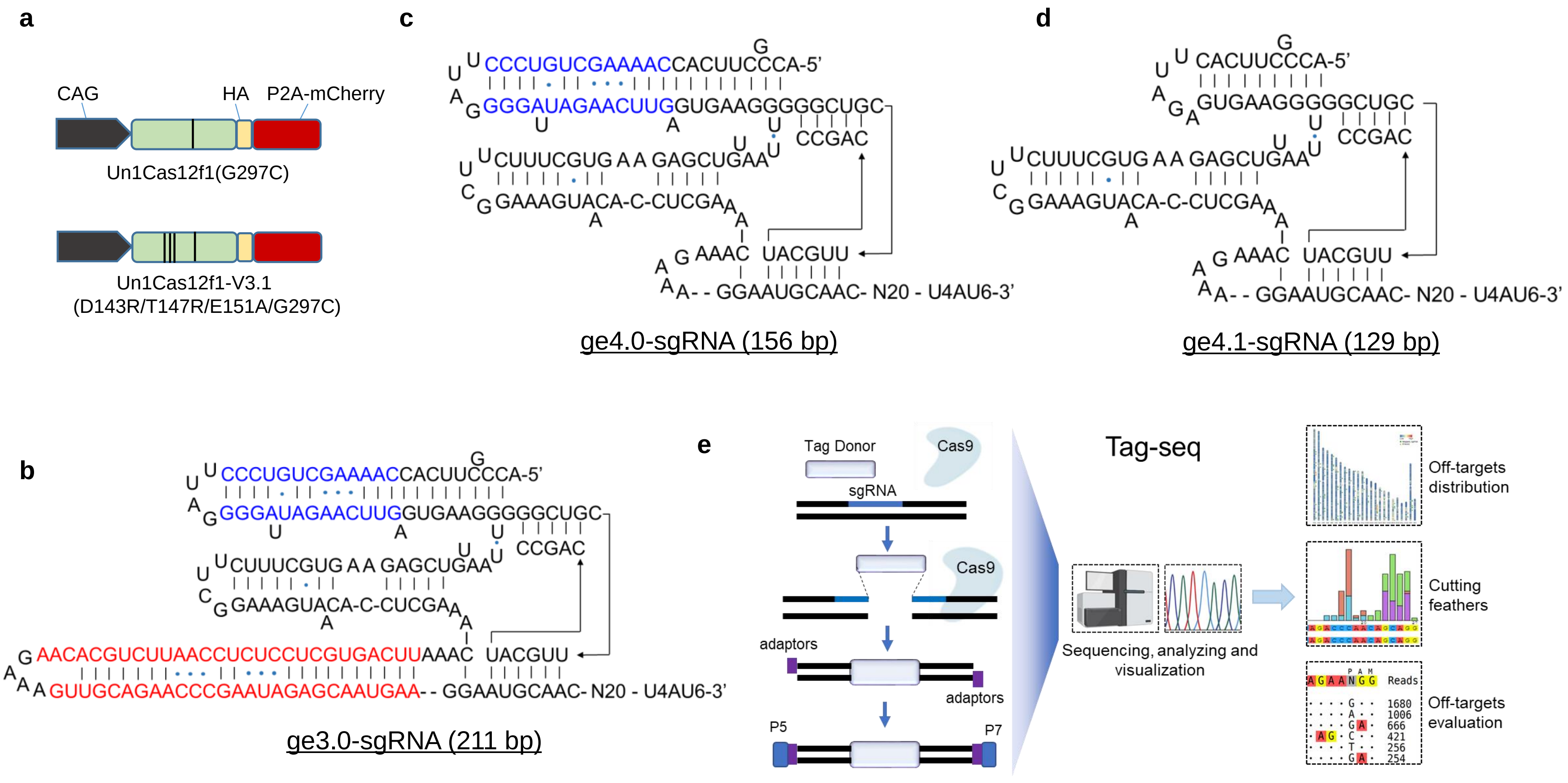

Fig. S1 The schematic of the engineered CRISPR-Un1Cas12f1 system and the Tag-seq method

Supplementary Figure 1. The schematic of the engineered CRISPR-Un1Cas12f1 system and the Tag-seq method. a The structures of the Un1Cas12f1 plasmids, where the sequences of the Un1Cas12f1-nucleases contained the G297C mutation was from the previous report[8]. **b-d** The structures and detailed sequences of the engineered sgRNAs, in which the colored sequences were the differences among these three sgRNAs. The ge4.0-sgRNA (c) was generated by the deletion of the red sequences in ge3.0-sgRNA (b), and the ge4.1-sgRNA (d) was generated by the deletion of the blue sequences in ge4.0-sgRNA. **e** The workflow of Tag-seq, a streamlined sequencing method, which has a broad spectrum of applications, like tracing DNA double-strand breaks induced by CRISPR tools, profiling CRISPR-based off-targets, evaluation of gene editing events, and profiling molecular characteristics in on- and off-target sites *etc*.

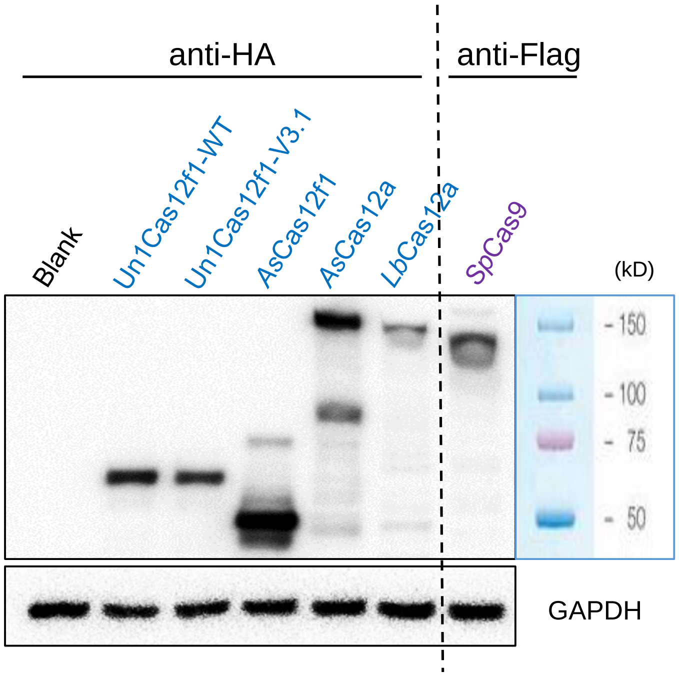

Fig. S2 The expression of the Cas nucleases

Supplementary Figure 2. The expression of the Cas nucleases. Western blot showing the expression levels of the Cas-protein nucleases. Exception for the *Sp*Cas9 that fusing with the anti-Flag at the N-terminus, the other nuclease were detected by the anti-HA which fused to the C-terminus. Blank, HEK293T without transfection.

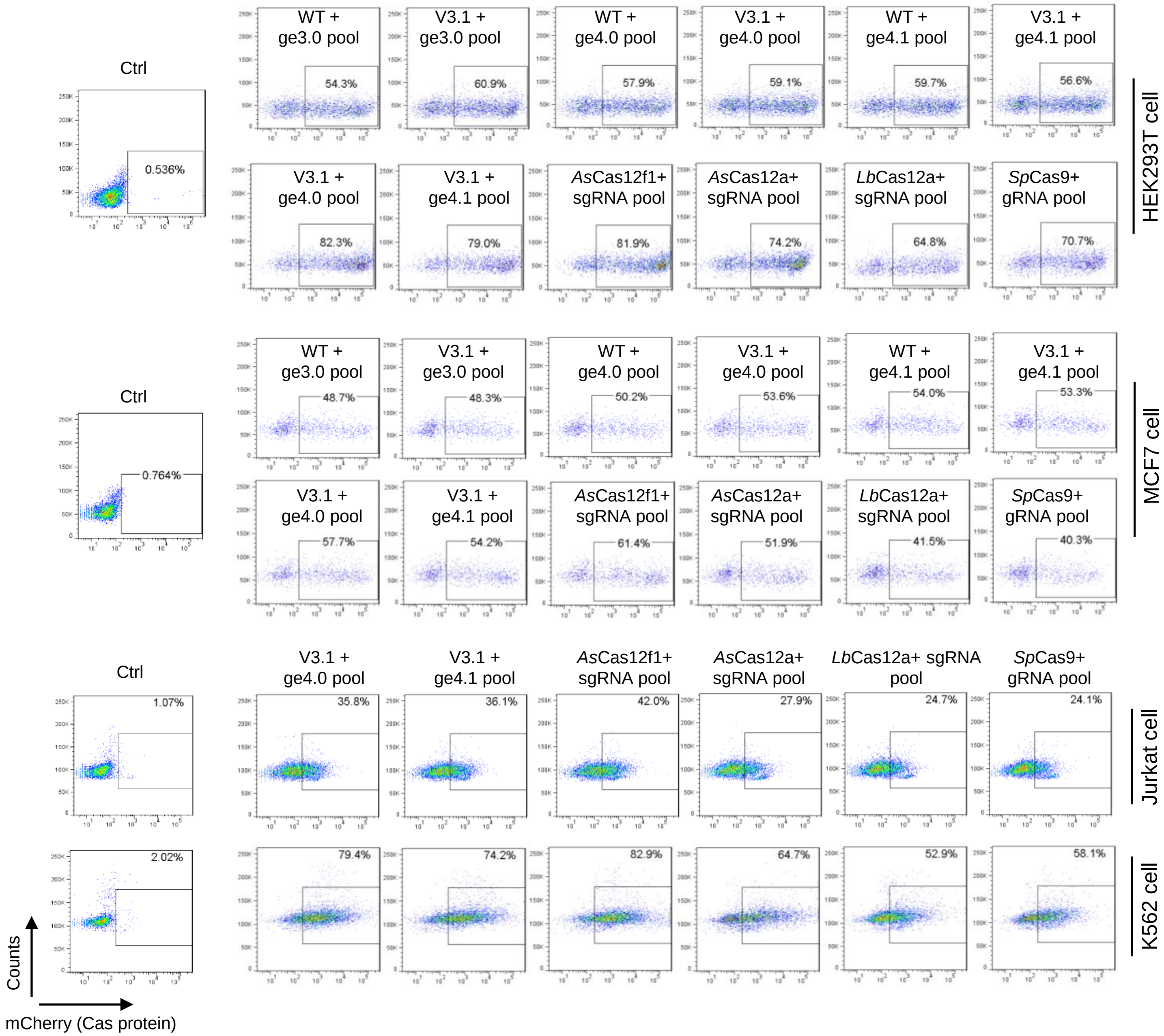

Fig. S3 Detection of the transfection efficiency by FACS

Supplementary Figure 3. Detection of the transfection efficiency by FACS. The transfection were administrated by PEI-based method (for HEK293T and MCF7 cells) and Lonza kit (2D, for Jurkat and K562 cells) with the plasmids expression of Cas-protein (fusing a P2A mCherry reporter) and the pooled sgRNAs, and an Tag-oligo DNA, and the transfection efficiency was determined by FACS with the mCherry reporter.

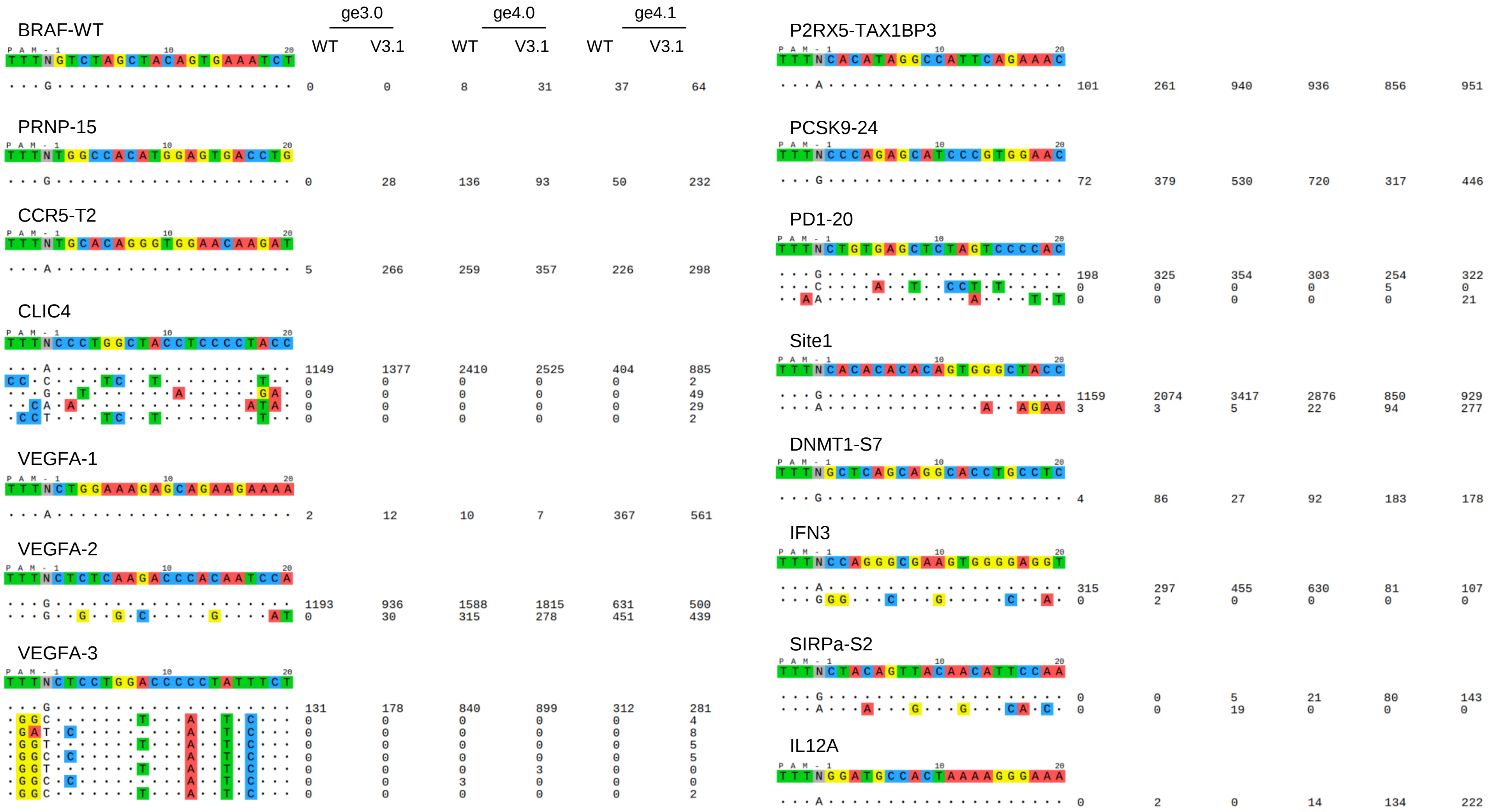

Fig. S4 Specificity comparison of the engineered CRISPR-Un1Cas12f1 systems by Tag-seq in HEK293T cells

Supplementary Figure 4. Specificity comparison of the engineered CRISPR-Un1Cas12f1 system by Tag-seq in HEK293T cells. HEK293T cells were transfected by PEI method with the plasmids expressing Un1Cas12f1-WT or -V3.1 and a pooled twenty-one sgRNAs (containing the ge3.0, ge4.0, or ge4.1), and the Tag-oligo DNA sequence. Genomic DNA was harvested three days post-transfection for libraries construction and Tag-seq analysis. Read counts represented a measure of cleavage frequency at a given site, mismatched positions within the spacer or PAM are highlighted in color (also see Fig. 1b).

Supplementary Figure 5

Characteristics of the distributions of Tag-oligo integration at break sites induced by DNA-targeting CRISPR systems in various cell lines.

Sequencing reads are mapped back to the reference (Human hg19) for visualization of the localization of the break sites.

The targeted sequences are shown with the spacer sequence downstream from the TTTR PAM site on the x axis.

CRISPR-Un1Cas12f1 systems (HEK293T cell)

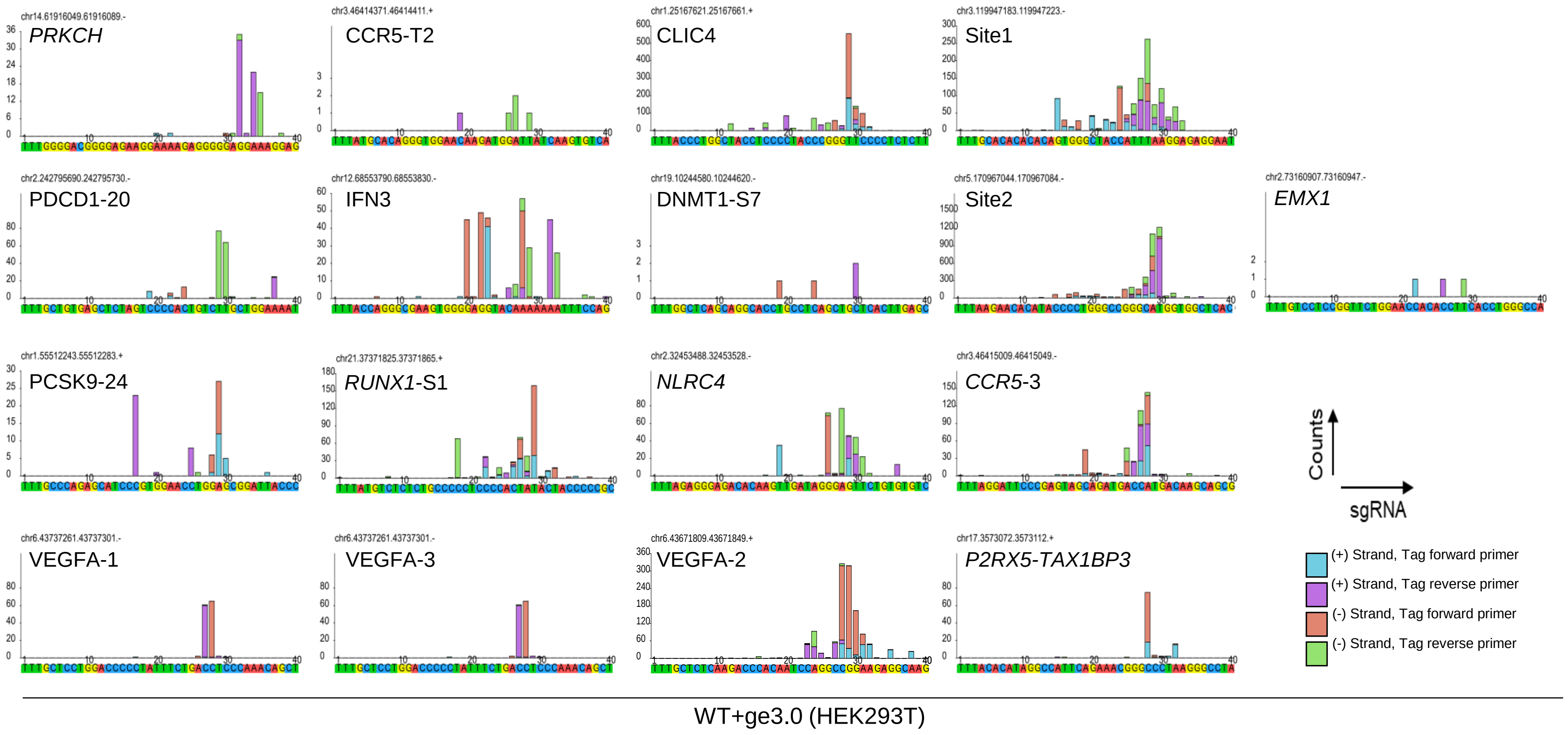

CRISPR-Un1Cas12f1 systems (HEK293T cell)

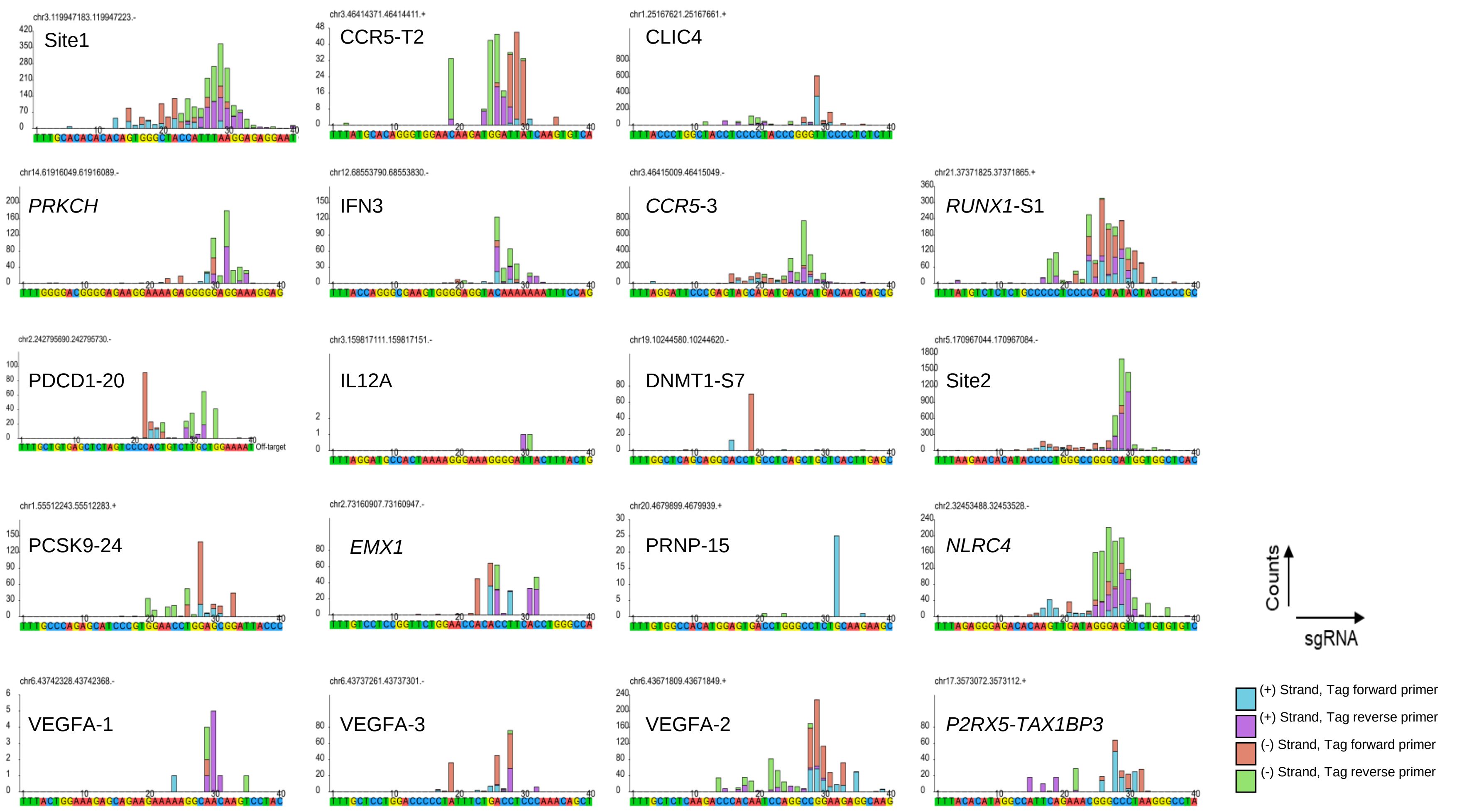

V3.1+ge3.0 (HEK293T)

CRISPR-Un1Cas12f1 systems (HEK293T cell)

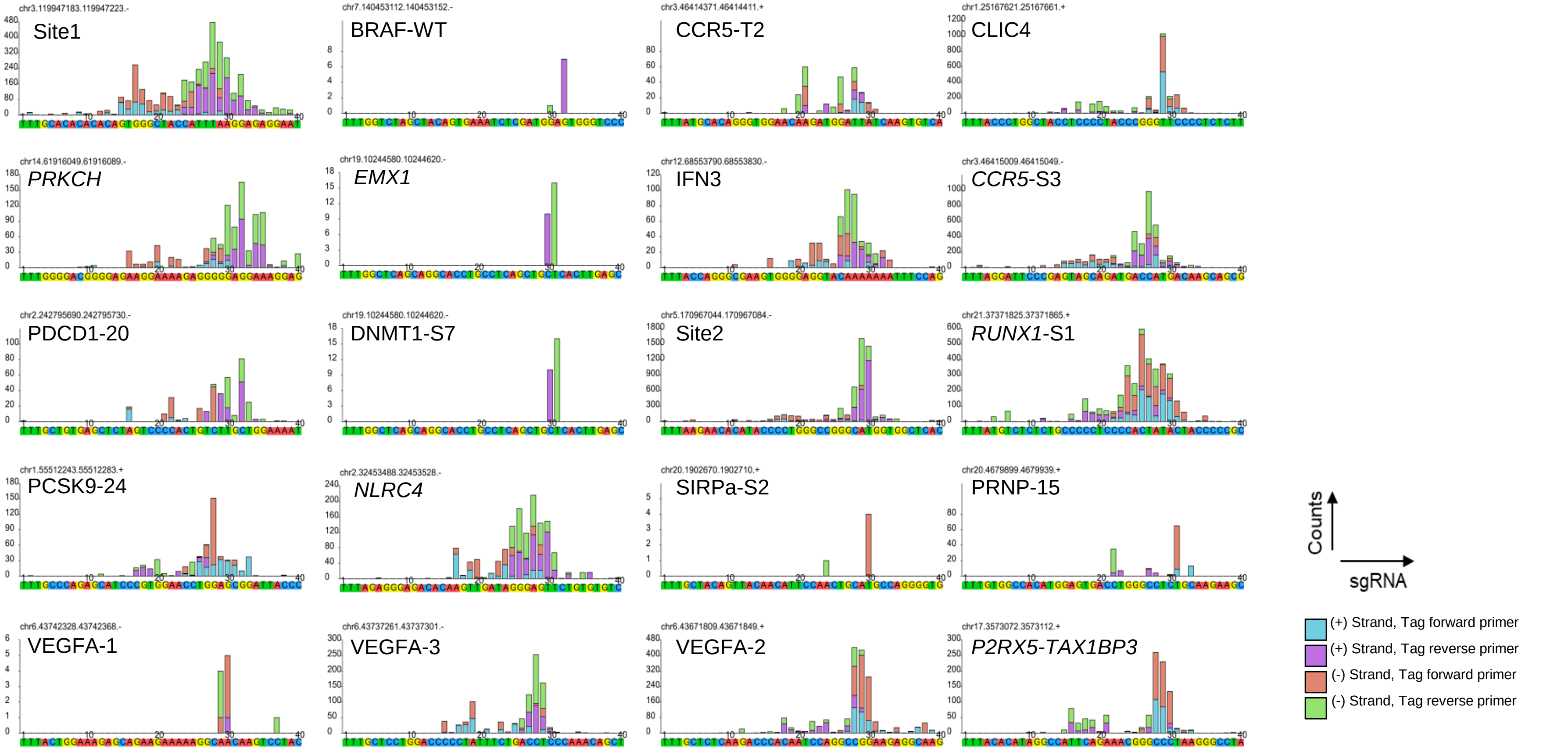

WT+ge4.0 (HEK293T)

CRISPR-Un1Cas12f1 systems (HEK293T cell)

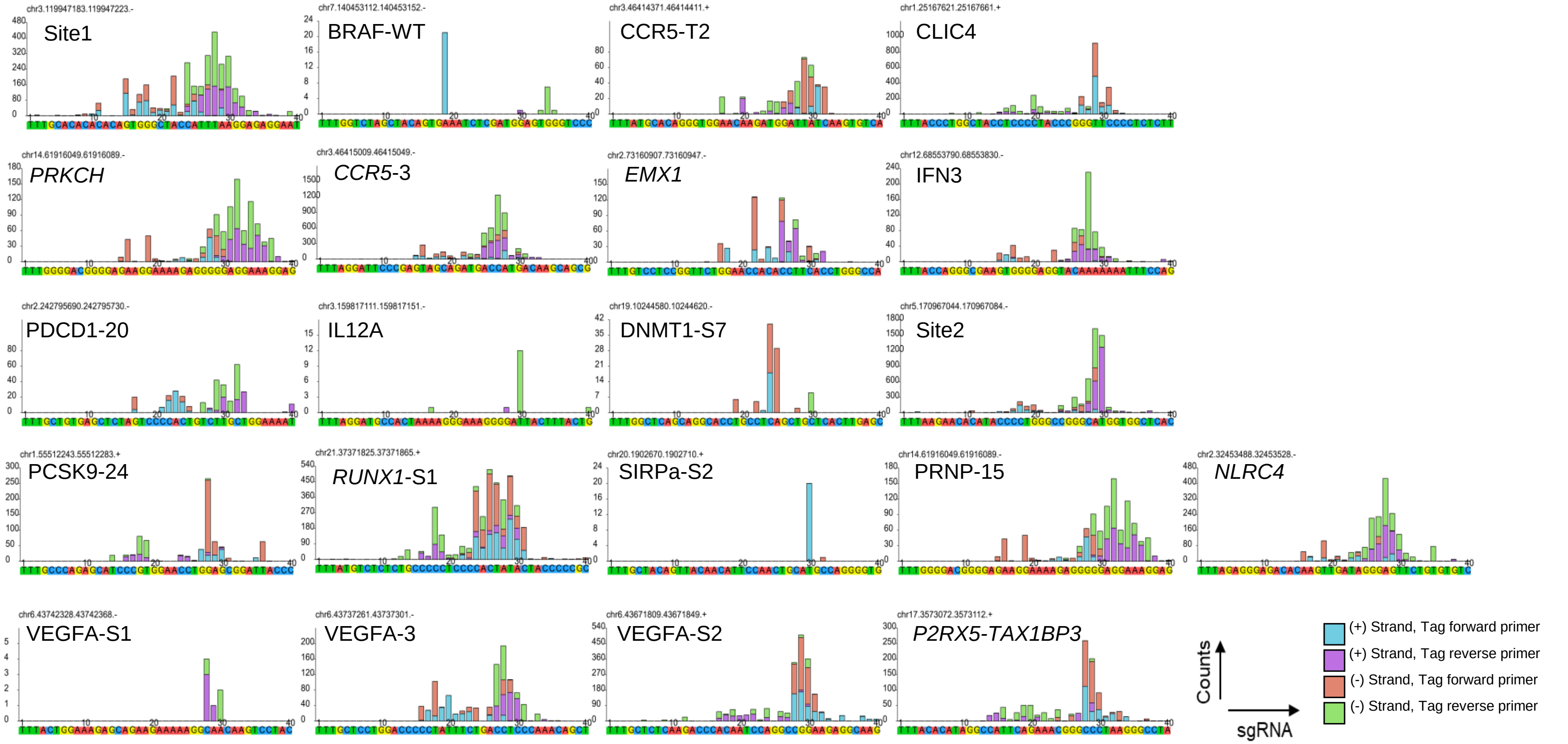

V3.1+ge4.0 (HEK293T)

CRISPR-Un1Cas12f1 systems (HEK293T cell)

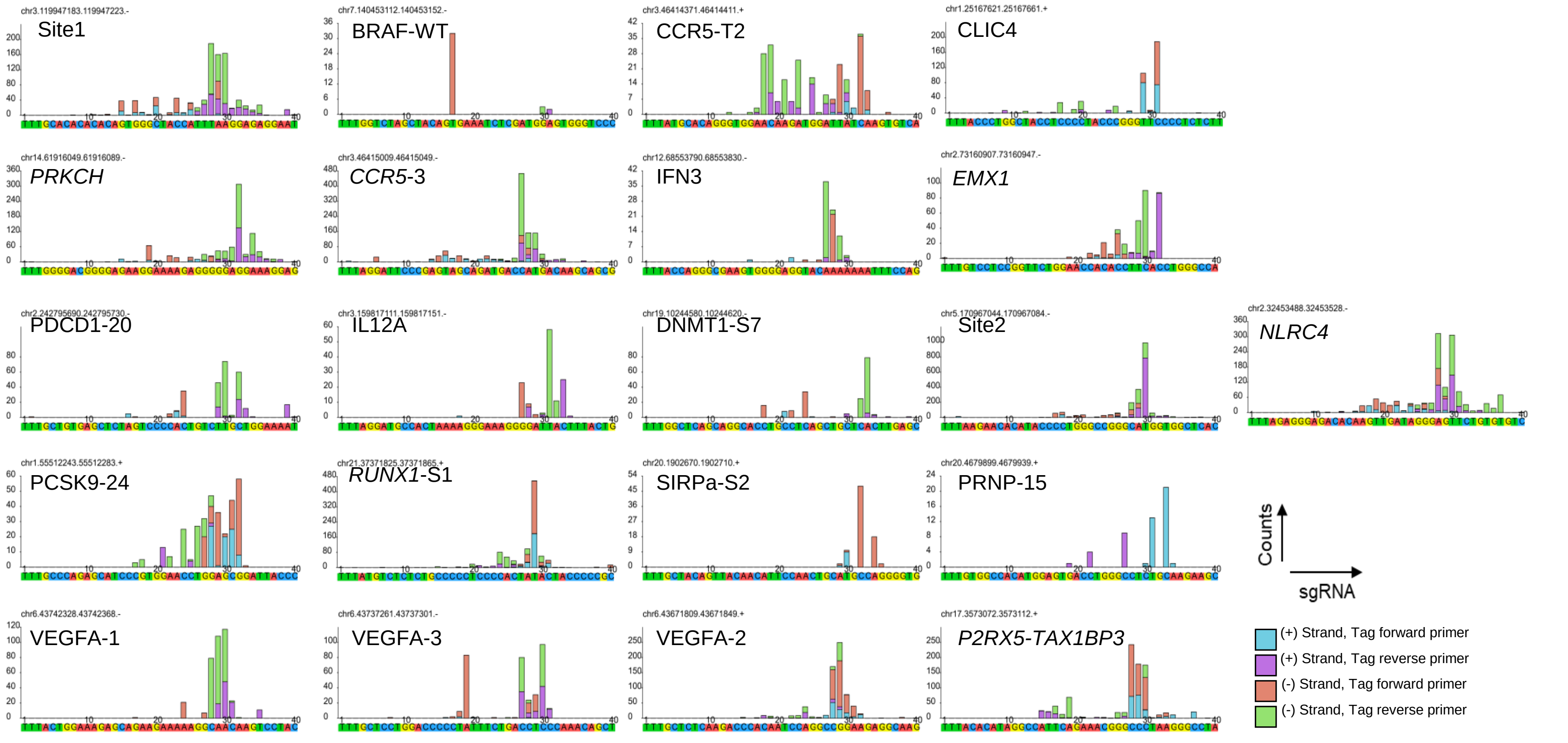

WT+ge4.1 (HEK293T)

CRISPR-Un1Cas12f1 systems (HEK293T cell)

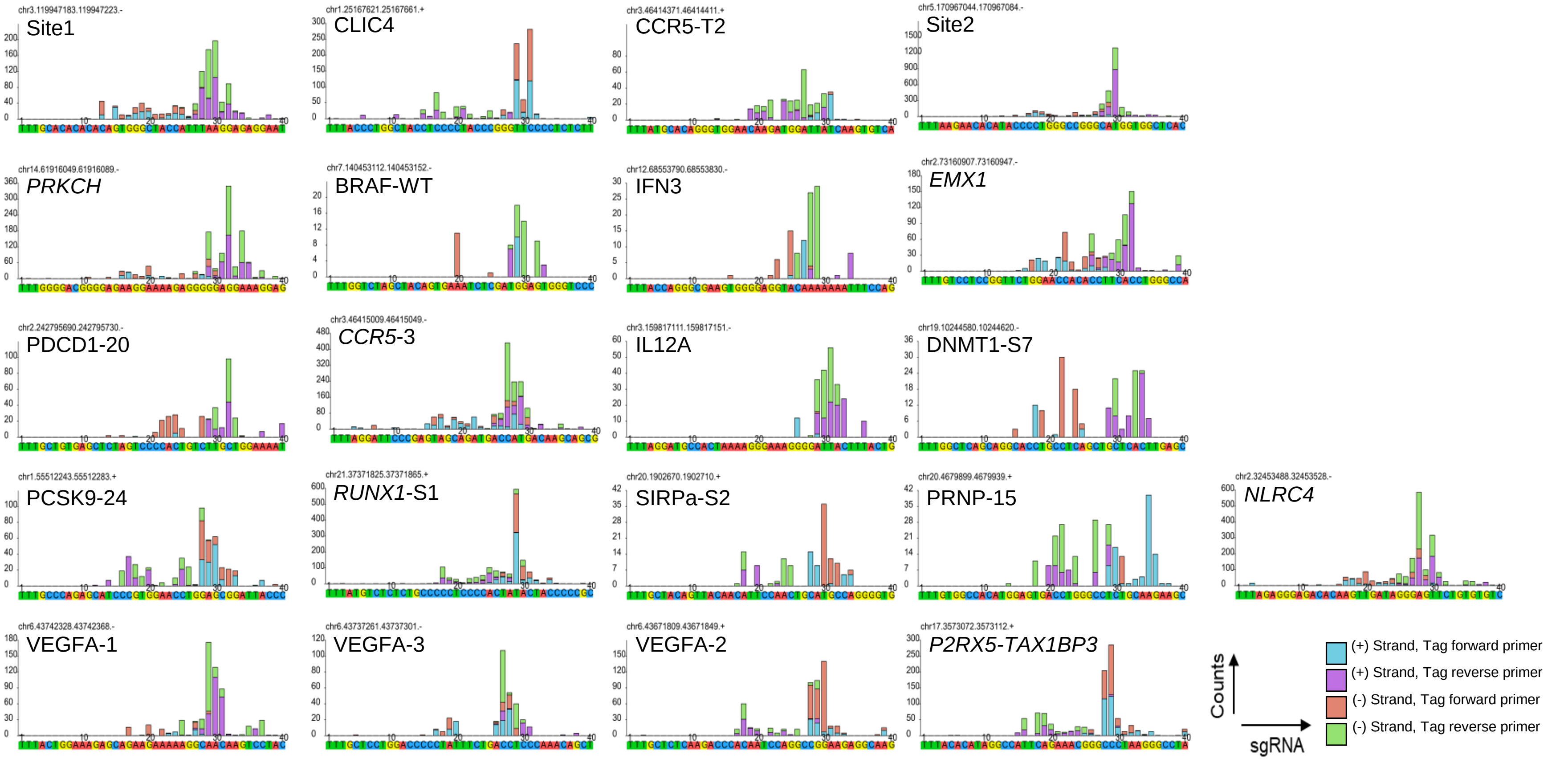

V3.1+ge4.1 (HEK293T)

CRISPR-Un1Cas12f1 systems (MCF7 cell)

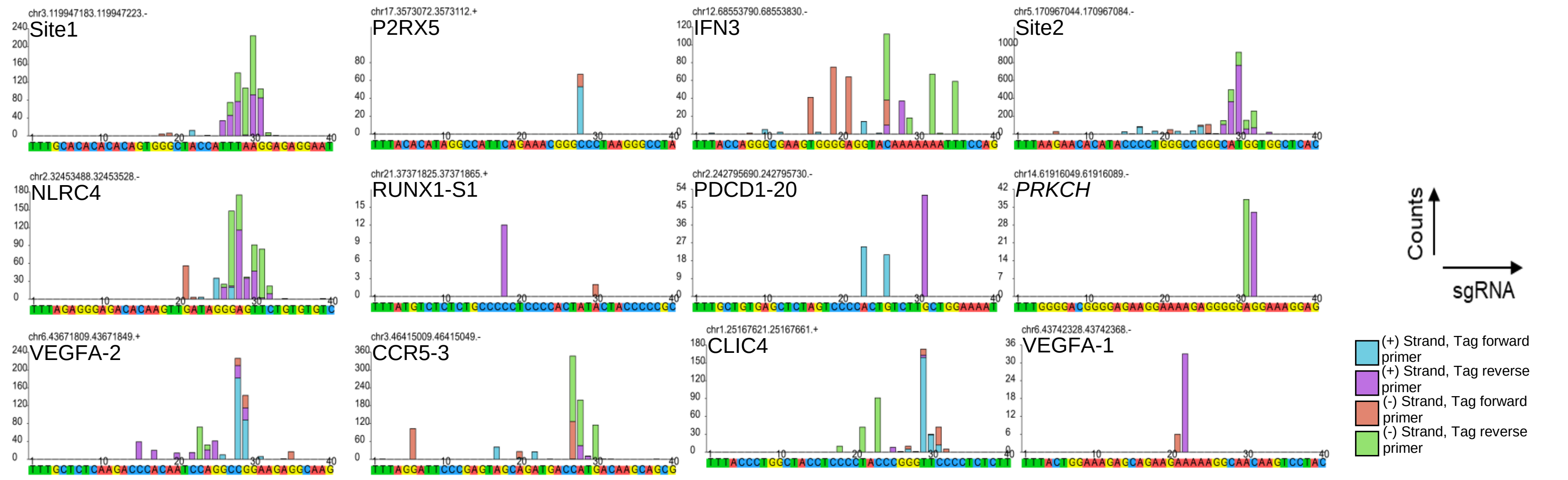

WT+ge3.0 (MCF7)

CRISPR-Un1Cas12f1 systems (MCF7 cell)

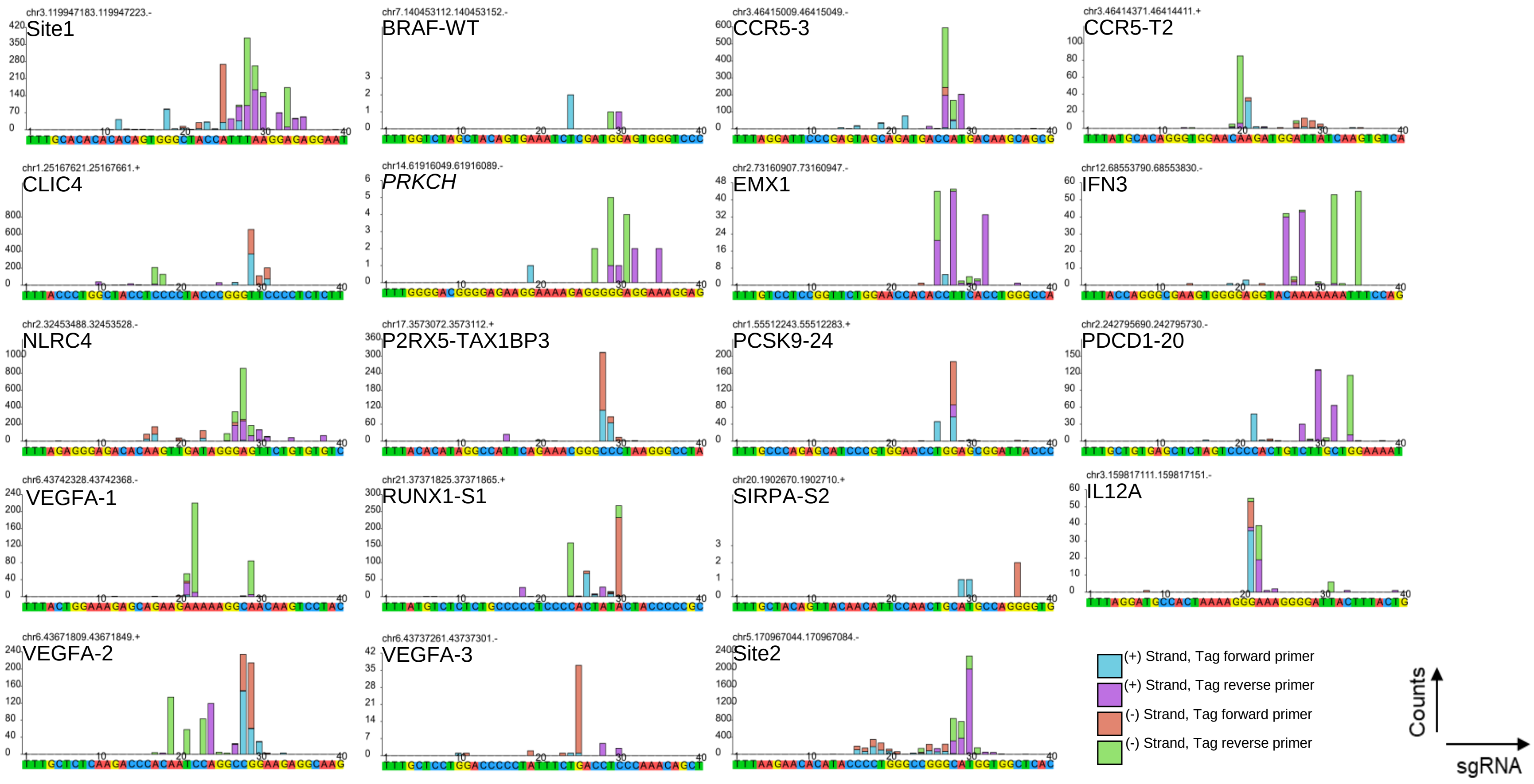

V3.1+ge3.0 (MCF7)

CRISPR-Un1Cas12f1 systems (MCF7 cell)

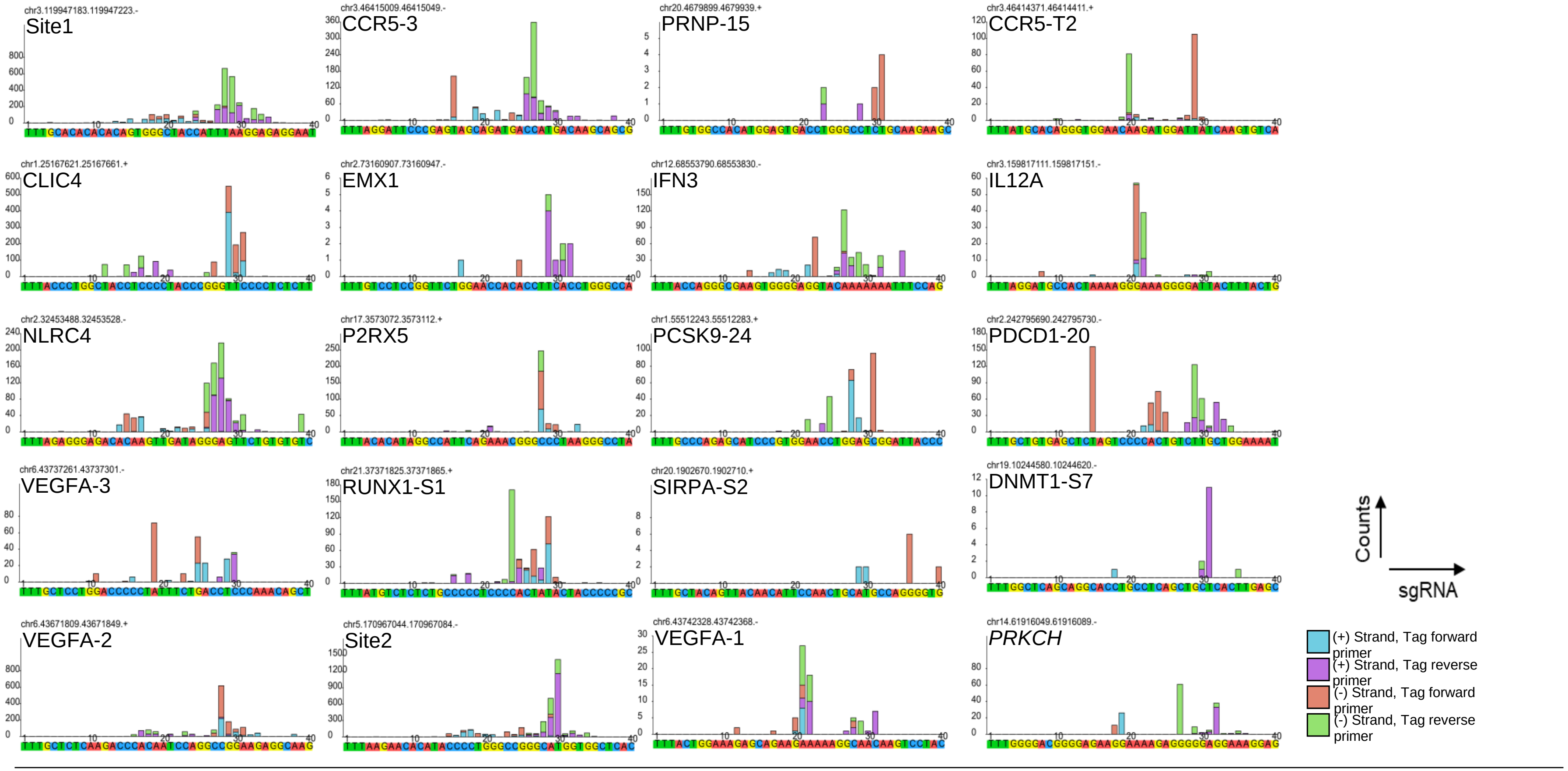

WT+ge4.0 (MCF7)

CRISPR-Un1Cas12f1 systems (MCF7 cell)

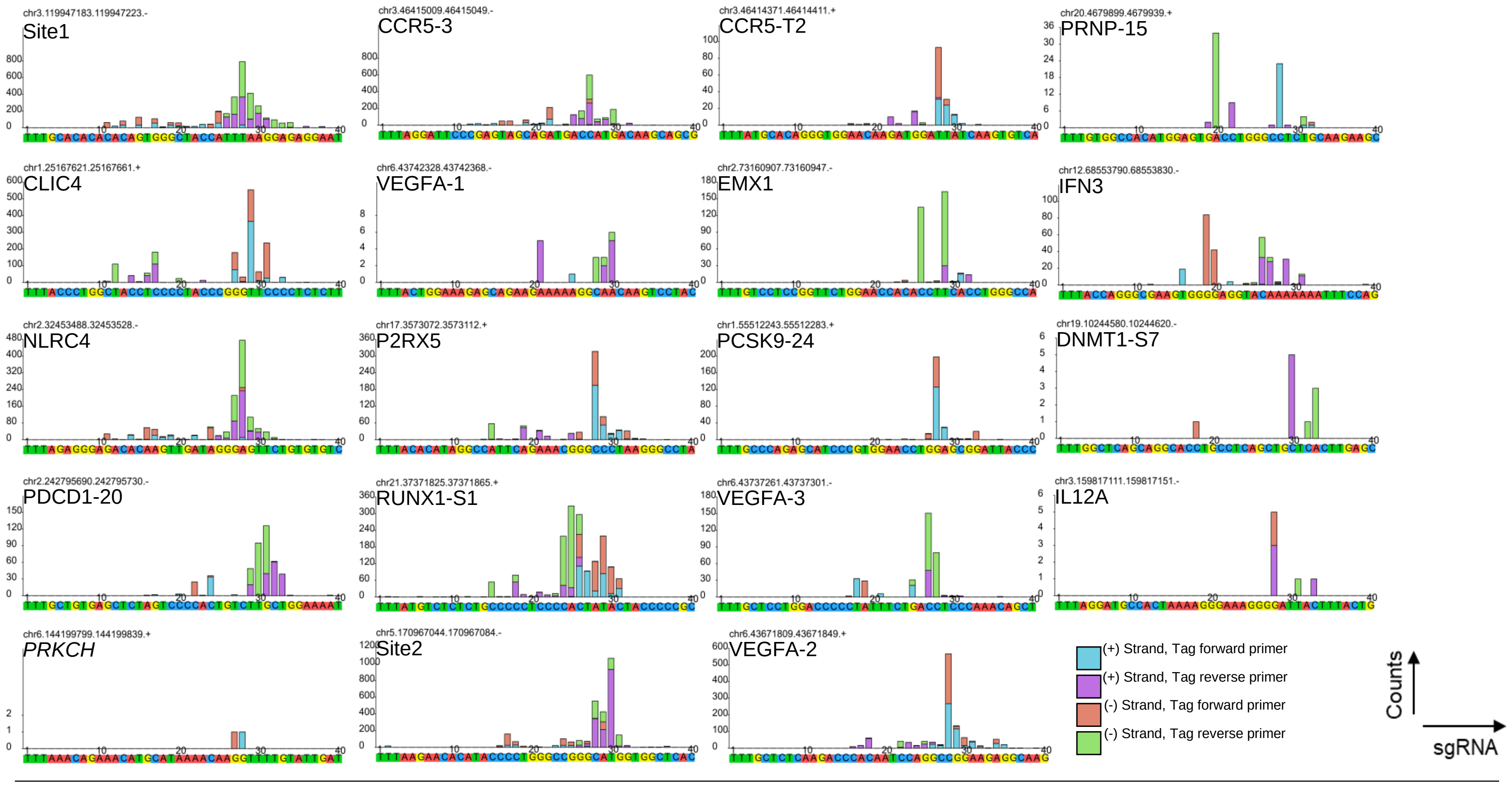

V3.1+ge4.0 (MCF7)

CRISPR-Un1Cas12f1 systems (MCF7 cell)

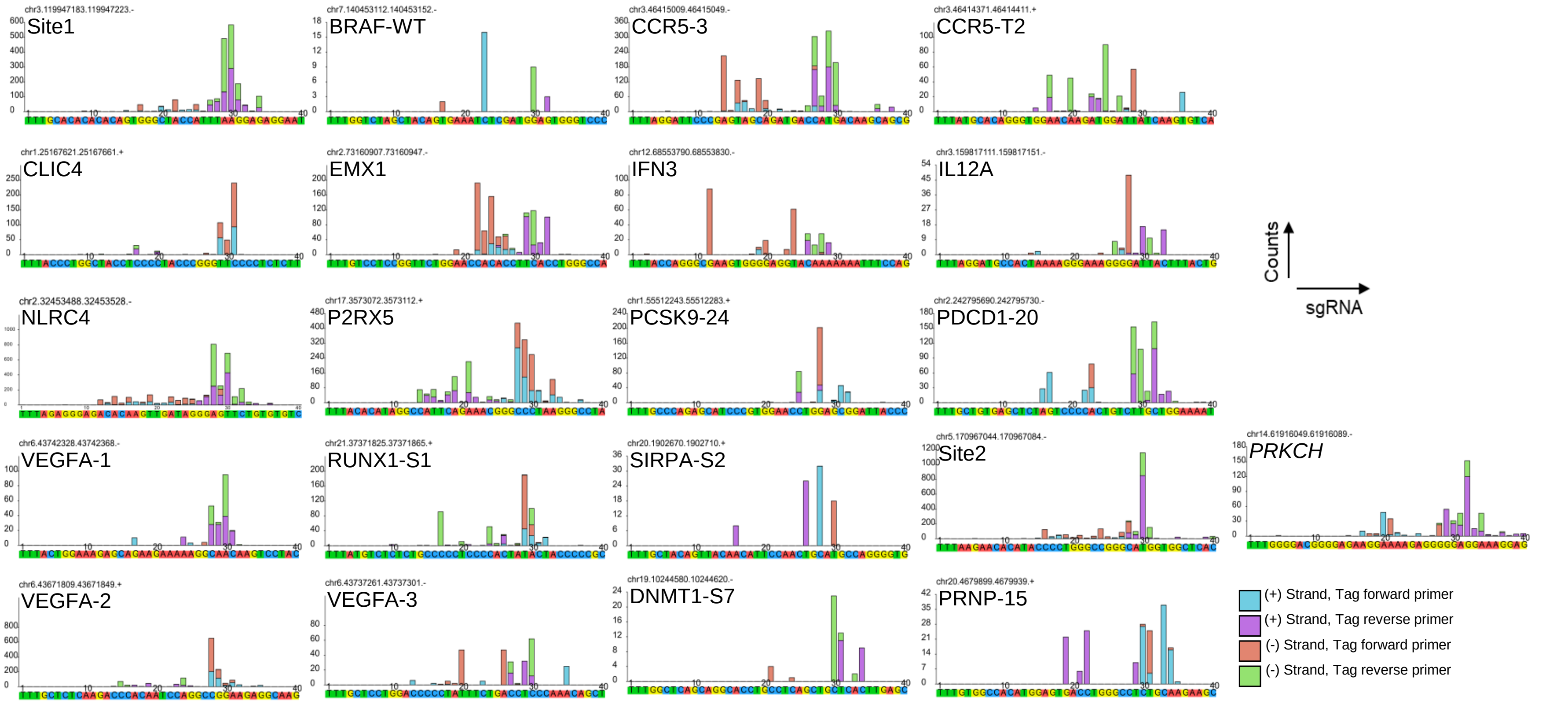

WT+ge4.1 (MCF7)

CRISPR-Un1Cas12f1 systems (MCF7 cell)

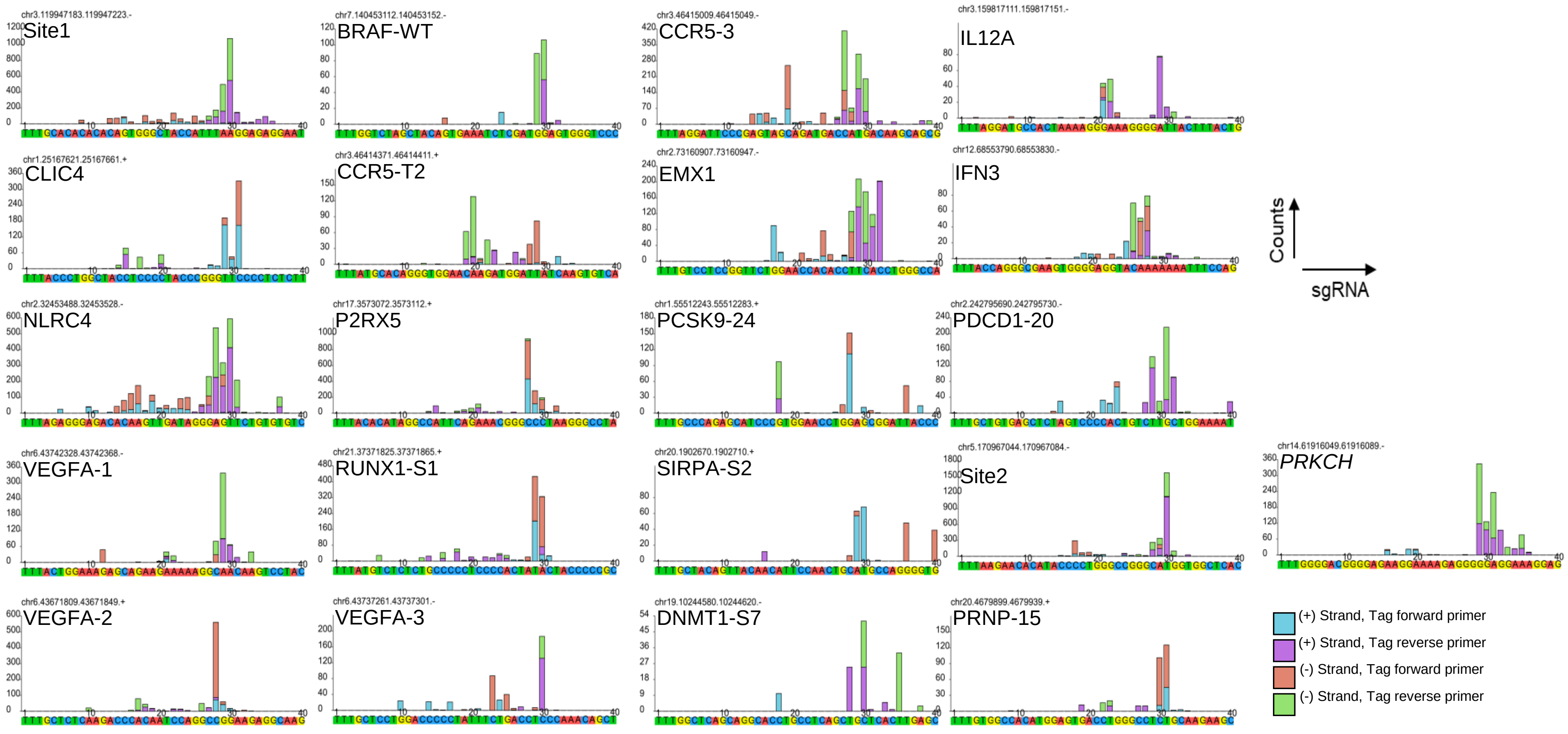

V3.1+ge4.1 (MCF7)

DNA-targeting editors (HEK293T cell)

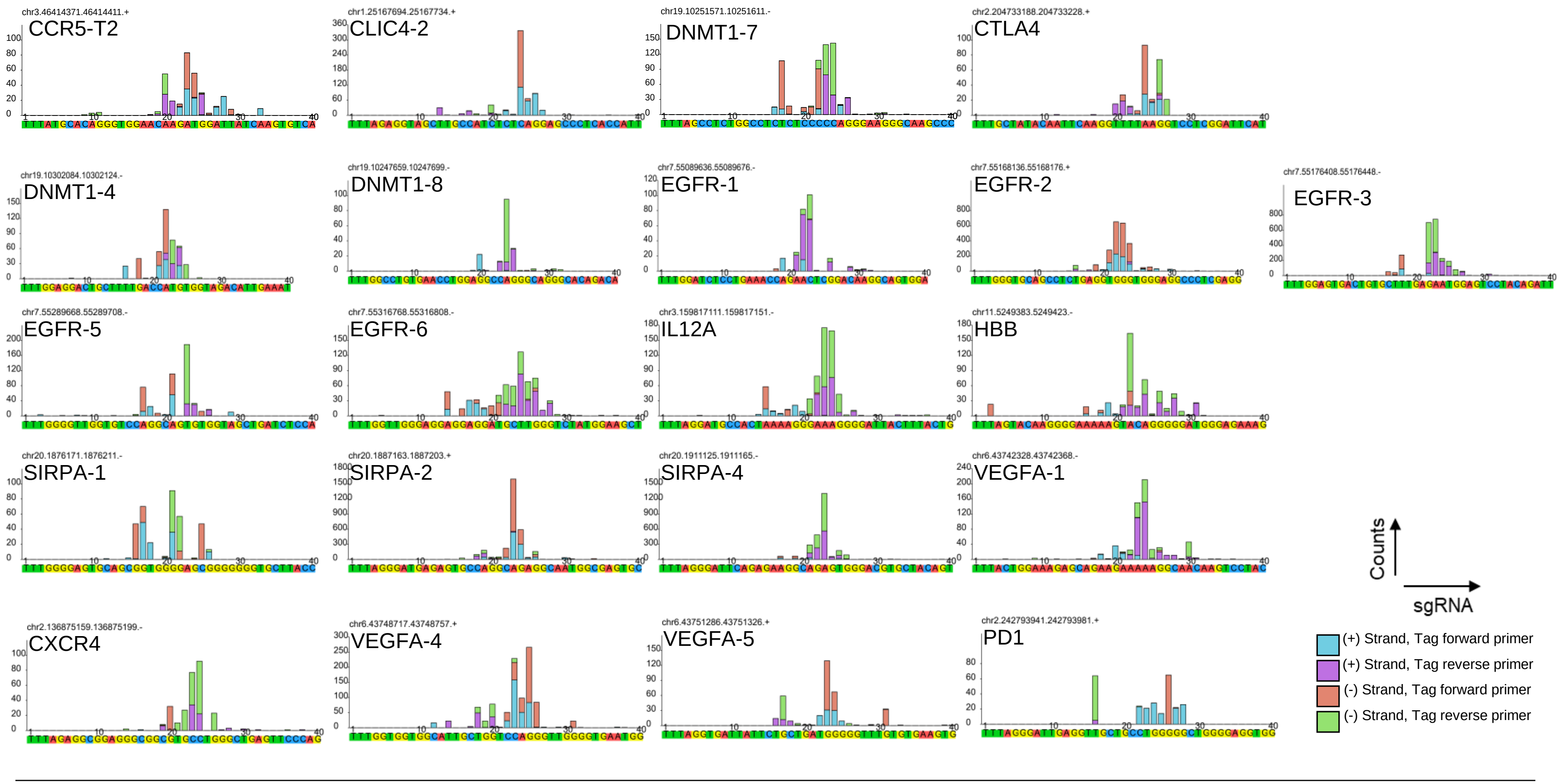

*As*Cas12a (HEK293T)

DNA-targeting editors (HEK293T cell)

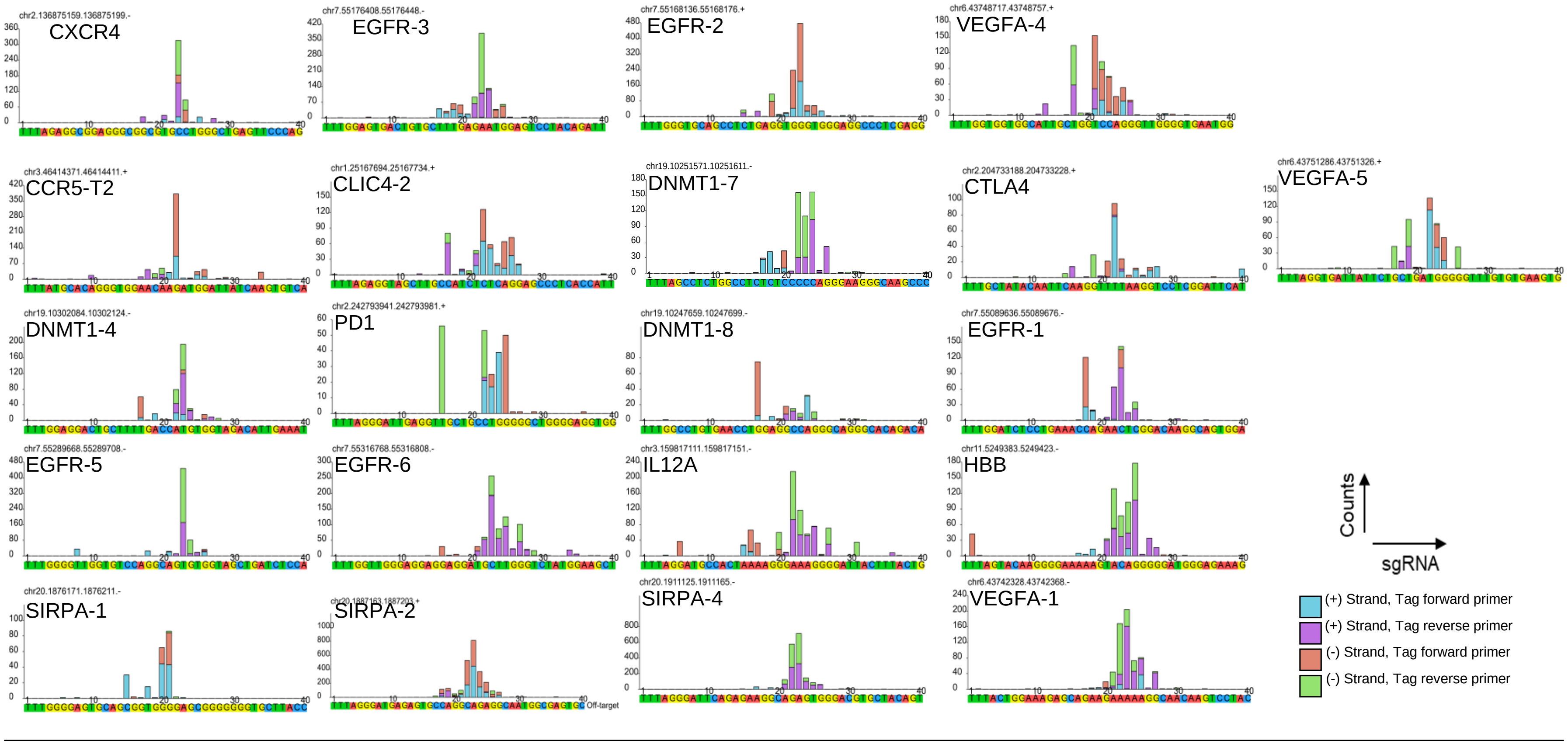

*Lb*Cas12a (HEK293T)

DNA-targeting editors (HEK293T cell)

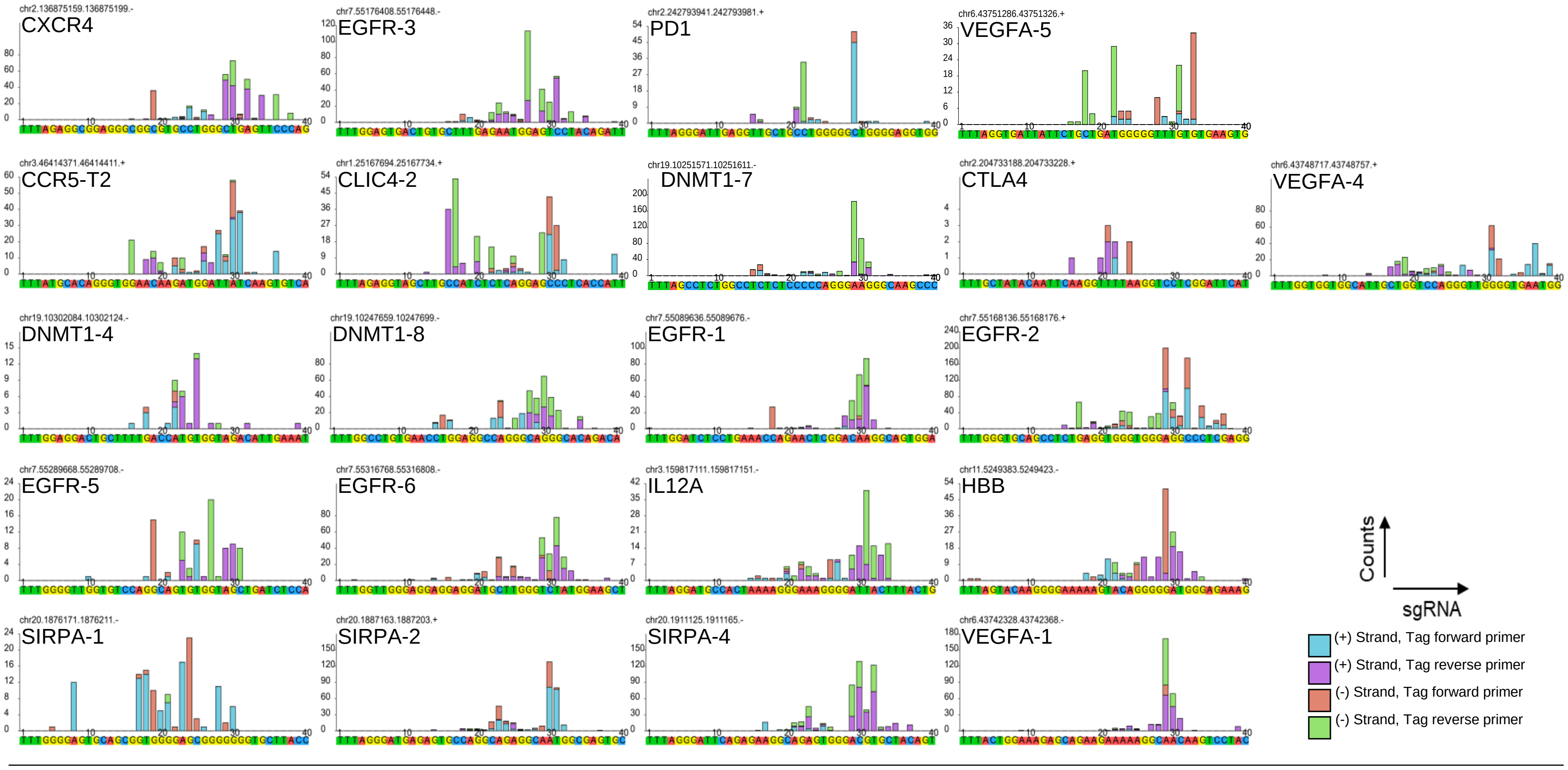

V3.1+ge4.1 (HEK293T)

DNA-targeting editors (HEK293T cell)

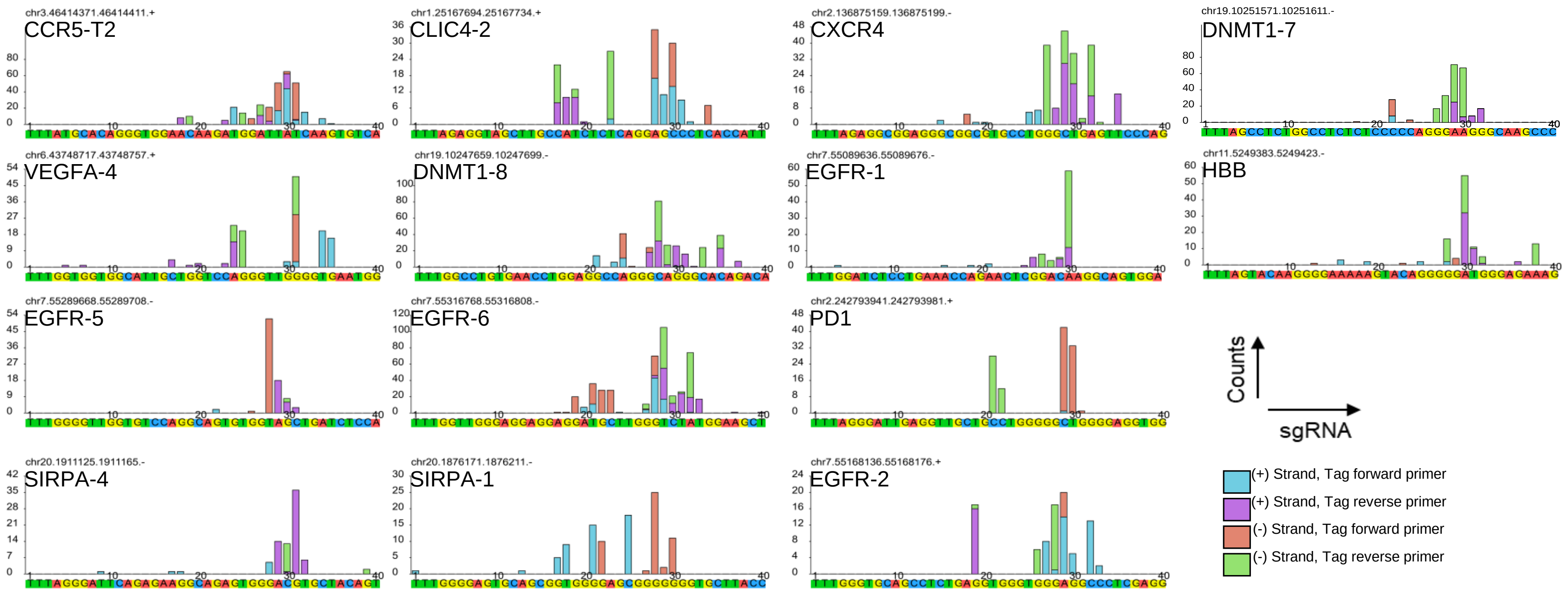

V3.1+ge4.0 (HEK293T)

DNA-targeting editors (HEK293T cell)

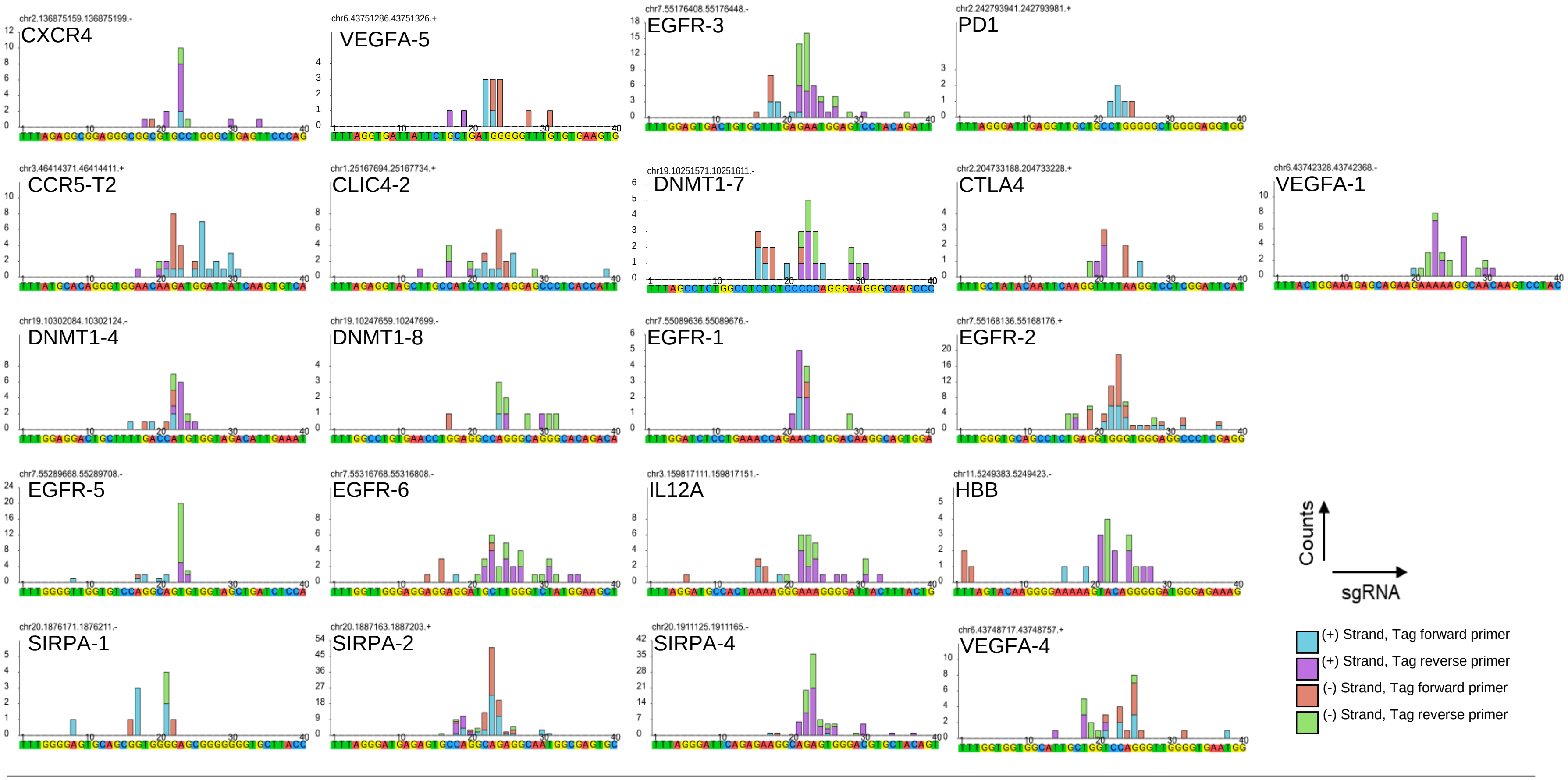

*As*Cas12f1 (HEK293T)

DNA-targeting editors (HEK293T cell)

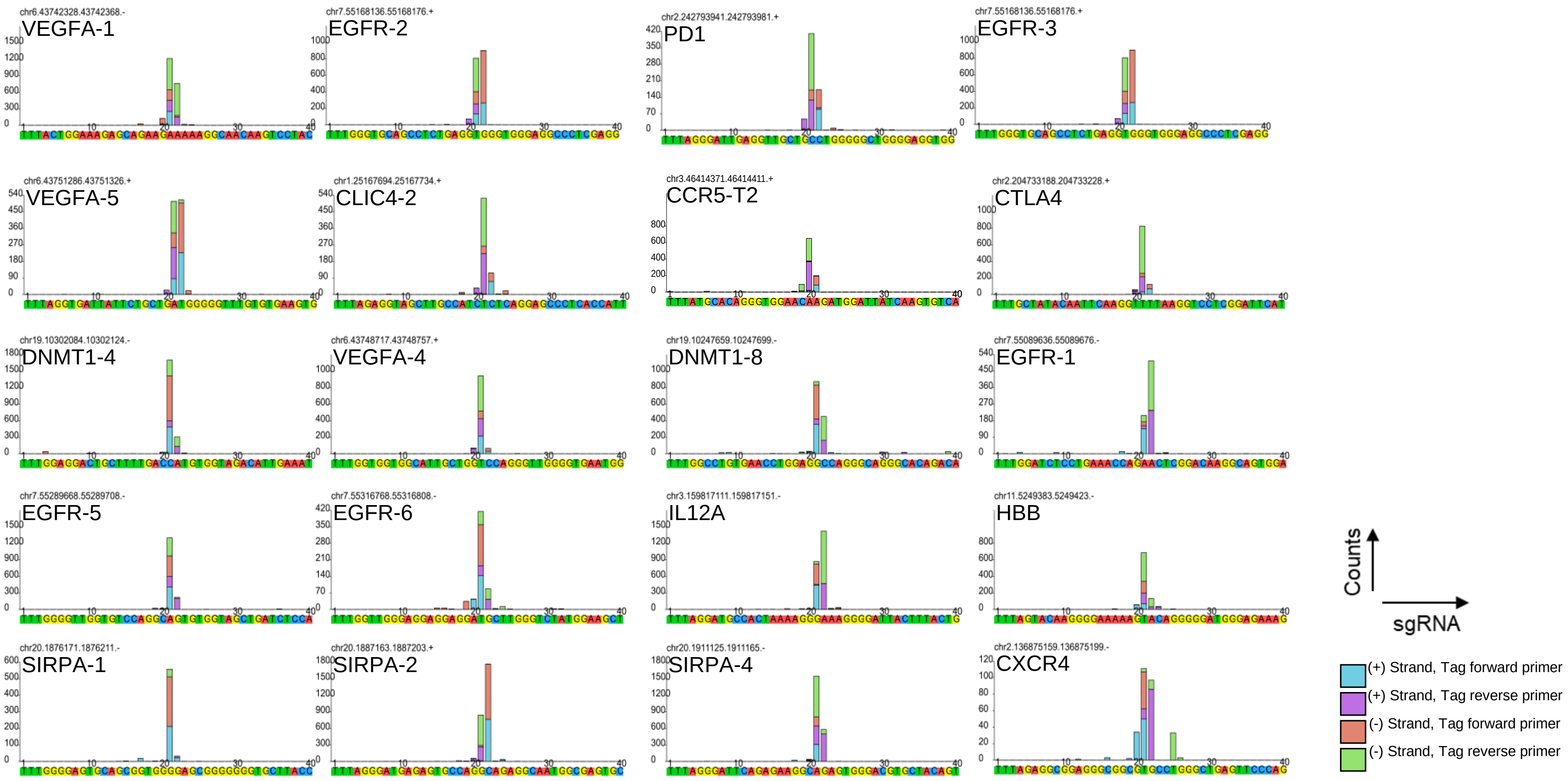

*Sp*Cas9 (HEK293T)

DNA-targeting editors (MCF7 cell)

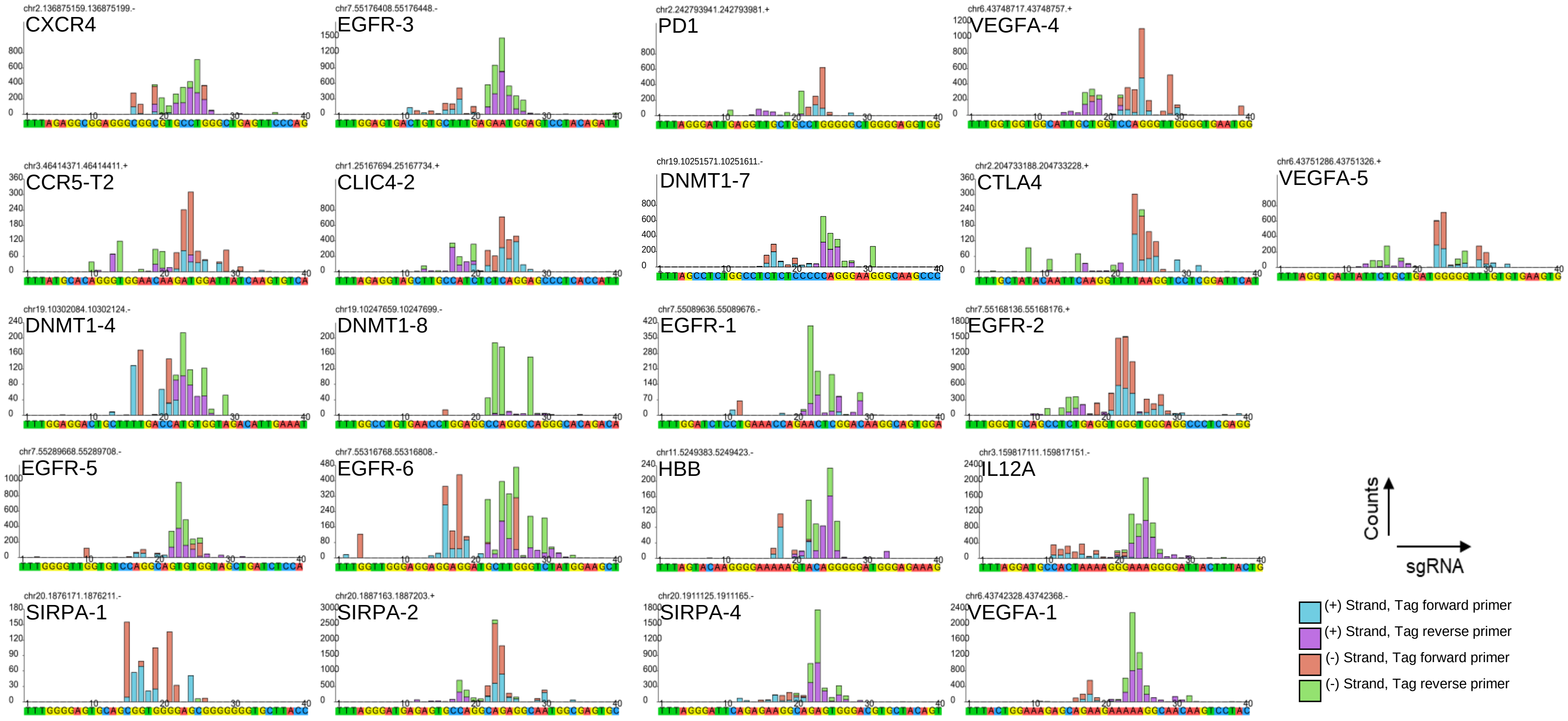

*As*Cas12a (MCF7)

DNA-targeting editors (MCF7 cell)

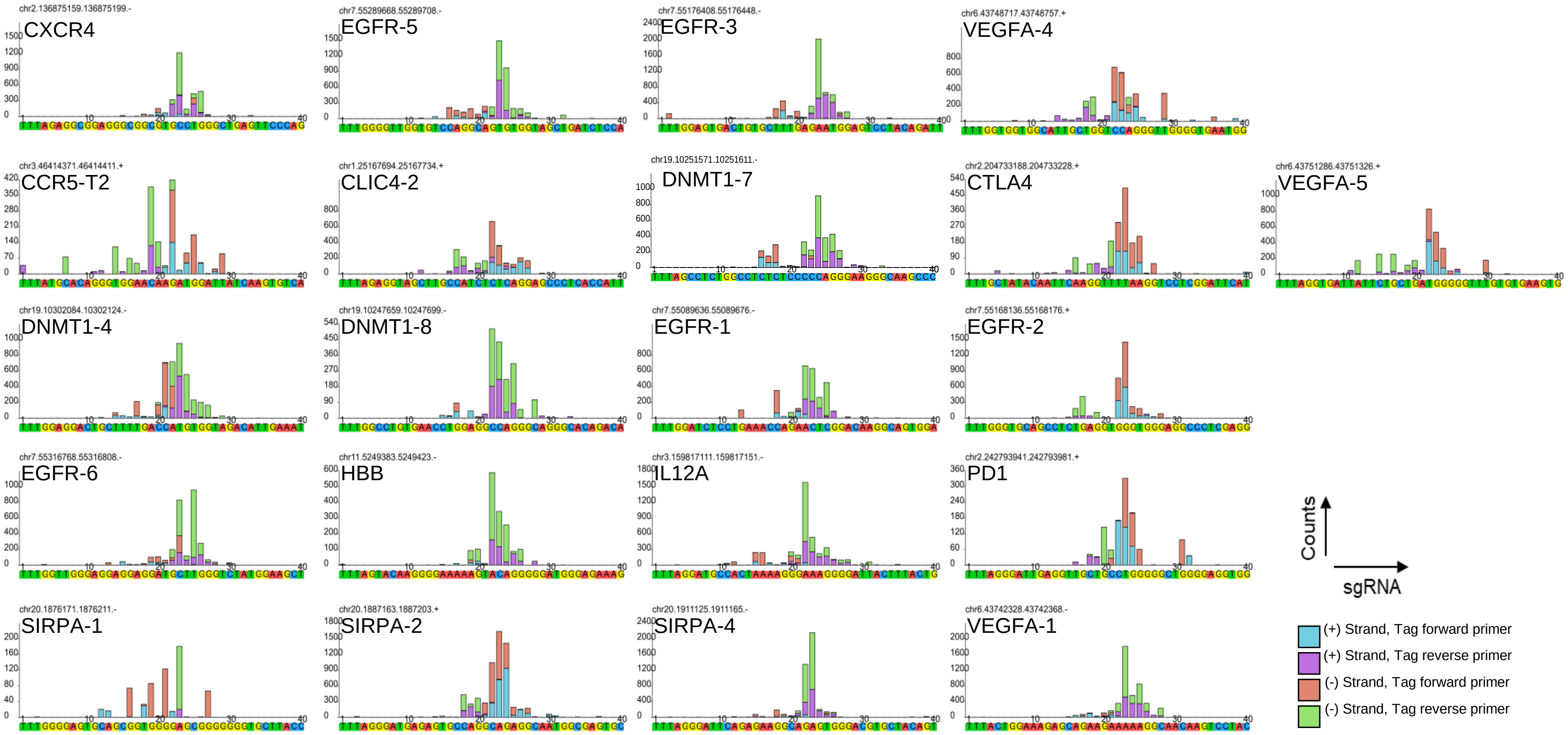

*Lb*Cas12a (MCF7)

DNA-targeting editors (MCF7 cell)

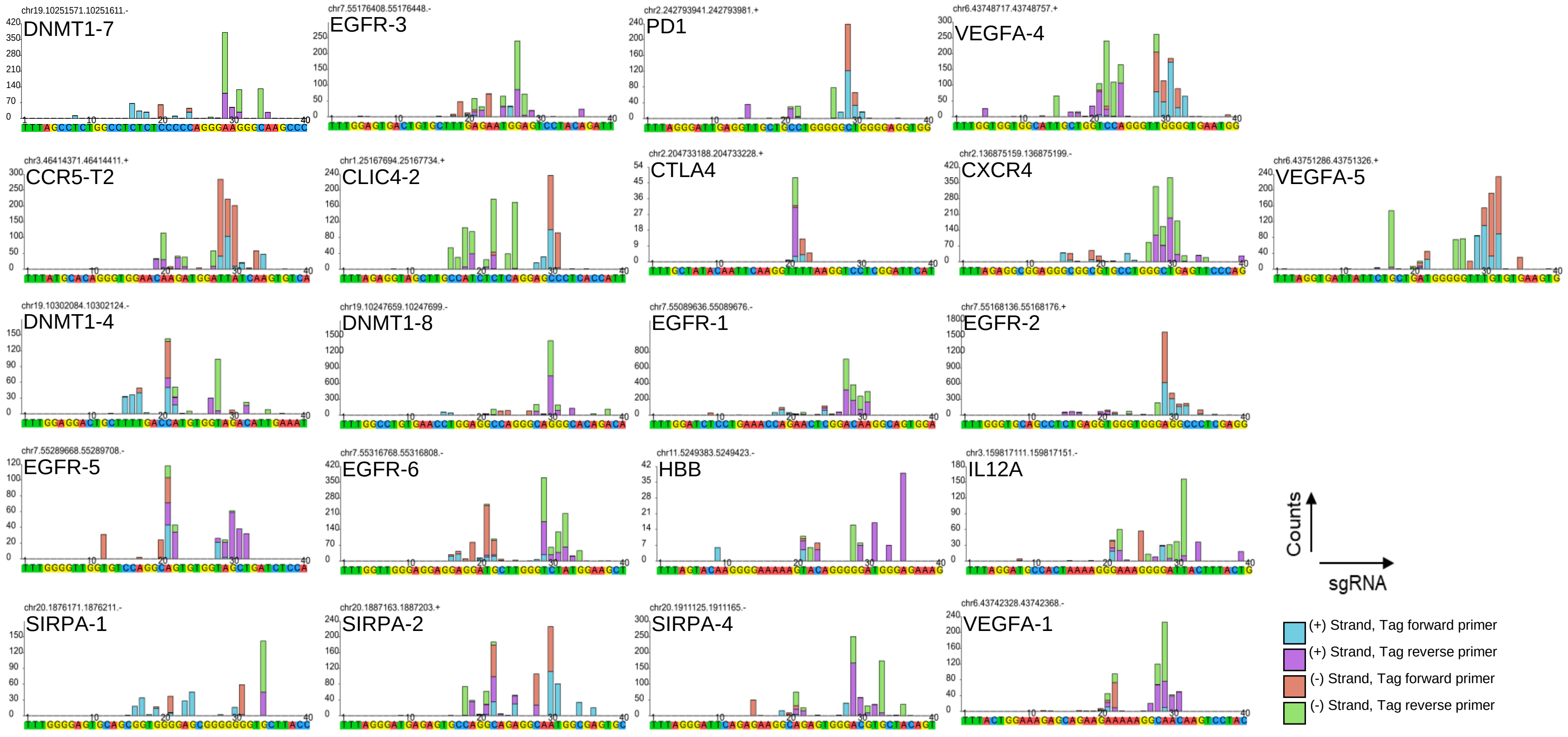

V3.1+ge4.1 (MCF7)

DNA-targeting editors (MCF7 cell)

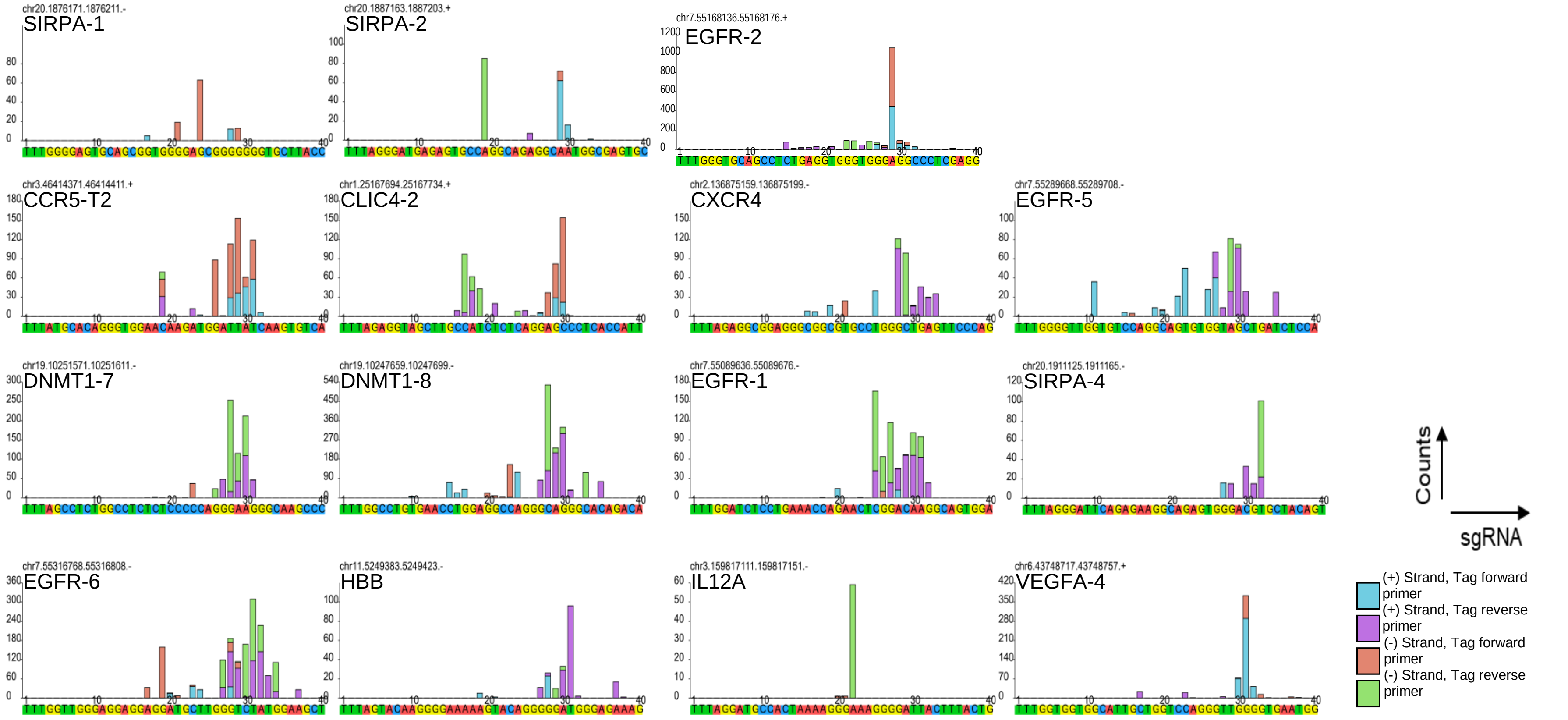

V3.1+ge4.0 (MCF7)

DNA-targeting editors (MCF7 cell)

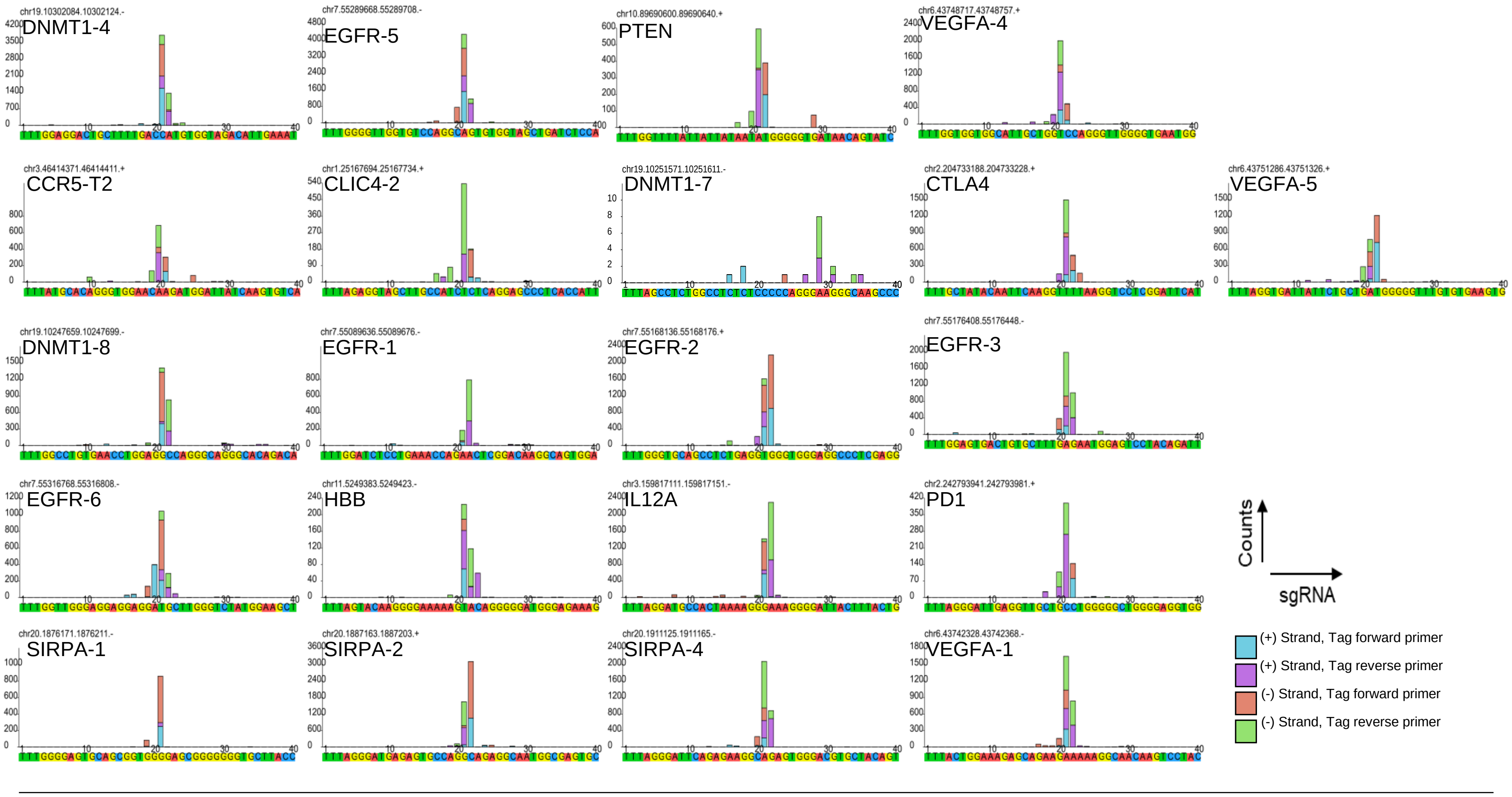

*Sp*Cas9 (MCF7)

DNA-targeting editors (K562 cell)

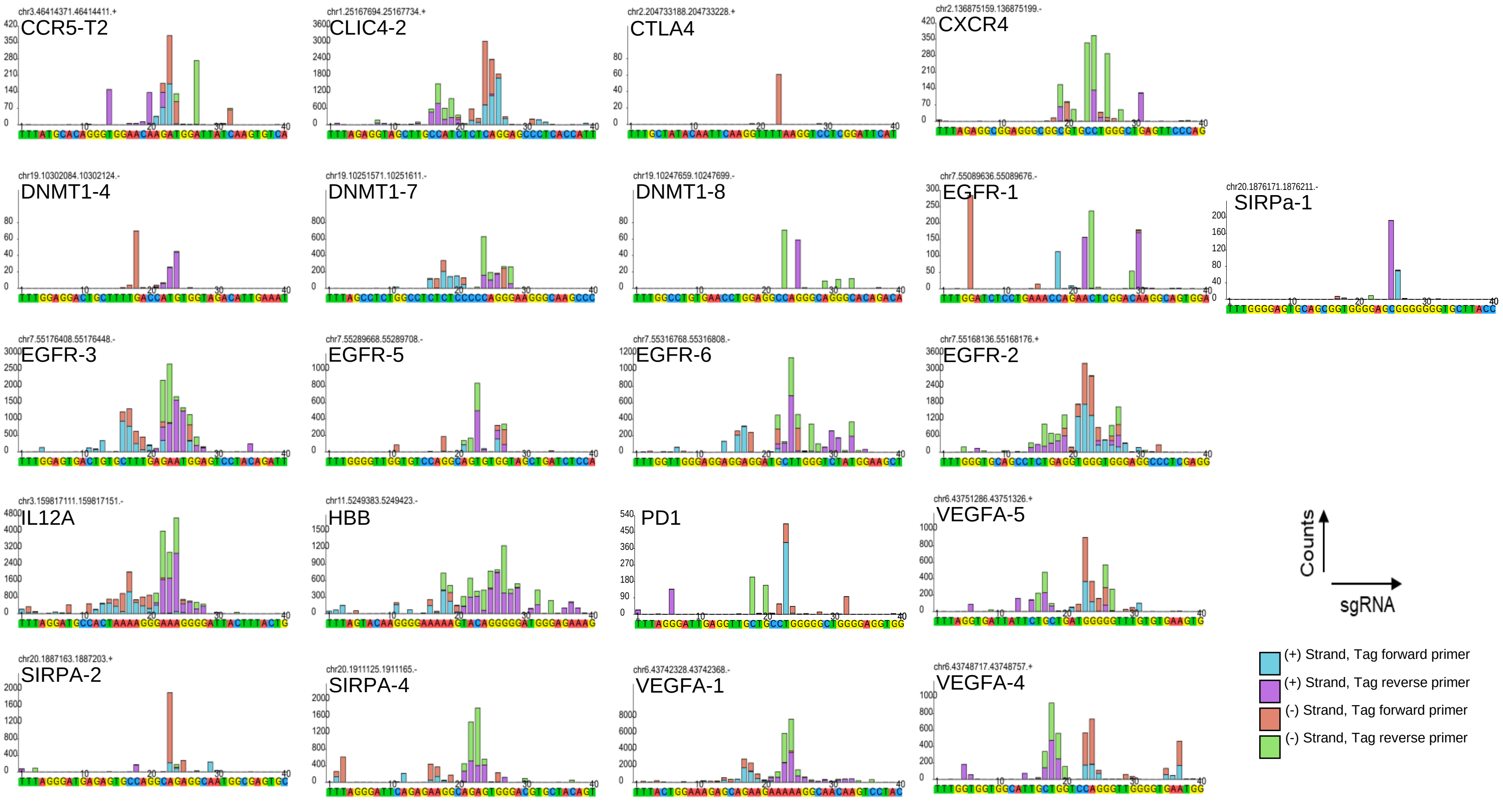

*As*Cas12a (K562 cells)

DNA-targeting editors (K562 cell)

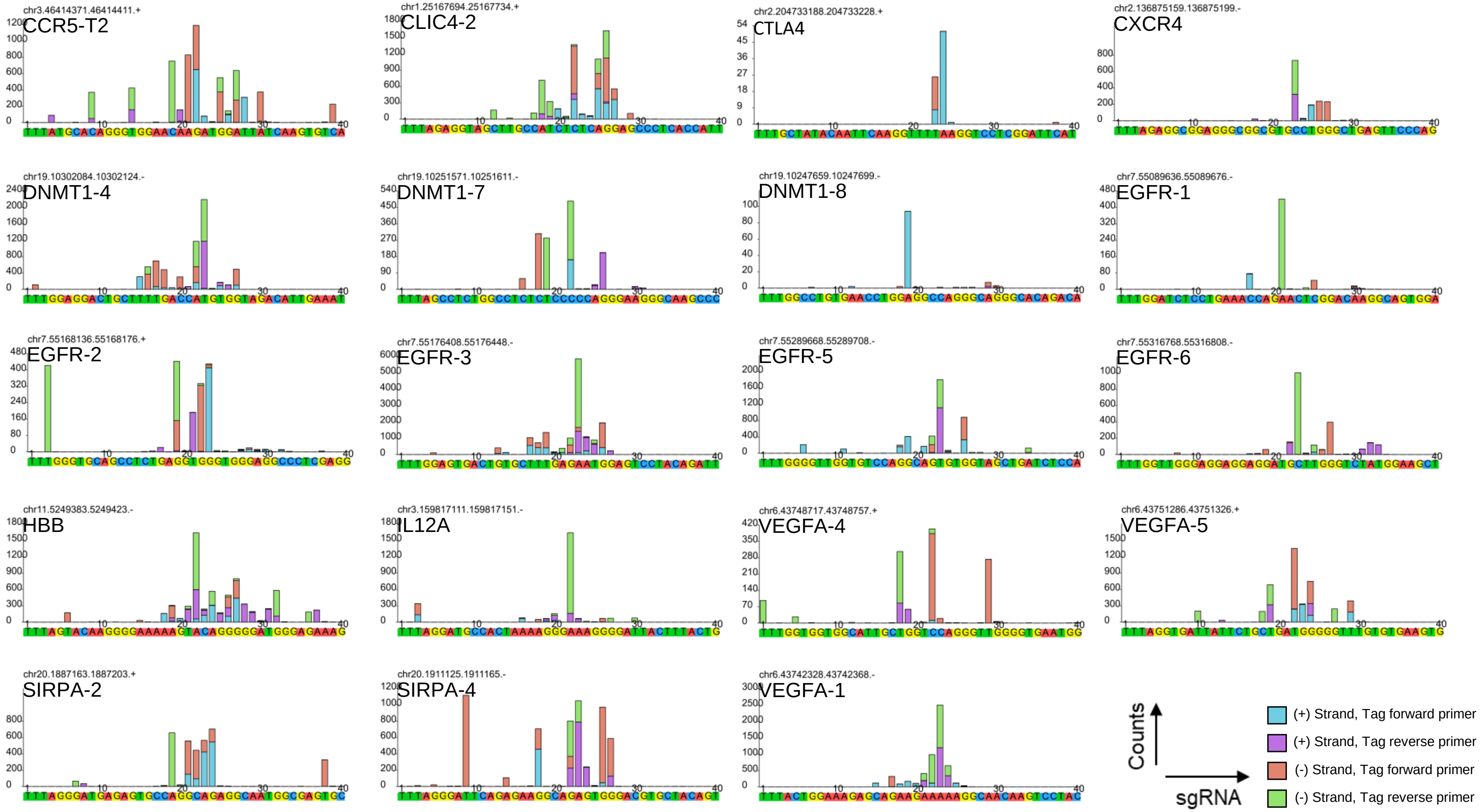

*Lb*Cas12a (K562 cells)

DNA-targeting editors (K562 cell)

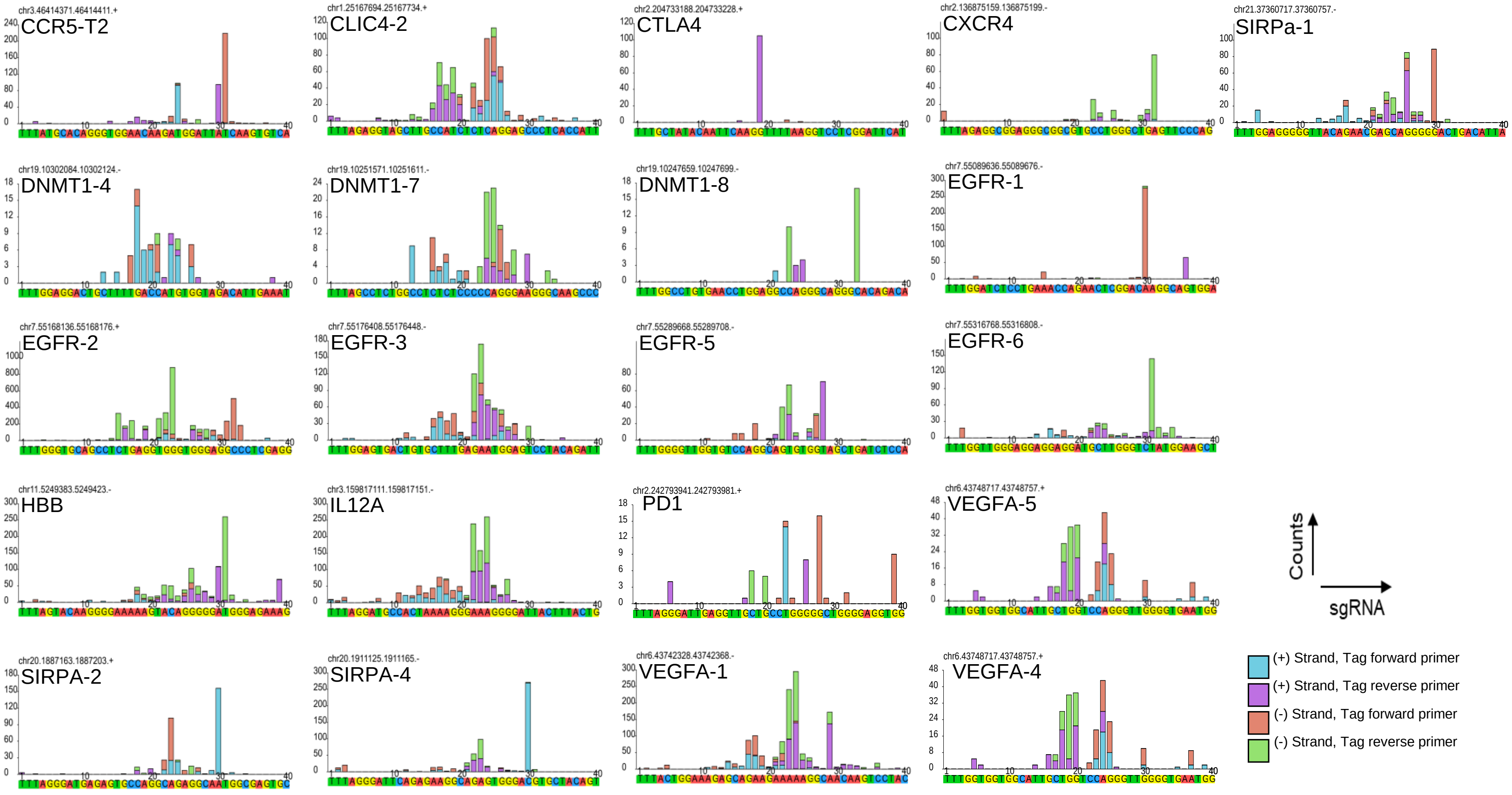

V3.1+ge4.1 (K562 cells)

DNA-targeting editors (K562 cell)

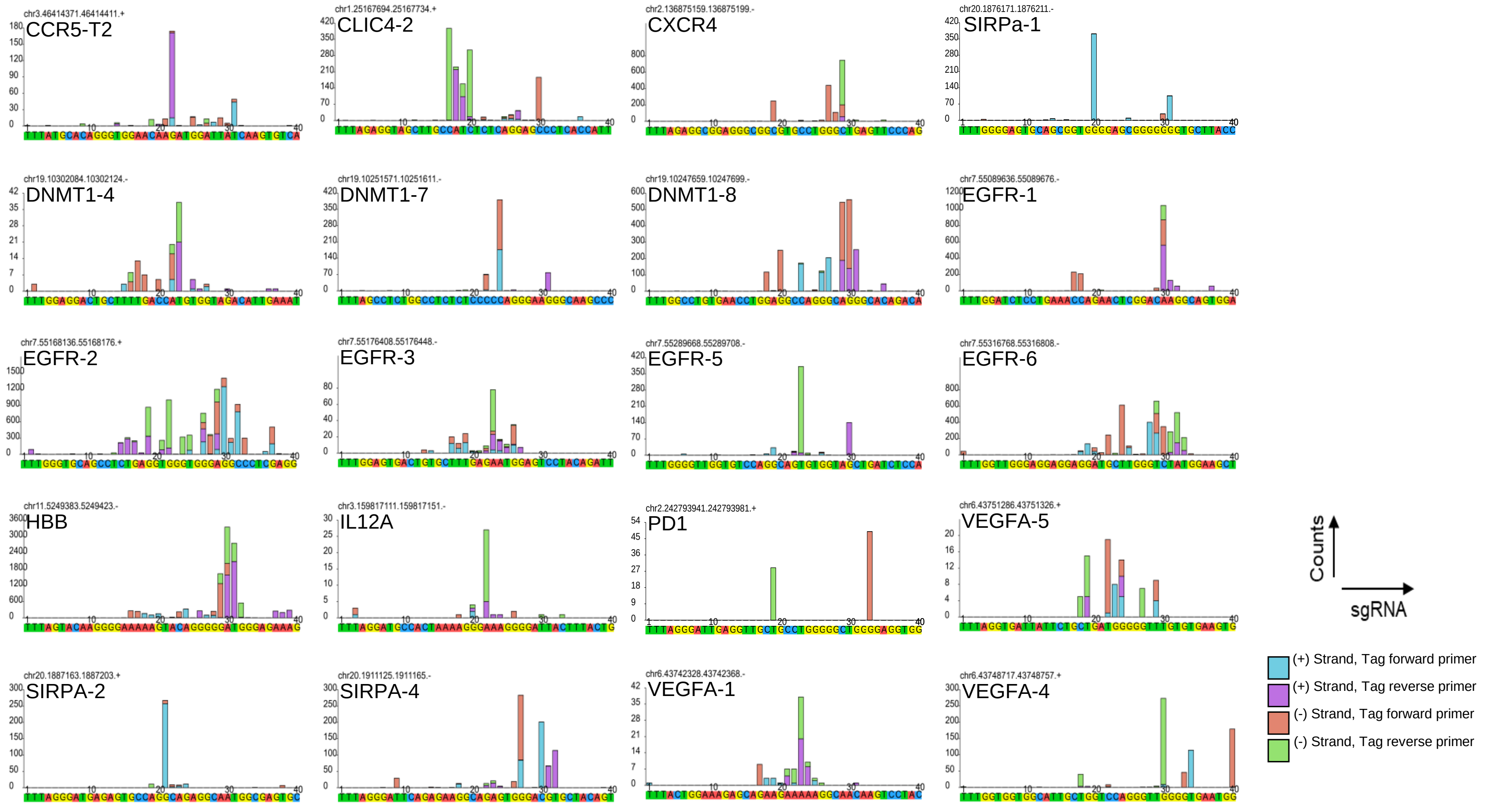

V3.1+ge4.0 (K562 cells)

DNA-targeting editors (K562 cell)

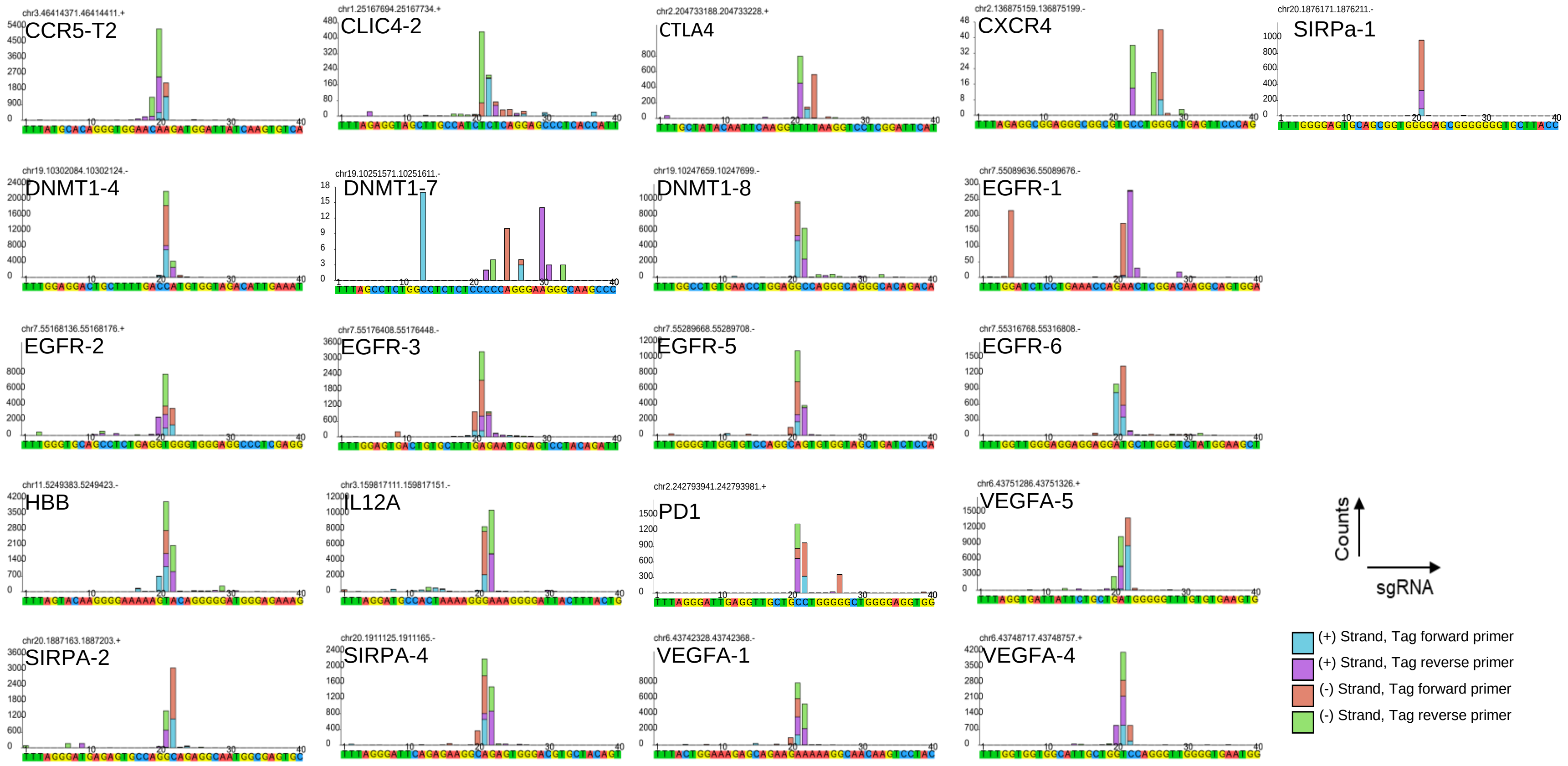

*Sp*Cas9 (K562 cells)

DNA-targeting editors (Jurkat cell)

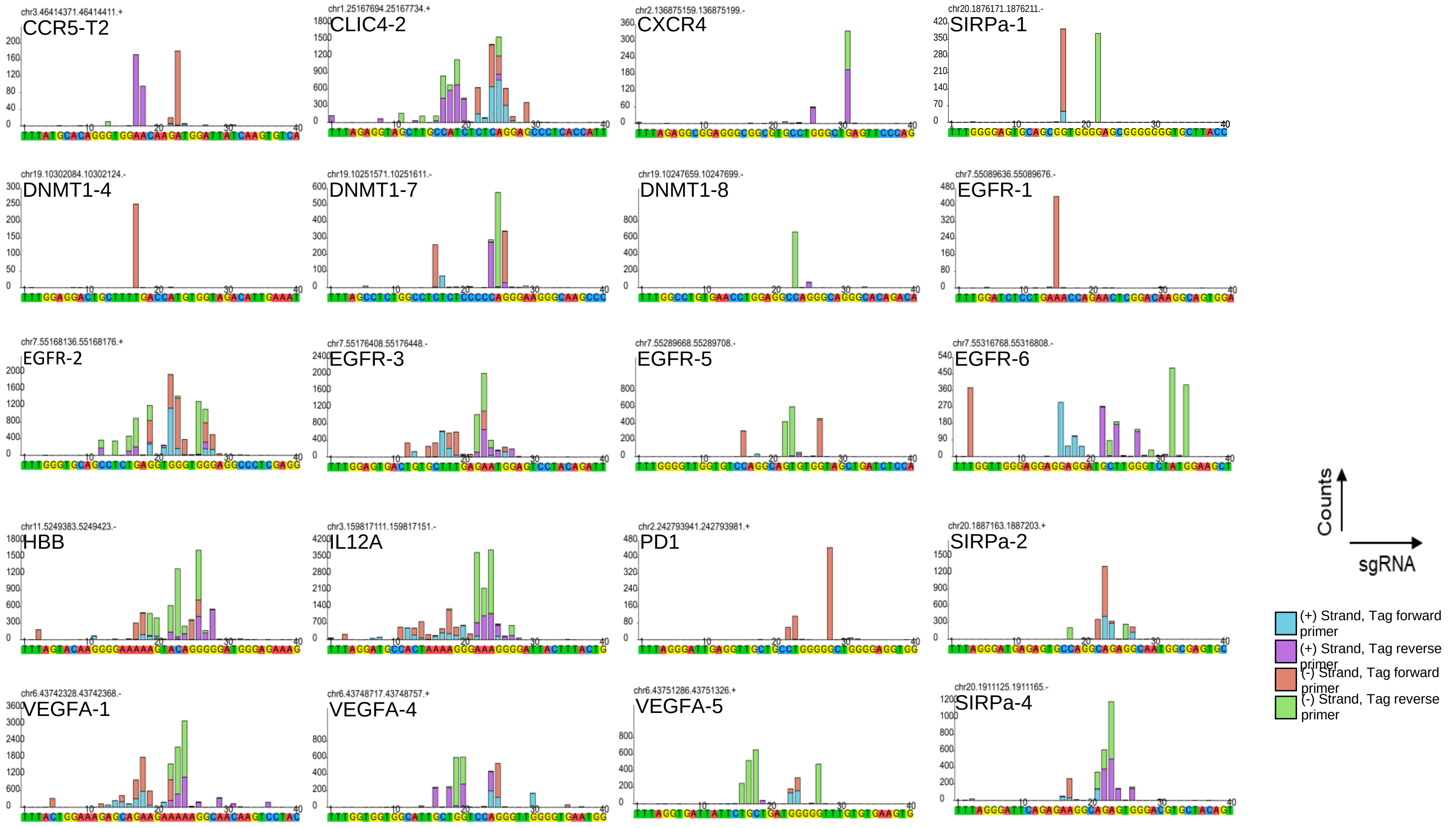

*As*Cas12a (Jurkat cells)

DNA-targeting editors (Jurkat cell)

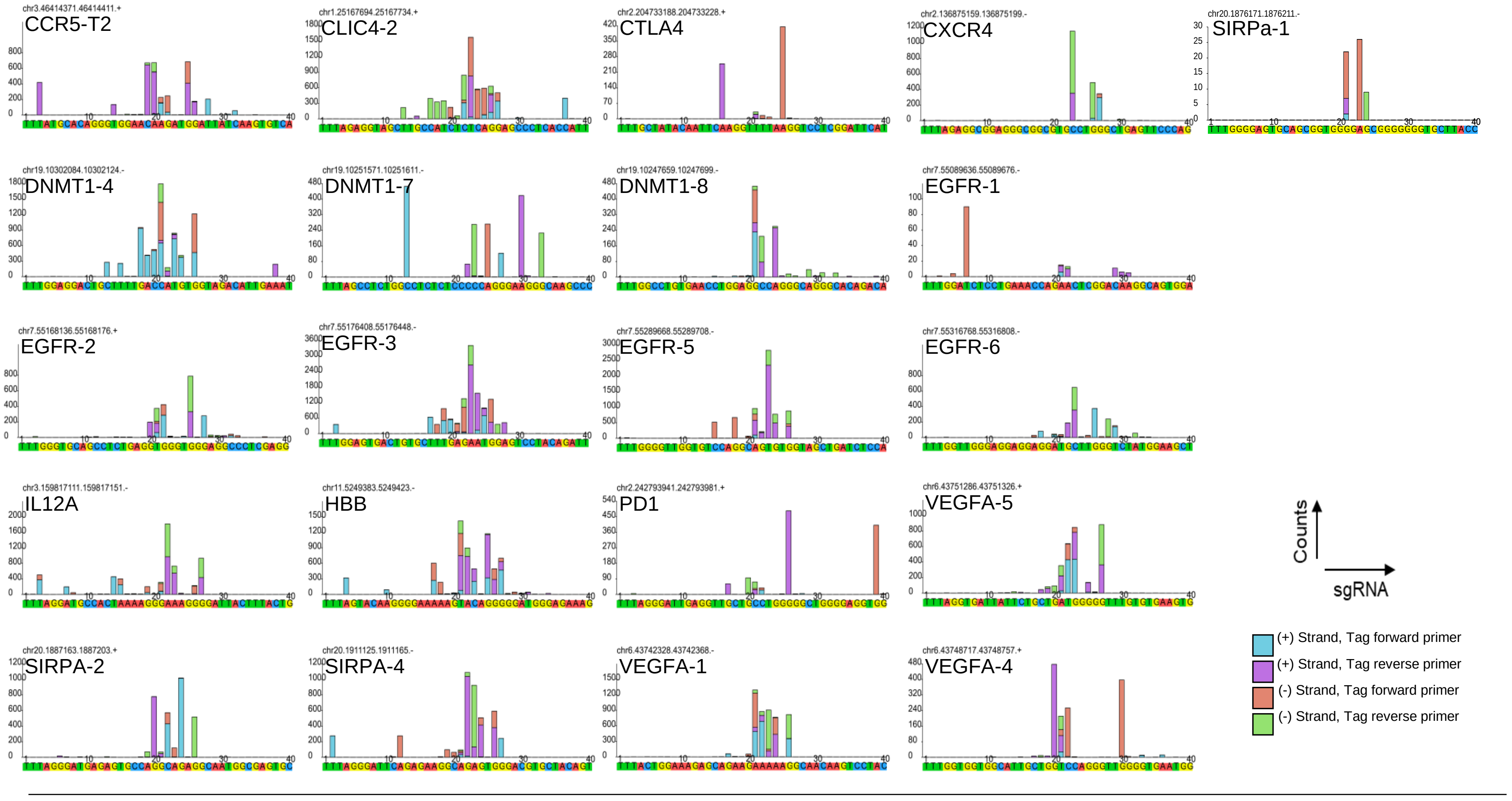

*Lb*Cas12a (Jurkat cells)

DNA-targeting editors (Jurkat cell)

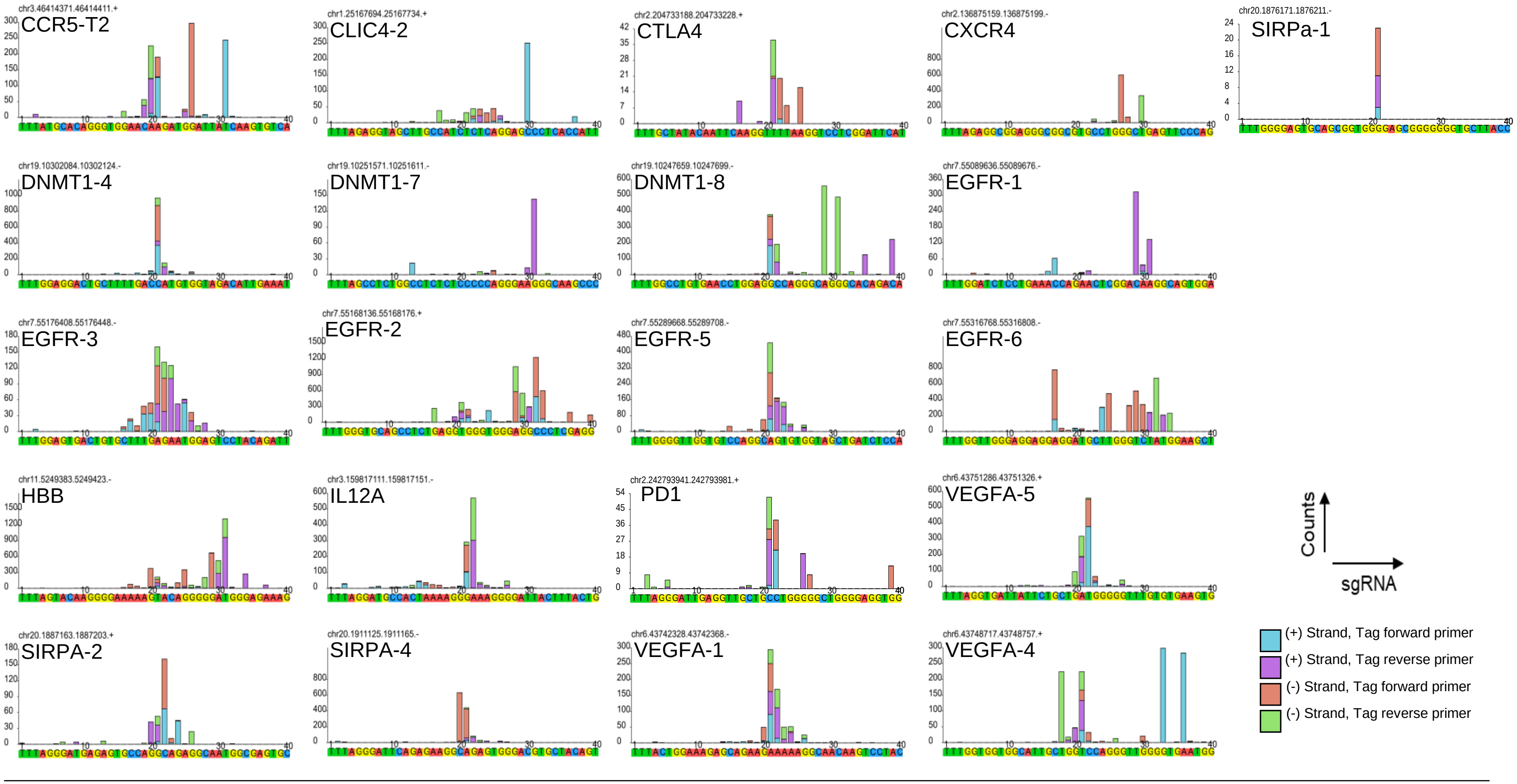

V3.1+ge4.1 (Jurkat cells)

DNA-targeting editors (Jurkat cell)

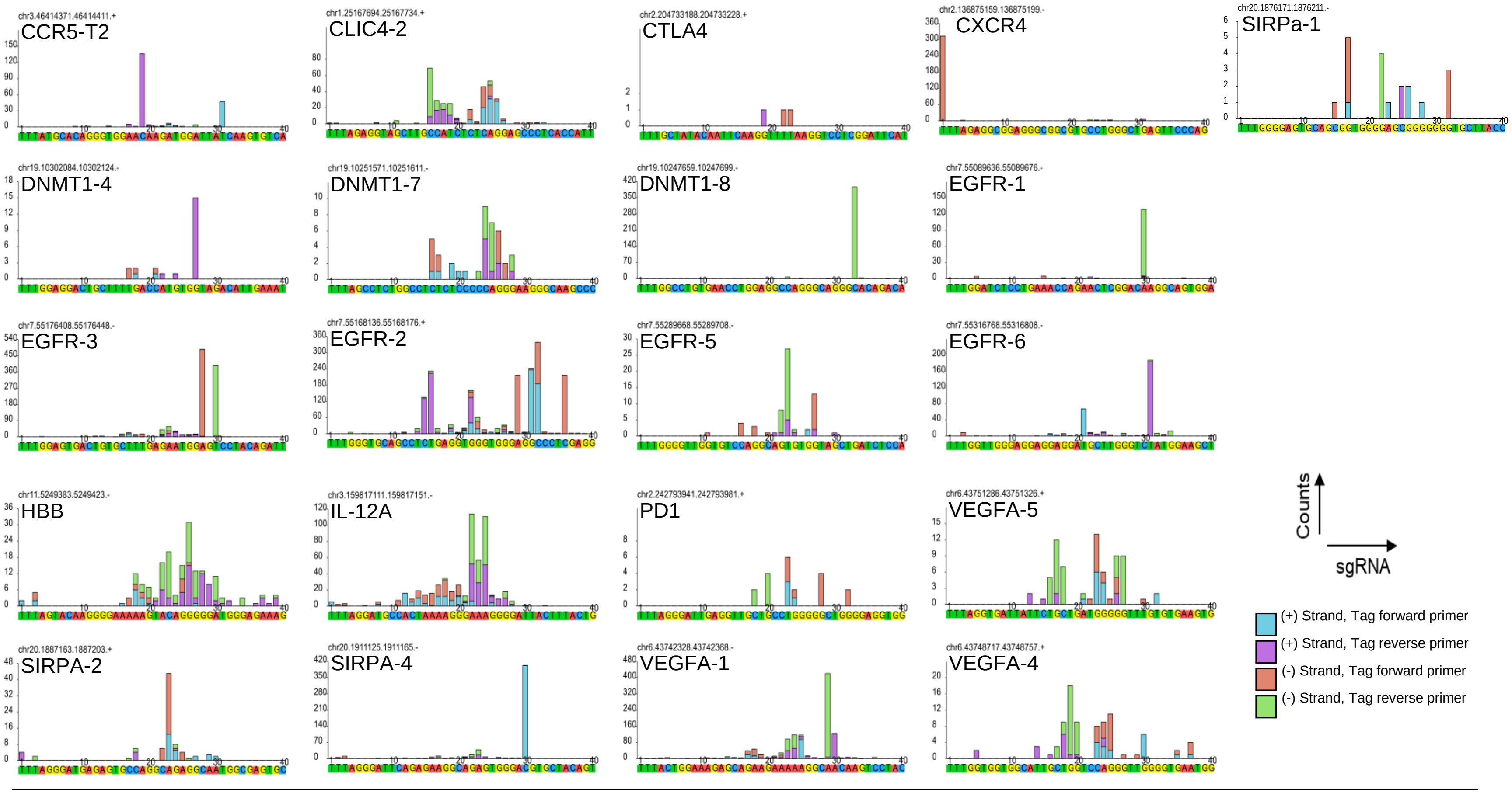

V3.1+ge4.0 (Jurkat cells)

DNA-targeting editors (Jurkat cell)

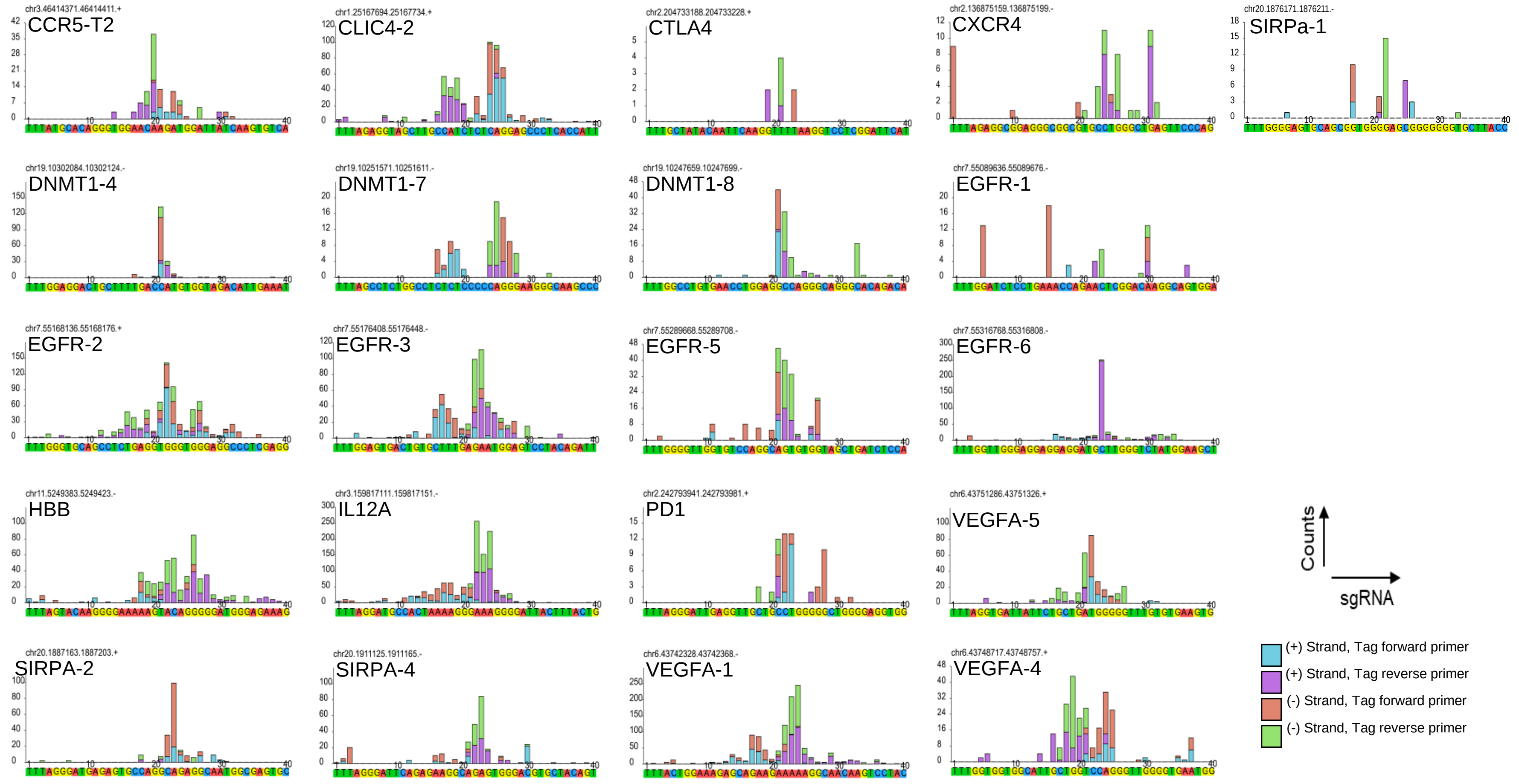

*As*Cas12f1 (Jurkat cells)

DNA-targeting editors (Jurkat cell)

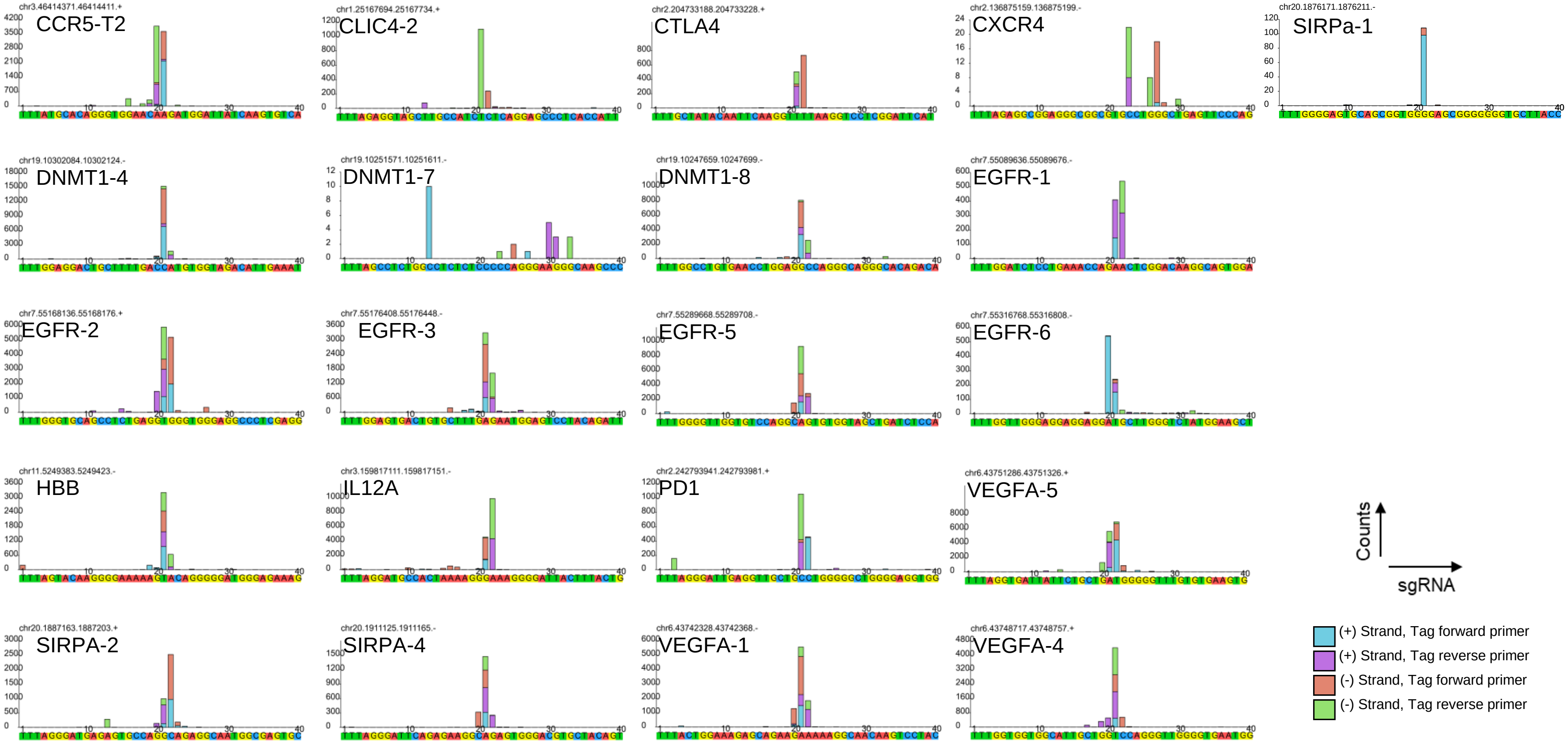

*Sp*Cas9 (Jurkat cells)

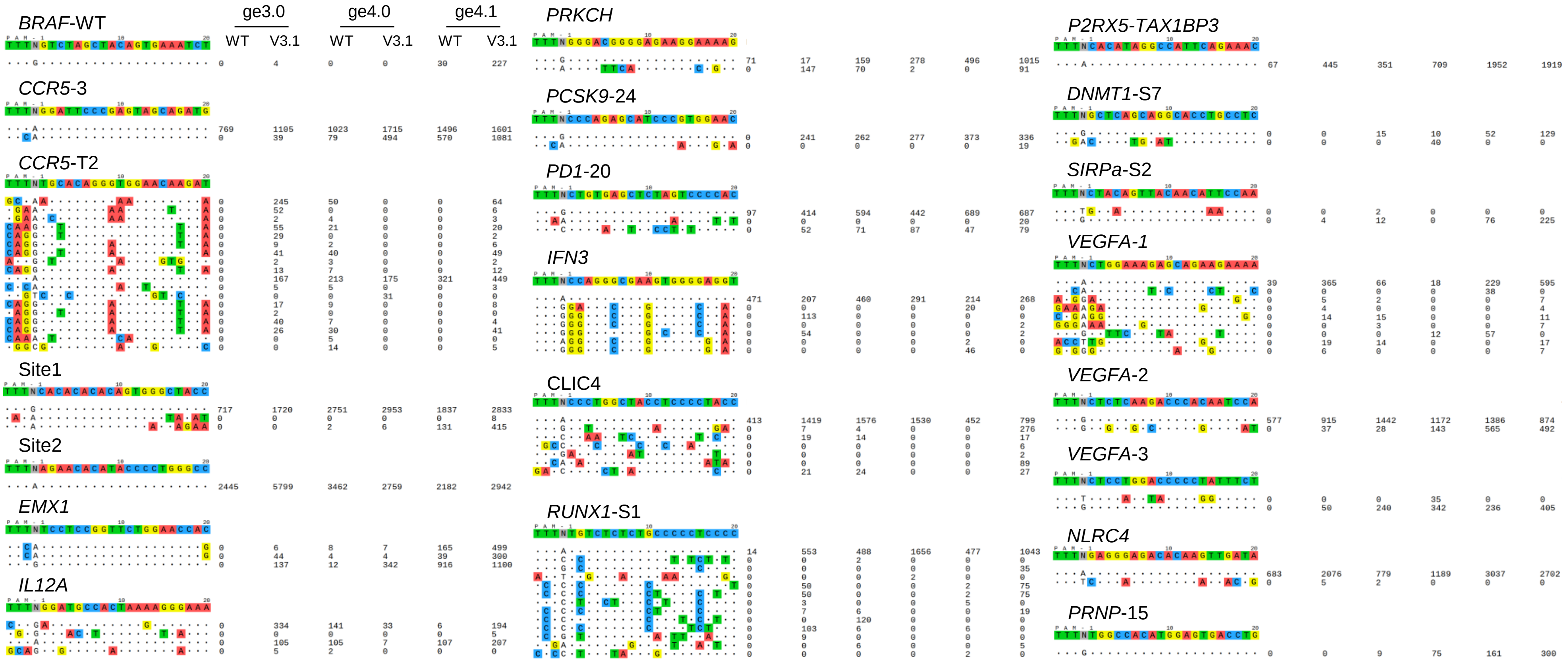

Fig. S6 Specificity comparison of the engineered CRISPR-Un1Cas12f1 systems by Tag-seq in MCF7 cells

Supplementary Figure 6. Specificity comparison of the engineered CRISPR-Un1Cas12f1 system by Tag-seq in MCF7 cells. MCF7 cells were transfected by PEI method with the plasmids expressing Un1Cas12f1-WT or -V3.1 and a pooled twenty-one sgRNAs (containing the ge3.0, ge4.0, or ge4.1), and the Tag-oligo DNA sequence. Genomic DNA was harvested three days post-transfection for libraries construction and Tag-seq analysis. Read counts represented a measure of cleavage frequency at a given site, mismatched positions within the spacer or PAM are highlighted in color.

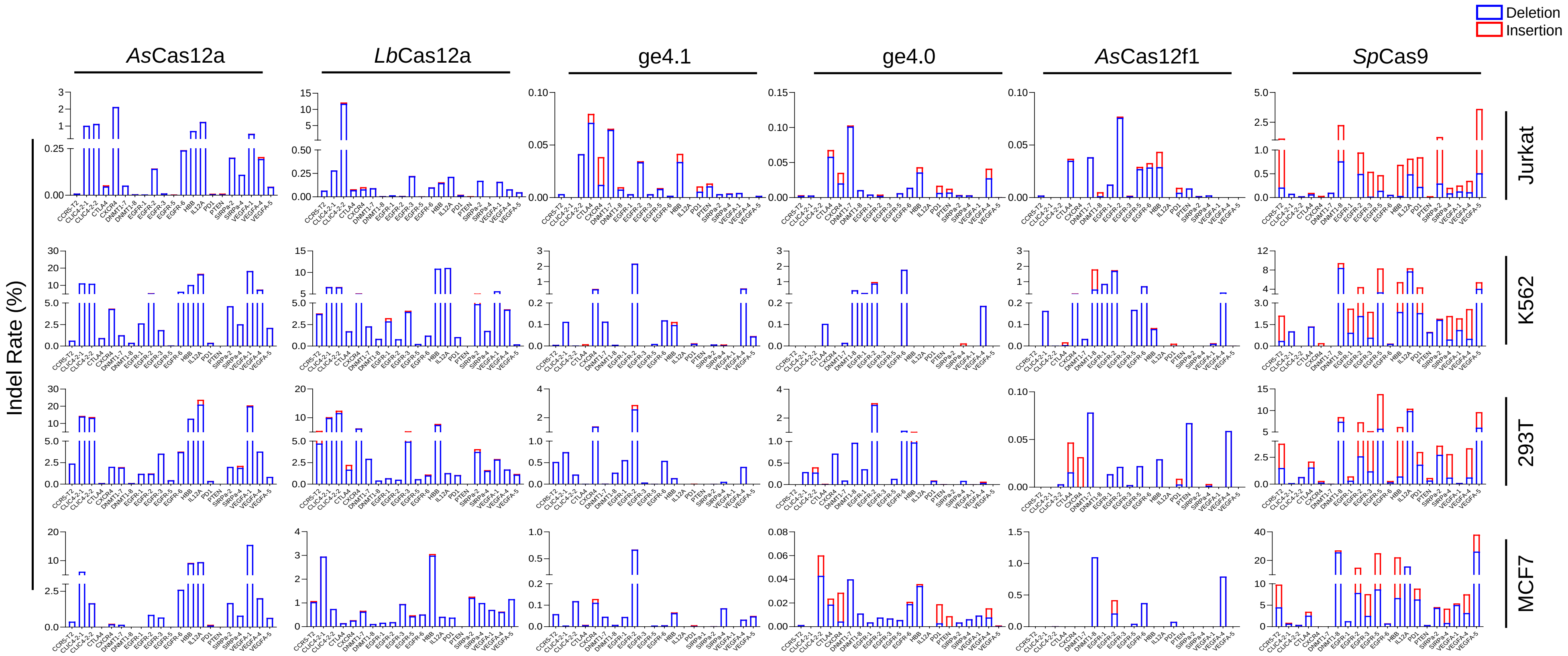

Figure S7 Activity comparison of the DNA editing CRISPR system by Deep-seq in 4 cell lines

Supplementary Figure 7. Activity comparison of the DNA editing CRISPR system by Deep-seq in 4 cell lines. Deep-seq revealed the editing activities of the DNA CRISPR editors by targeting twenty-one sites in various cell lines, including HEK293T, MCF7, K562, and Jurkat. Cells were transfected with the plasmids expressing DNA-editing nucleases, the corresponding sgRNAs that pooled with twenty-one guides. Two days post-transfection genomic DNA was harvested for Deep-seq libraries construction and editing efficiency analyses.

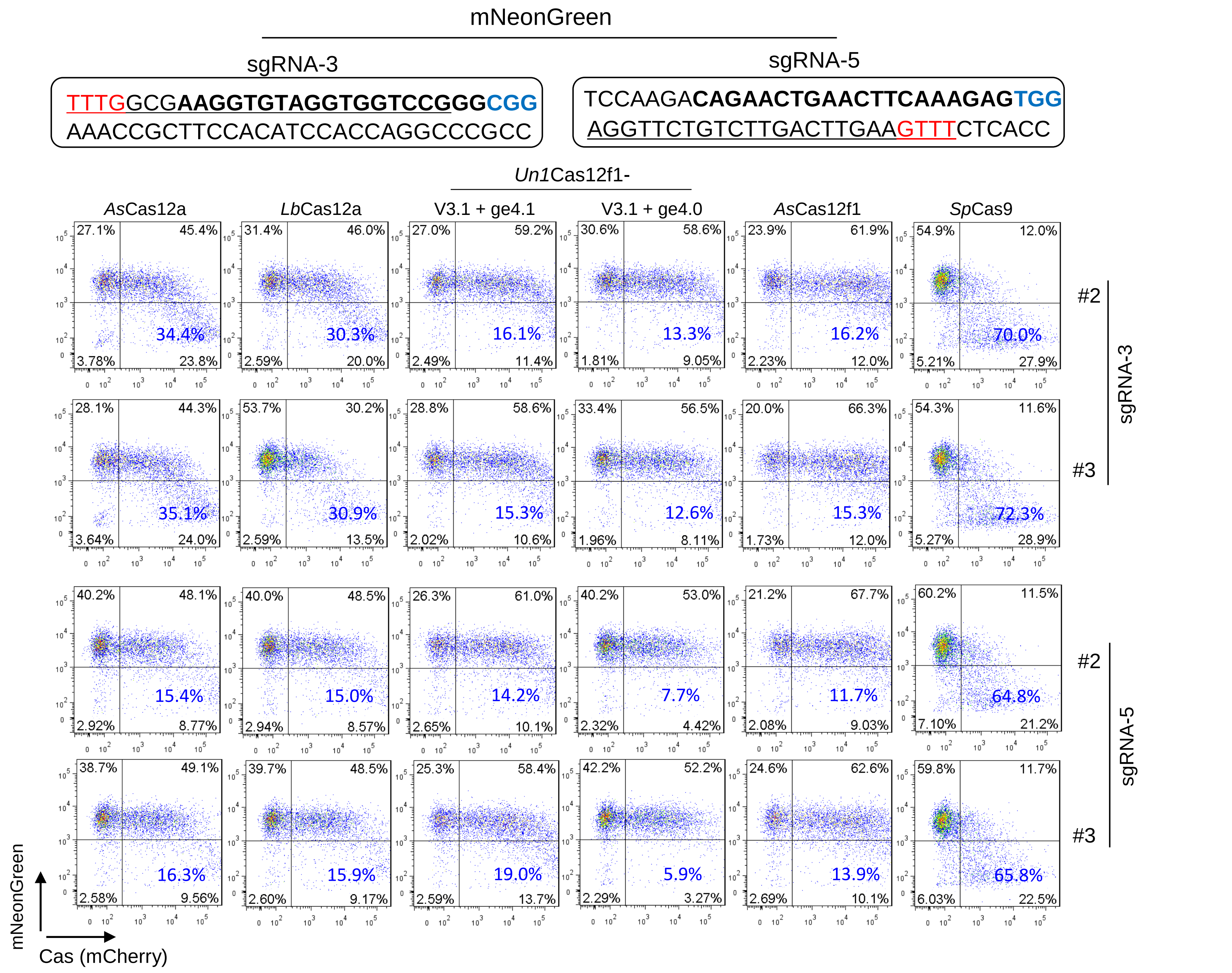

Figure S8 Activity comparison of the DNA editing CRISPR systems by FACS in HEK293T-KI-mNeonGreen reporter cells

Supplementary Figure 8. Activity comparison of the DNA editing CRISPR system by FACS in HEK293T-KI-mNeonGreen reporter cells. Another two replicates for the Fig. 3c. The editing efficiency (values were showed with blue) was determined as the proportion of GFP negative cells within the Cas-nucleases transfected cells (mCherry-positive) by FACS. mNeonGree-sgRNA3/5, DNA editing CRISPR targeting mNeonGreen site 3/5. Red sequences showing the PAM of Cas12f/Cas12a, Blue sequences showing the PAM of Cas9. n=3 independent experiments.

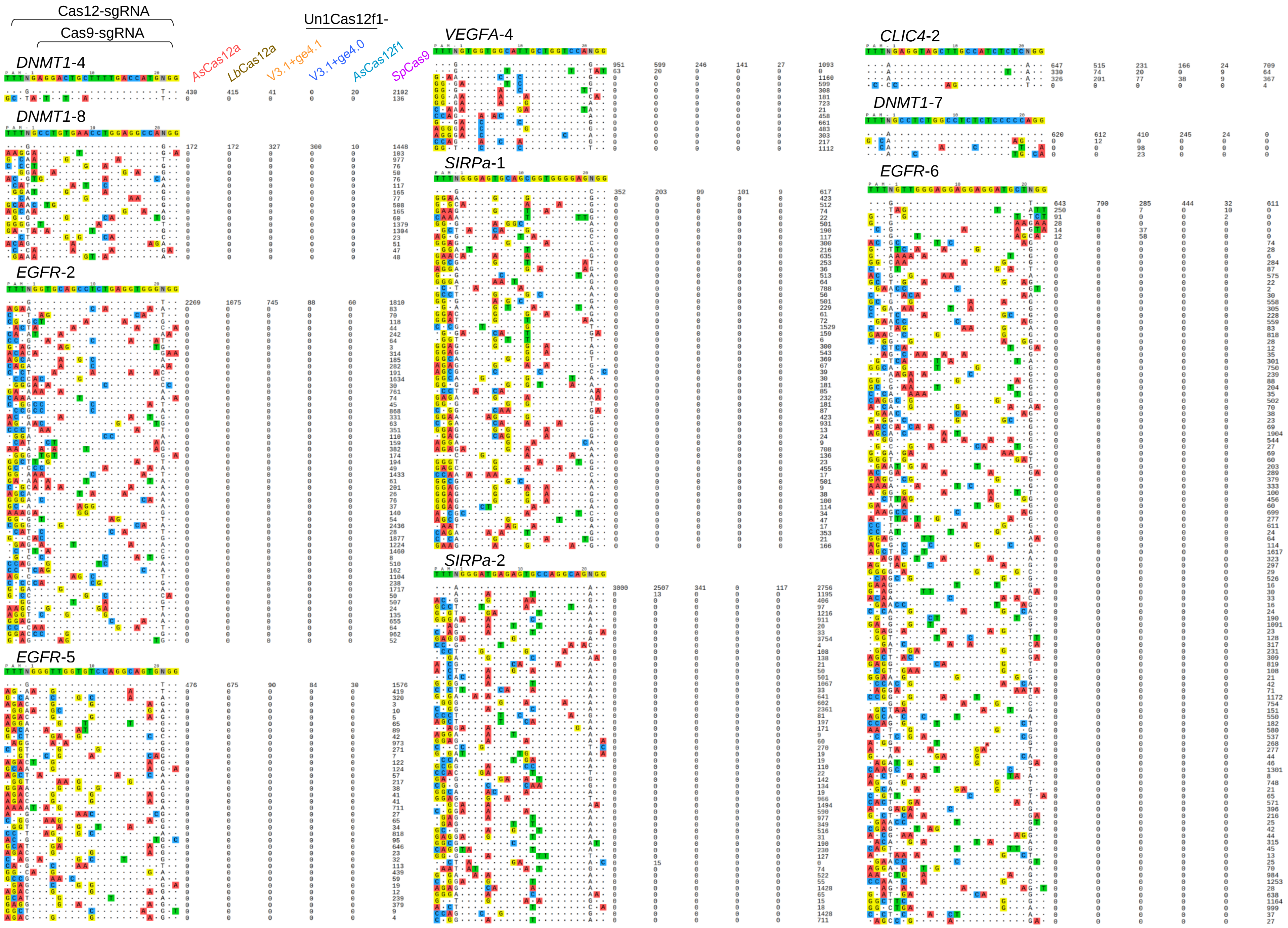

To be continued

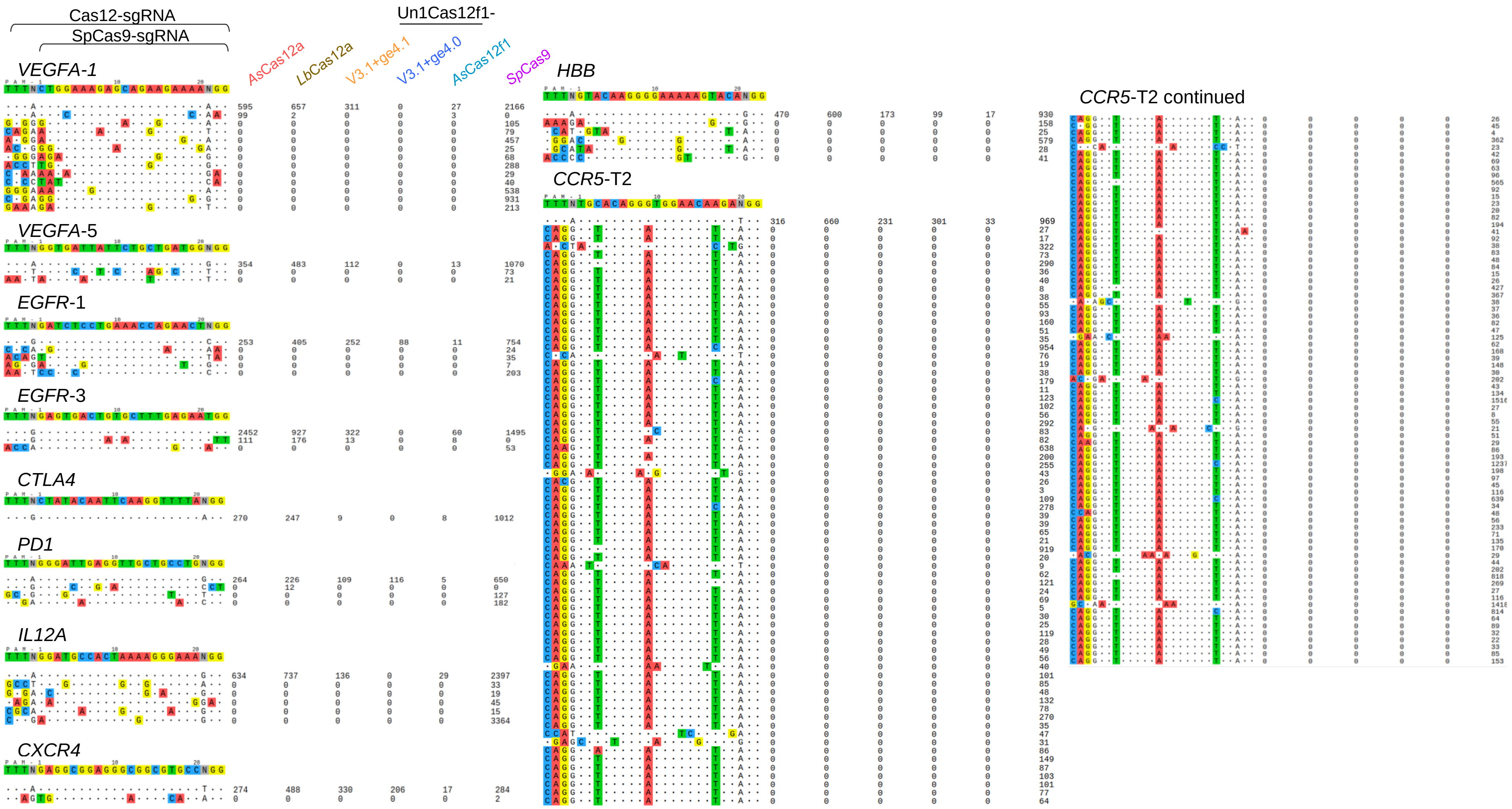

Fig. S9 Specificity comparison of the DNA editing CRISPR systems in HEK293T cells

Supplementary Figure 9. Specificity comparison of the DNA editing CRISPR systems in HEK293T cells. HEK293T cells were transfected by PEI method with the plasmids expressing DNA-editing nucleases, the corresponding sgRNAs that pooled with twenty-one guides, and the Tag-oligo DNA sequence. Genomic DNA was harvested three days post-transfection for libraries construction and Tag-seq analysis. Read counts represented a measure of cleavage frequency at a given site, mismatched positions within the spacer or PAM are highlighted in color (also see Fig. 2b). The targeted sites for Cas12a, Cas12f1 and *Sp*Cas9 share with a common spacer sequence as shown in the top.

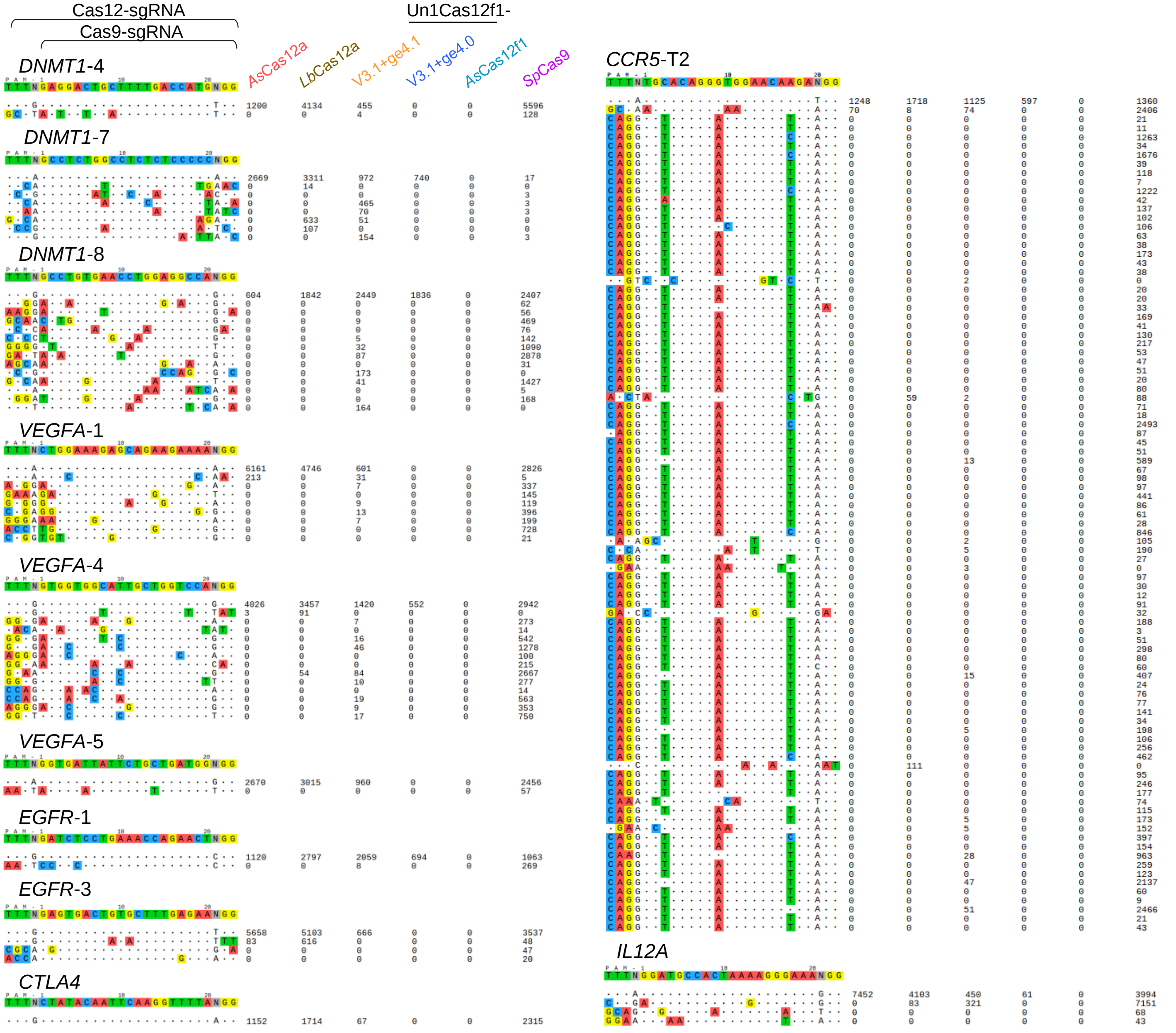

To be continued

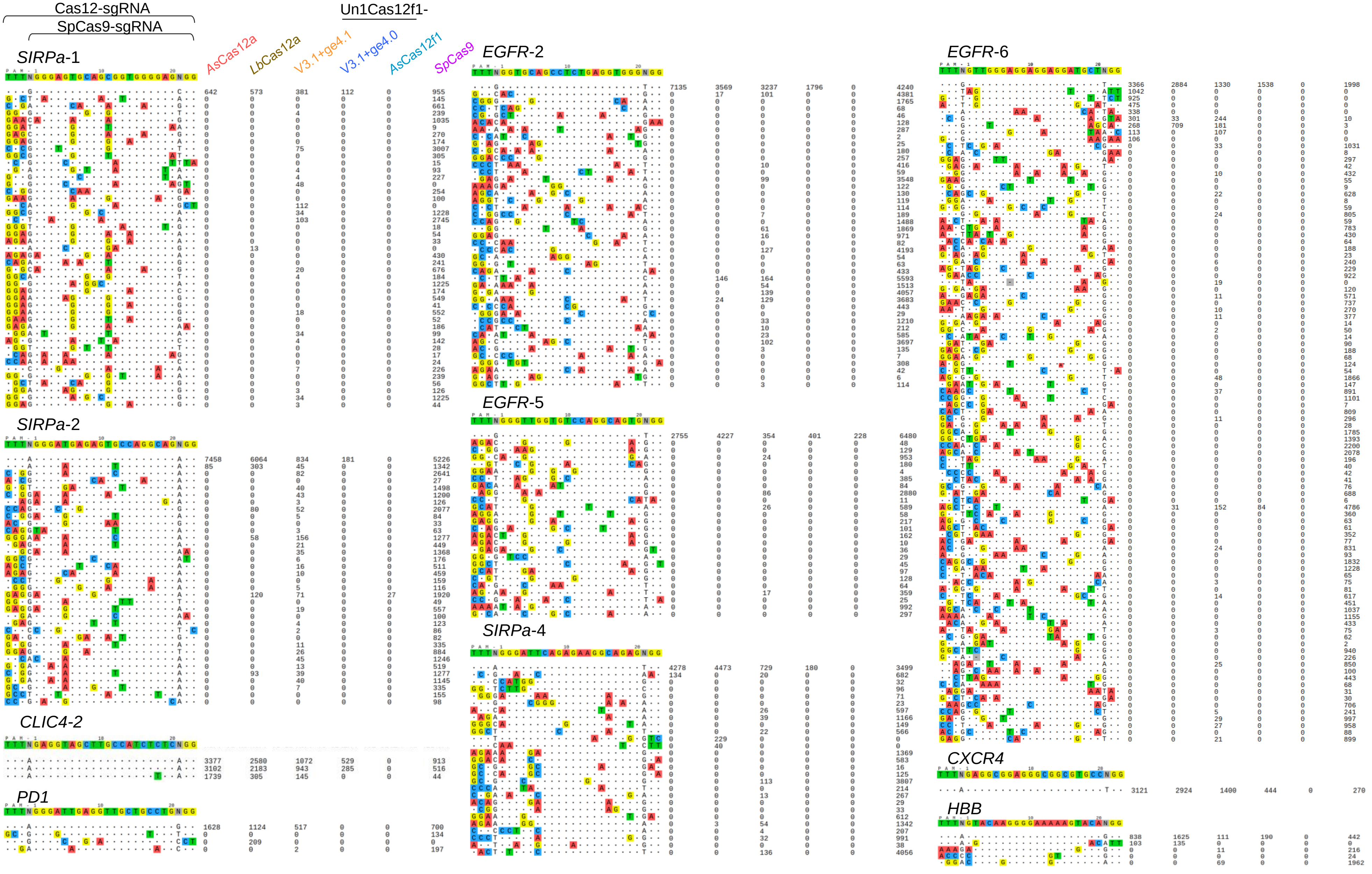

Fig. S10 Specificity comparison of the DNA editing CRISPR systems in MCF7 cells

Supplementary Figure 10. Specificity comparison of the DNA editing CRISPR systems in MCF7 cells. MCF7 cells were transfected by PEI method with the plasmids expressing DNA-editing nucleases, the corresponding sgRNAs that pooled with twenty-one guides, and the Tag-oligo DNA sequence. Genomic DNA was harvested three days post-transfection for libraries construction and Tag-seq analysis. Read counts represented a measure of cleavage frequency at a given site, mismatched positions within the spacer or PAM are highlighted in color. The targeted sites for Cas12a, Cas12f1 and *Sp*Cas9 share with a common spacer sequence as shown in the top.

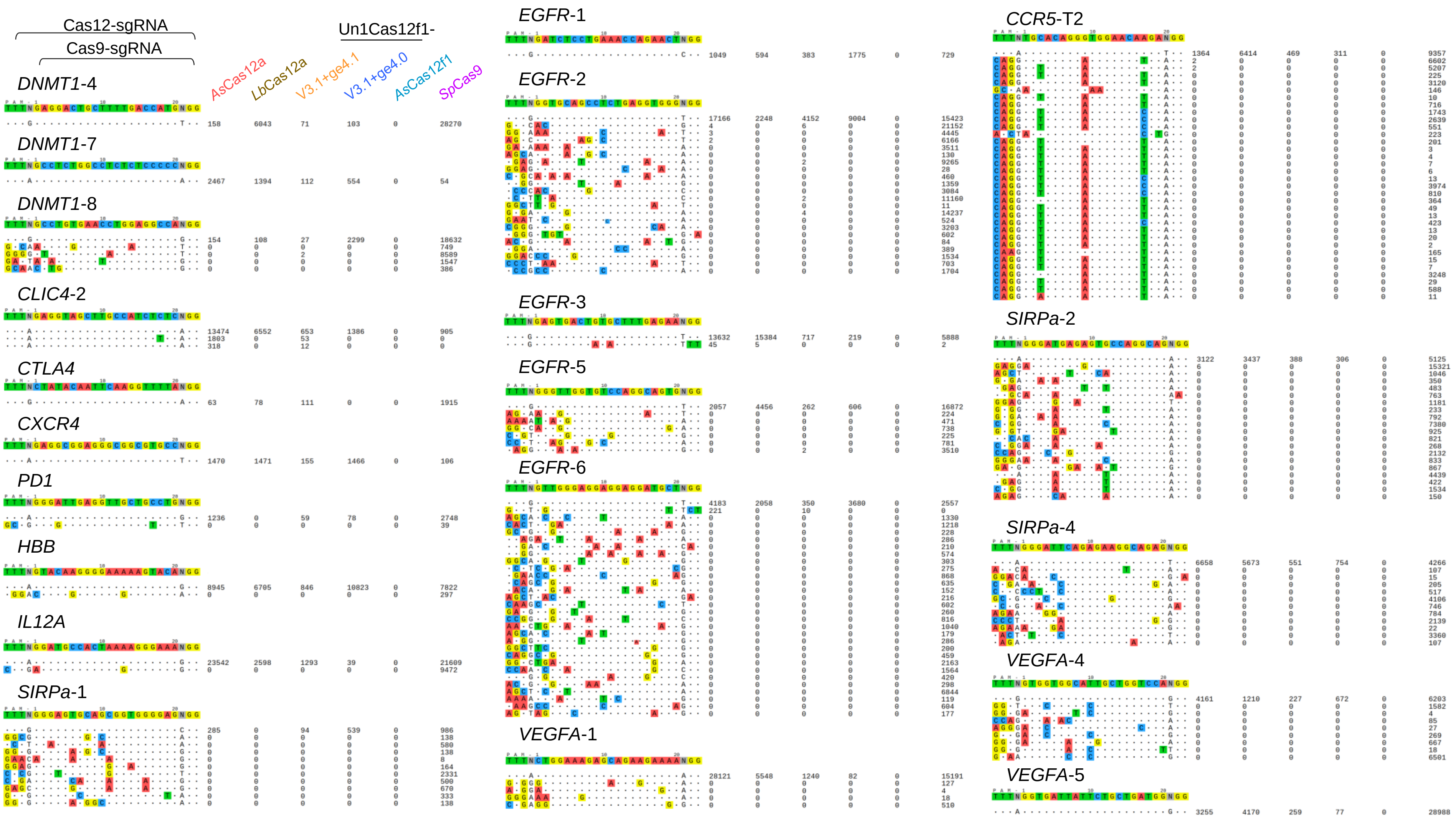

Fig. S11 Specificity comparison of the DNA editing CRISPR systems in K562 cells

Supplementary Figure 11. Specificity comparison of the DNA editing CRISPR systems in K562 cells. K562 cells were co-transfected by Lonza electroporation method with the plasmids expressing DNA-editing nucleases, the corresponding sgRNAs that pooled with twenty-one guides, and the Tag-oligo DNA sequence. Genomic DNA was harvested three days post-transfection for libraries construction and Tag-seq analysis. Read counts represented a measure of cleavage frequency at a given site, mismatched positions within the spacer or PAM are highlighted in color. The targeted sites for Cas12a, Cas12f1 and *Sp*Cas9 share a common spacer sequence as shown in the top.

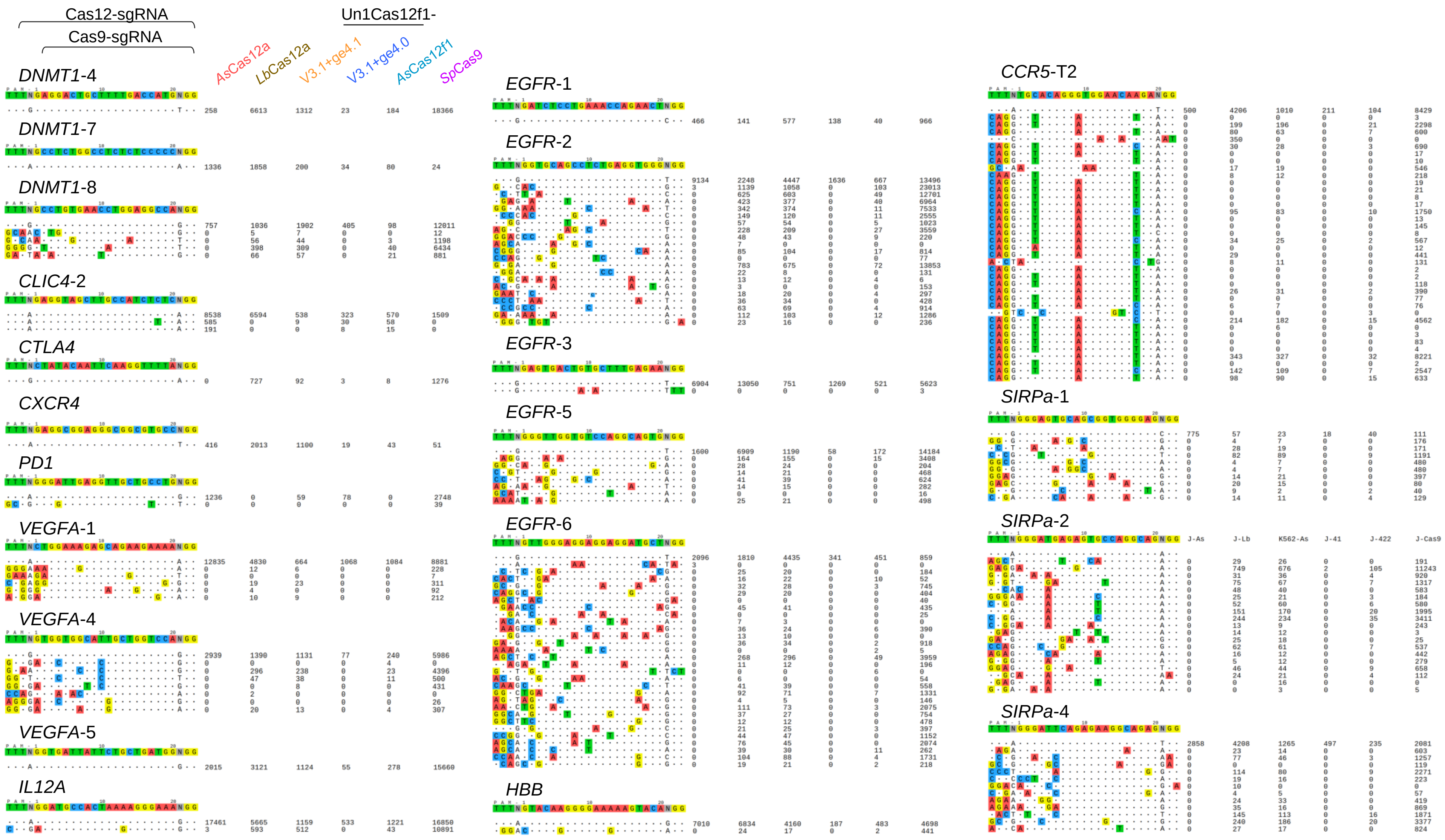

Fig. S12 Specificity comparison of the DNA editing CRISPR systems in Jurkat cells

Supplementary Figure 12. Specificity comparison of the DNA editing CRISPR systems in Jurkat cells. Jurkat cells were transfected by Lonza electroporation method with the plasmids expressing DNA-editing nucleases, the corresponding sgRNAs that pooled with twenty-one guides, and the Tag-oligo DNA sequence. Genomic DNA was harvested three days post-transfection for libraries construction and Tag-seq analysis. Read counts represented a measure of cleavage frequency at a given site, mismatched positions within the spacer or PAM are highlighted in color. The targeted sites for Cas12a, Cas12f1 and *Sp*Cas9 share a common spacer sequence as shown in the top.
