# Supplementary material for: Comparison of DNA targeting CRISPR editors in human cells
Source: Cell Biosci. 2023 Jan 16;13:11. doi: 10.1186/s13578-023-00958-z (PMC9844007; doi:10.1186/s13578-023-00958-z)
Supplement: Supplementary file 2 — Additional file 2: Table S1. Summary of potential mismatched sites in the reference human genome for the 21sgRNAs examined by Tag-seq. Table S2. The sgRNAs and primers used in this study. Table S3. Deep-seq primers for this study. [file 13578_2023_958_MOESM2_ESM.pdf]

Supplementary Information

Table S1. Summary of potential mismatched sites in the reference human genome for the 21 sgRNAs examined by Tag-seq

Name	Sequence(5'-3')	mm<1	mm<2	mm<3
CCR5-T2	TTTATGCACAGGGTGGAACAAGATTGG	0	1	28
CLIC4-2	TTTAGAGGTAGCTTGCCATCTCTCAGG	1	0	8
CTLA4	TTTGCTATACAATTCAAGGTTTTAAGG	0	1	17
CXCR4	TTTAGAGGCGGAGGGCGGCGTGCCTGG	0	2	25
DNMT1-4	TTTG GAGGACTGCTTTTGACCATGTGG	0	2	61
DNMT1-7	TTTAGCCTCTGGCCTCTCTCCCCCAGG	1	5	54
DNMT1-8	TTTGGCCTGTGAACCTGGAGGCCAGGG	0	2	38
EGFR-1	TTTGGATCTCCTGAAACCAGAACTCGG	0	3	27
EGFR-2	TTTGGGTGCAGCCTCTGAGGTGGGTGG	1	5	49
EGFR-3	TTTGgagtgactgtgctttgagaatgg	1	3	31
EGFR-5	TTTGGGGTTGGTGTCCAGGCAGTGTGG	0	4	121
EGFR-6	TTTGGTTGGGAGGAGGAGGATGCTTGG	0	5	60
HBB	TTTAGTACAAGGGGAAAAAGTACAGGG	0	3	32
IL12A	TTTAGGATGCCACTAAAAGGGAAAGGG	0	1	5
PD1	TTTAGGGATTGAGGTTGCTGCCTGGGG	0	2	19
SIRPa-1	TTTGGGGAGTGCAGCGGTGGGGAGCGG	0	11	210
SIRPa-2	TTTAgggatgagagtgccaggcagagg	0	18	118
SIRPa-4	TTTAGGGATTCAGAGAAGGCAGAGTGG	1	9	63
VEGFA-1	TTTACTGGAAAGAGCAGAAGAAAAAGG	5	87	808
VEGFA-4	TTTGGTGGTGGCATTGCTGGTCCAGGG	0	2	33
VEGFA-5	TTTA GGTGATTATTCTGCTGATGGGGG	0	1	45

Note: mm=mismatch

Table S2. The sgRNAs and primers used in this study

Name	PAM	Sequence(5'-3')	location (hg19)			
BRAF-WT	TTTG	GTCTAGCTACAGTGAAATCT	chr7	-	140453129	140453152
CCR5-3	TTTA	GGATTCCCGAGTAGCAGATG	chr3	-	46415026	46415049
CCR5-T2	TTTA	TGCACAGGGTGGAACAAGAT	chr3	+	46414372	46414395
CLIC4	TTTA	CCCTGGCTACCTCCCCTACC	chr1	+	25167622	25167645
DNMT1-S7	TTTG	GCTCAGCAGGCACCTGCCTC	chr1	+	155081128	155081147
EMX1	TTTG	TCCTCCGGTTCTGGAACCAC	chr2	-	73160924	73160947
IFN3	TTTA	CCAGGGCGAAGTGGGGAGGT	chr12	-	68553807	68553830
IL12A	TTTA	GGATGCCACTAAAAGGGAAA	chr3	-	159817128	159817151
NLRC4	TTTA	GAGGGAGACACAAGTTGATA	chr2	-	32453505	32453528
P2RX5-TAX1BP3	TTTA	CACATAGGCCATTCAGAAAC	chr17	+	3573073	3573096
PCSK9-24	TTTG	CCCAGAGCATCCCGTGGAAC	chr1	+	55512244	55512267
PD1-20	TTTG	CTGTGAGCTCTAGTCCCCAC	chr2	-	242795707	242795730
PRKCH	TTTG	GGGACGGGGAGAAGGAAAAG	chr14	-	61916066	61916089
RPL32P3	TTTG	GGGTGATCAGACCCAACAGC	chr3	-	129108496	129108519
RUNX1-S1	TTTA	TGTCTCTCTGCCCCCTCCCC	chr21	+	37371826	37371849
SIRPa-S2	TTTG	CTACAGTTACAACATTCCAA	chr20	+	1902671	1902694
site1	TTTG	CACACACACAGTGGGCTACC	chr3	-	119947200	119947223

site2	TTTA	AGAACACATACCCCTGGGCC	chr5 - 170967061 170967084
VEGFA-1	TTTA	CTGGAAAGAGCAGAAGAAAA	chr6 - 43742345 43742368
VEGFA-2	TTTG	CTCTCAAGACCCACAATCCA	chr6 + 43671810 43671833
VEGFA-3	TTTG	CTCCTGGACCCCCTATTTCT	chr6 - 43737278 43737301
Shared sgRNAs for Cas12 and Cas9. Red is the PAM of Cas9			
Name	PAM	Sequence(5’-3’)	location (hg19)
CCR5-T2	TTTA	TGCACAGGGTGGAACAAGATGG	chr3 + 46414372 46414397
CLIC4-2	TTTA	GAGGTAGCTTGCCATCTCTCAGG	chr1 + 25167695 25167721/<br>chr9 - 22747627 22747653
CTLA4	TTTG	CTATACAATTCAAGGTTTTAAGG	chr2 + 204733189 204733215
CXCR4	TTTA	GAGGCGGAGGGCGGCGTGCCTGG	chr2 - 136875173 136875199
DNMT1-4	TTTG	GAGGACTGCTTTTGACCATGTGG	chr19 - 10302098 10302124
DNMT1-7	TTTA	GCCTCTGGCCTCTCTCCCCCAGG	chr19 - 10251585 10251611
DNMT1-8	TTTG	GCCTGTGAACCTGGAGGCCAGGG	chr19 - 10247673 10247699
EGFR-1	TTTG	GATCTCCTGAAACCAGAACTCGG	chr7 - 55089650 55089676
EGFR-2	TTTG	GGTGCAGCCTCTGAGGTGGGTGG	chr7 + 55168137 55168163
EGFR-3	tttg	gagtgactgtgctttgagaatgg	chr7 - 55176422 55176448
EGFR-5	TTTG	GGGTTGGTGTCCAGGCAGTGTGG	chr7 - 55289682 55289708
EGFR-6	TTTG	GTTGGGAGGAGGAGGATGCTTGG	chr7 - 55316782 55316808
HBB	TTTA	GTACAAGGGGAAAAAGTACAGGG	chr11 - 5249397 5249423
IL12A	TTTA	GGATGCCACTAAAAGGGAAAGGG	chr3 - 159817125 159817151
PD1	TTTA	GGGATTGAGGTTGCTGCCTGGGG	chr2 + 242793942 242793968
SIRPa-1	TTTG	GGGAGTGCAGCGGTGGGGAGCGG	chr20 - 1876185 1876211
SIRPa-2	ttta	gggatgagagtgccaggcagagg	chr20 + 1887164 1887190
SIRPa-4	TTTA	GGGATTCAGAGAAGGCAGAGTGG	chr20 - 1911139 1911165
VEGFA-1	TTTA	CTGGAAAGAGCAGAAGAAAAAGG	chr6 - 43742342 43742368
VEGFA-4	TTTG	GTGGTGGCATTGCTGGTCCAGGG	chr6 + 43748718 43748744
VEGFA-5	TTTA	GGTGATTATTCTGCTGATGGGGG	chr6 + 43751287 43751313
sgRNA for the disruption of mNeonGreen			
mNeonGreen-sgRNA3 (Cas12)	TTTG	GCGAAGGTGTAGGTGGTCCG	
mNeonGreen-sgRNA5 (Cas12)	TTTG	AAGTTCAGTTCTGTCTTGGA	
mNeonGreen-sgRNA3 (Cas9)	CGG	AAGGTGTAGGTGGTCCGGG	
mNeonGreen-sgRNA5 (Cas9)	TGG	CAGAACTGAACTTCAAAGAG	
sgRNA for gene activation			
Cas12f1-HBG	TTTG	CCTTGTCAAGGCTATTGGTC	
Cas12f1-IL1RN	TTTG	GTTTCTGCTAGCCTGAGTCA	
Cas12a-HBG	TTTA	AACTACAGGCCTCACTGGAGCTA	
Cas12a-IL1RN	TTTC	CAGGAGGGTGACTCAGGCTAGCA	
Cas9-HBG	AGG	GCTAGGGATGAAGAATAAA	
Cas9-IL1RN	TGG	ACGCAGATAAGAACCAGTT	
Cas12f1-Fgf21	TTTG	ACACACTTGGCAGGAACCTG	
Cas12a-Fgf21	TTTG	ACACACTTGGCAGGAACCTGAAT	

Cas9-Fgf21	CGG	GAAT TCCACCGTGGCCAGGG	
qPCR primers			
qPCR-IL1RN-F	GGAATCCATGGAGGGAAGAT		
qPCR-IL1RN-R	TGTTCTCGCTCAGGTCAGTG		
qPCR-HBG-F	GCTGAGTGAACTGCACTGTGA		
qPCR-HBG-R	GAATTCTTTGCCGAAATGGA		
qPCR-Fgf21-F	CAAGACACTGAAGCCCACCT		
qPCR-Fgf21-R	CACCCAGGATTTGAATGACC		
qPCR-hGAPDH-F	AGAAGGCTGGGGCTCATTTG		
qPCR-hGAPDH-R	AGGGGCCATCCACAGTCTTC		
qPCR-mGapdh-F1	GGTGAAGGTCGGTGTGAAC		
qPCR-mGapdh-R1	GAGTGGAGTCATACTGGAAC		

Table S3. Deep-seq primers for this study

Name	Sequence(5'-3')
CCR5-T2-DS-F1	gtCTCTTTCCCTACACGACGCTCTTCCGATCTTAGATCGGAGGGCAACTAAATACATTC
CCR5-T2-DS-R1	tgACTGGAGTTCAGACGTGTGCTCTTCCGATCTTAAGGCTTCCTGGGAGAGACGCAAAC
CCR5-T2-DS-F2	gtCTCTTTCCCTACACGACGCTCTTCCGATCTCAGAGCGGAGGGCAACTAAATACATTC
CCR5-T2-DS-R2	acACTGGAGTTCAGACGTGTGCTCTTCCGATCTCTCTACTTCCTGGGAGAGACGCAAAC
CLIC4-2-1-DS-F1	tgCTCTTTCCCTACACGACGCTCTTCCGATCTTAGATCCCCGGGTTCCCCTCTCTTTA
CLIC4-2-1-DS-R1	tACTGGAGTTCAGACGTGTGCTCTTCCGATCTTAAGGCTGATACAGTAGCAATCCTTTC
CLIC4-2-1-DS-F2	gtCTCTTTCCCTACACGACGCTCTTCCGATCTCAGAGCCCCGGGTTCCCCTCTCTTTA
CLIC4-2-1-DS-R2	cACTGGAGTTCAGACGTGTGCTCTTCCGATCTCTCTACTGATACAGTAGCAATCCTTTC
CLIC4-2-2-DS-F1	gCTCTTTCCCTACACGACGCTCTTCCGATCTTAGATCTGTTGAAAGTTATGTTCCCCTC
CLIC4-2-2-DS-R1	aACTGGAGTTCAGACGTGTGCTCTTCCGATCTTAAGGCTTAGACAGTATTCGGTGCCCA
CLIC4-2-2-DS-F2	cCTCTTTCCCTACACGACGCTCTTCCGATCTCAGAGCTGTTGAAAGTTATGTTCCCCTC
CLIC4-2-2-DS-R2	cACTGGAGTTCAGACGTGTGCTCTTCCGATCTCTCTACTTAGACAGTATTCGGTGCCCA
CTLA4-DS-F1	cCTCTTTCCCTACACGACGCTCTTCCGATCTTAGATCAGGATGAGCTCACAAGTTCCTT
CTLA4-DS-R1	tACTGGAGTTCAGACGTGTGCTCTTCCGATCTTAAGGCCCACACGTAGGTCAGGCAAC
CTLA4-DS-F2	gCTCTTTCCCTACACGACGCTCTTCCGATCTCAGAGCAGGATGAGCTCACAAGTTCCTT
CTLA4-DS-R2	cACTGGAGTTCAGACGTGTGCTCTTCCGATCTCTCTACCCACACGTAGGTCAGGCAAC
CXCR4-DS-F1	cgCTCTTTCCCTACACGACGCTCTTCCGATCTTAGATCGGGATGTCTTGGAGCGAGTT
CXCR4-DS-R1	ctACTGGAGTTCAGACGTGTGCTCTTCCGATCTTAAGGCCCAAACGGTTTCCCCACTTG
CXCR4-DS-F2	cgCTCTTTCCCTACACGACGCTCTTCCGATCTCAGAGCGGGATGTCTTGGAGCGAGTT
CXCR4-DS-R2	ctACTGGAGTTCAGACGTGTGCTCTTCCGATCTCTCTACCCAAACGGTTTCCCCACTTG
DNMT1-4-DS-F1	cgCTCTTTCCCTACACGACGCTCTTCCGATCTTAGATCTACAATCCACCACTGACCCTG
DNMT1-4-DS-R1	cACTGGAGTTCAGACGTGTGCTCTTCCGATCTTAAGGCGTTTCAAGTACTGTTGGATAC
DNMT1-4-DS-F2	cgCTCTTTCCCTACACGACGCTCTTCCGATCTCAGAGCTACAATCCACCACTGACCCTG
DNMT1-4-DS-R2	cACTGGAGTTCAGACGTGTGCTCTTCCGATCTCTCTACGTTTCAAGTACTGTTGGATAC
DNMT1-7-DS-F1	cgCTCTTTCCCTACACGACGCTCTTCCGATCTTAGATCGCTTGAGAGCAAGGTGGACTT
DNMT1-7-DS-R1	tACTGGAGTTCAGACGTGTGCTCTTCCGATCTTAAGGCGGGTTGGAAGTCGTTTCAGGT
DNMT1-7-DS-F2	cgCTCTTTCCCTACACGACGCTCTTCCGATCTCAGAGCGCTTGAGAGCAAGGTGGACTT
DNMT1-7-DS-R2	tACTGGAGTTCAGACGTGTGCTCTTCCGATCTCTCTACGGGTTGGAAGTCGTTTCAGGT
DNMT1-8-DS-F1	cgCTCTTTCCCTACACGACGCTCTTCCGATCTTAGATCGCGATCTGCCCAACATCGAG
DNMT1-8-DS-R1	tACTGGAGTTCAGACGTGTGCTCTTCCGATCTTAAGGCGTCAGCAGCTACCTTCTCTGG
DNMT1-8-DS-F2	cgCTCTTTCCCTACACGACGCTCTTCCGATCTCAGAGCGCGATCTGCCCAACATCGAG
DNMT1-8-DS-R2	tACTGGAGTTCAGACGTGTGCTCTTCCGATCTCTCTACGTCAGCAGCTACCTTCTCTGG

EGFR-1-DS-F1	aCTCTTTCCCTACACGACGCTCTTCCGATCTTAGATCCCTTGCCAGCTCTGACATTAGA
EGFR-1-DS-R1	cACTGGAGTTCAGACGTGTGCTCTTCCGATCTTAAGGCTGGTTCTGGGACATGCTAACT
EGFR-1-DS-F2	tCTCTTTCCCTACACGACGCTCTTCCGATCTCAGAGCCCTTGCCAGCTCTGACATTAGA
EGFR-1-DS-R2	gACTGGAGTTCAGACGTGTGCTCTTCCGATCTCTCTACTGGTTCTGGGACATGCTAACT
EGFR-2-DS-F1	cgCTCTTTCCCTACACGACGCTCTTCCGATCTTAGATCCCCAGCAGATGCTGTGTGTA
EGFR-2-DS-R1	ctACTGGAGTTCAGACGTGTGCTCTTCCGATCTTAAGGCGCCAGCCCTCCAAGACTAAC
EGFR-2-DS-F2	cgCTCTTTCCCTACACGACGCTCTTCCGATCTCAGAGCCCCAGCAGATGCTGTGTGTA
EGFR-2-DS-R2	ctACTGGAGTTCAGACGTGTGCTCTTCCGATCTCTCTACGCCAGCCCTCCAAGACTAAC
EGFR-3-DS-F1	cCTCTTTCCCTACACGACGCTCTTCCGATCTTAGATCTATTCTGCCACCTGGAGTCCTA
EGFR-3-DS-R1	cACTGGAGTTCAGACGTGTGCTCTTCCGATCTTAAGGCAACTCATGTTCGGAACTGTCC
EGFR-3-DS-F2	gCTCTTTCCCTACACGACGCTCTTCCGATCTCAGAGCTATTCTGCCACCTGGAGTCCTA
EGFR-3-DS-R2	cACTGGAGTTCAGACGTGTGCTCTTCCGATCTCTCTACAACTCATGTTCGGAACTGTCC
EGFR-5-DS-F1	cgCTCTTTCCCTACACGACGCTCTTCCGATCTTAGATCCTGGTCTACCCCAGAGCTGA
EGFR-5-DS-R1	cACTGGAGTTCAGACGTGTGCTCTTCCGATCTTAAGGCTTGGGGGAATAGAGACAACGG
EGFR-5-DS-F2	cgCTCTTTCCCTACACGACGCTCTTCCGATCTCAGAGCCTGGTCTACCCCAGAGCTGA
EGFR-5-DS-R2	cACTGGAGTTCAGACGTGTGCTCTTCCGATCTCTCTACTTGGGGGAATAGAGACAACGG
EGFR-6-DS-F1	gCTCTTTCCCTACACGACGCTCTTCCGATCTTAGATCCCATAGGACTTGGCTCACAACC
EGFR-6-DS-R1	cACTGGAGTTCAGACGTGTGCTCTTCCGATCTTAAGGCACCCAAGGAGGTCCTATTCAC
EGFR-6-DS-F2	cCTCTTTCCCTACACGACGCTCTTCCGATCTCAGAGCCCATAGGACTTGGCTCACAACC
EGFR-6-DS-R2	cACTGGAGTTCAGACGTGTGCTCTTCCGATCTCTCTACACCCAAGGAGGTCCTATTCAC
HBB-DS-F1	gCTCTTTCCCTACACGACGCTCTTCCGATCTTAGATCTGCACTTCAAAAGTTTTTCCTC
HBB-DS-R1	tACTGGAGTTCAGACGTGTGCTCTTCCGATCTTAAGGCTGGGGTAATCAGTGGTGTCAA
HBB-DS-F2	cCTCTTTCCCTACACGACGCTCTTCCGATCTCAGAGCTGCACTTCAAAAGTTTTTCCTC
HBB-DS-R2	aACTGGAGTTCAGACGTGTGCTCTTCCGATCTCTCTACTGGGGTAATCAGTGGTGTCAA
IL12A-DS-F1	cgCTCTTTCCCTACACGACGCTCTTCCGATCTTAGATCCCATCTGGGTTTCTGTGGCAT
IL12A-DS-R1	ctACTGGAGTTCAGACGTGTGCTCTTCCGATCTTAAGGCGCAACCCTGTAACCACAGTC
IL12A-DS-F2	cgCTCTTTCCCTACACGACGCTCTTCCGATCTCAGAGCCCATCTGGGTTTCTGTGGCAT
IL12A-DS-R2	ctACTGGAGTTCAGACGTGTGCTCTTCCGATCTCTCTACGCAACCCTGTAACCACAGTC
PD1-DS-F1	cgCTCTTTCCCTACACGACGCTCTTCCGATCTTAGATCCTGCTTACCCTGAGCTGTCC
PD1-DS-R1	ctACTGGAGTTCAGACGTGTGCTCTTCCGATCTTAAGGCGGCCTGCAGGACTCACATTC
PD1-DS-F2	cgCTCTTTCCCTACACGACGCTCTTCCGATCTCAGAGCCTGCTTACCCTGAGCTGTCC
PD1-DS-R2	ctACTGGAGTTCAGACGTGTGCTCTTCCGATCTCTCTACGGCCTGCAGGACTCACATTC
PTEN-DS-F1	gCTCTTTCCCTACACGACGCTCTTCCGATCTTAGATCCTGTATTAGTGGCATCACAAGT
PTEN-DS-R1	tACTGGAGTTCAGACGTGTGCTCTTCCGATCTTAAGGCTGGCGGTGTCATAATGTCTTT
PTEN-DS-F2	cCTCTTTCCCTACACGACGCTCTTCCGATCTCAGAGCCTGTATTAGTGGCATCACAAGT
PTEN-DS-R2	tACTGGAGTTCAGACGTGTGCTCTTCCGATCTCTCTACTGGCGGTGTCATAATGTCTTT
SIRPa-1-DS-F1	cgCTCTTTCCCTACACGACGCTCTTCCGATCTTAGATCATTAGCCGAAGGGGTCCCAG
SIRPa-1-DS-R1	ctACTGGAGTTCAGACGTGTGCTCTTCCGATCTTAAGGCAGCCTTAGTCATTTCCCCGC
SIRPa-1-DS-F2	cgCTCTTTCCCTACACGACGCTCTTCCGATCTCAGAGCATTAGCCGAAGGGGTCCCAG
SIRPa-1-DS-R2	ctACTGGAGTTCAGACGTGTGCTCTTCCGATCTCTCTACAGCCTTAGTCATTTCCCCGC
SIRPa-2-DS-F1	cgCTCTTTCCCTACACGACGCTCTTCCGATCTTAGATCACAGAATGGCCAAGGAAGGC
SIRPa-2-DS-R1	ctACTGGAGTTCAGACGTGTGCTCTTCCGATCTTAAGGCCATCCGTGTTCCACAACCCA
SIRPa-2-DS-F2	cgCTCTTTCCCTACACGACGCTCTTCCGATCTCAGAGCACAGAATGGCCAAGGAAGGC
SIRPa-2-DS-R2	ctACTGGAGTTCAGACGTGTGCTCTTCCGATCTCTCTACCATCCGTGTTCCACAACCCA
SIRPa-4-DS-F1	cgCTCTTTCCCTACACGACGCTCTTCCGATCTTAGATCTAGATGGGGCTGTGTAGCCA
SIRPa-4-DS-R1	ctACTGGAGTTCAGACGTGTGCTCTTCCGATCTTAAGGCACAGTGGGCTCTGTGAATGG
SIRPa-4-DS-F2	cgCTCTTTCCCTACACGACGCTCTTCCGATCTCAGAGCTAGATGGGGCTGTGTAGCCA

SIRPa-4-DS-R2	ctACTGGAGTTCAGACGTGTGCTCTTCCGATCTCTCTACACAGTGGGCTCTGTGAATGG
VEGFA-1-DS-F1	cgCTCTTTCCCTACACGACGCTCTTCCGATCTTAGATCACACTGTCTTCCTGCTCTGTG
VEGFA-1-DS-R1	ctACTGGAGTTCAGACGTGTGCTCTTCCGATCTTAAGGCGACTTGGAGAAGCCAGAGGC
VEGFA-1-DS-F2	cgCTCTTTCCCTACACGACGCTCTTCCGATCTCAGAGCACACTGTCTTCCTGCTCTGTG
VEGFA-1-DS-R2	ctACTGGAGTTCAGACGTGTGCTCTTCCGATCTCTCTACGACTTGGAGAAGCCAGAGGC
VEGFA-4-DS-F1	cgCTCTTTCCCTACACGACGCTCTTCCGATCTTAGATCTCTCATCCTCCTGGCCCGT
VEGFA-4-DS-R1	ctACTGGAGTTCAGACGTGTGCTCTTCCGATCTTAAGGCGCTTCCAGACGAGTGCAGAA
VEGFA-4-DS-F2	cgCTCTTTCCCTACACGACGCTCTTCCGATCTCAGAGCTCTCATCCTCCTGGCCCGT
VEGFA-4-DS-R2	ctACTGGAGTTCAGACGTGTGCTCTTCCGATCTCTCTACGCTTCCAGACGAGTGCAGAA
VEGFA-5-DS-F1	cCTCTTTCCCTACACGACGCTCTTCCGATCTTAGATCCCAGGGGTCCTTGGGAAAGATA
VEGFA-5-DS-R1	ctACTGGAGTTCAGACGTGTGCTCTTCCGATCTTAAGGCGAGACAATTCGGCGGCTTCA
VEGFA-5-DS-F2	gCTCTTTCCCTACACGACGCTCTTCCGATCTCAGAGCCCAGGGGTCCTTGGGAAAGATA
VEGFA-5-DS-R2	ctACTGGAGTTCAGACGTGTGCTCTTCCGATCTCTCTACGAGACAATTCGGCGGCTTCA

DNA sequences

- Expression of the ge3.0 sgRNA：

Red: U6 promoter, blue: ge3 scaffold

gagggcctatttcccatgattccttcatatttgcatatacgatacaaggctgttagagagataattagaattaatttgactgtaaacacaaagatattagtacaaaatacgtgacgtagaaagtaataatttcttgggtagtttgcagttttaaaattatgttttaaaatggactatcatatgcttaccgtaacttgaaagtatttcgatttcttggctttatatatcttgtggaaaggacgaaacaccgACCGCTTCACCAAAAGCTGTCCCTTAGGGGATTAGAACTTGAGTGAAGGTGGGCTGCTTGCATCAGCCTAATGTCGAGAAGTGCTTTCTTCGGAAAGTAACCCTCGAAACAAATTCAGTGCTCCTCTCCAATTCTGCACAAGAAAGTTGCAGAACCCGAATAGAGCAATGAAGGAATGCAAC/20bp-oligo/ttttattttt

- Expression of the ge4.0 sgRNA：

Red: U6 promoter, blue: ge4 scaffold

gagggcctatttcccatgattccttcatatttgcatatacgatacaaggctgttagagagataattagaattaatttgactgtaaacacaaagatattagtacaaaatacgtgacgtagaaagtaataatttcttgggtagtttgcagttttaaaattatgttttaaaatggactatcatatgcttaccgtaacttgaaagtatttcgatttcttggctttatatatcttgtggaaaggacgaaacaccgACCGCTTCACCAAAAGCTGTCCCTTAGGGGATTAGAACTTGAGTGAAGGTGGGCTGCTTGCATCAGCCTAATGTCGAGAAGTGCTTTCTTCGGAAAGTAACCCTCGAAACAAAgaaGGAATGCAAC/20bp-oligo/ttttattttt

- Expression of the ge4.1 sgRNA：

Red: U6 promoter, blue: ge4.1 scaffold

gagggcctatttcccatgattccttcatatttgcatatacgatacaaggctgttagagagataattagaattaatttgactgtaaacacaaagatattagtacaaaatacgtgacgtagaaagtaataatttcttgggtagtttgcagttttaaaattatgttttaaaatggactatcatatgcttaccgtaacttgaaagtatttcgatttcttggctttatatatcttgtggaaaggacgaaacaccgACCGCTTCACTTAGAGTGAAGGTGGGCTGCTTGCATCAGCCTAATGTCGAGAAGTGCTTTCTTCGGAAAGTAACCCTCGAAACAAAgaaGGAATGCAAC/20bp-oligo/ttttattttt

- Expression of the AsCas12f1 sgRNA：

Red: U6 promoter, blue: AsCas12f1 sgRNA scaffold

gagggcctatttcccatgattccttcatatttgcatatacgatacaaggctgttagagagataattagaattaatttgactgtaaacacaaagatattagtacaaaatacgtgacgtagaaagtaataatttcttgggtagtttgcagttttaaaattatgttttaaaatggactatcatatgcttaccgtaacttgaaagtatttcgatttcttggctttatatatcttgtggaaaggacgaaacaccgATTCGTCGGTTCAGCGACGATAAGCCGAGAAGTGCCAATAAAACTGTTAAGTGGTTTGGTAACGCTCGGTAAGGTAGCCAAAAGGCTGAAACTCCGTGCACAAAGACCGCACGGACGCTTCACATATAGCTCATAAACAAGGGTTTGCGAGCTAGCTTGTGGAGTGTGAAC/20bp-oligo/tttttt

- Expression of the LbCas12a sgRNA：

Red: U6 promoter, blue: LbCas12a sgRNA scaffold

gagggcctatttcccatgattccttcatatttgcatatacgatacaaggctgttagagagataattagaattaatttgactgtaaacacaaagatattagtacaaaat
acgtgacgtagaaagtaataatttcttgggtagtttgcagttttaaaattatgttttaaaatggactatcatatgcttaccgtaacttgaaagtatttcgatttcttggcttt
atatatcttgtggaaaggacgaaacaccgAATTTCTACTAAGTGTAGAT/20bp-oligo/ttttattttt

- Expression of the AsCas12a sgRNA：

Red: U6 promoter, blue: AsCas12a sgRNA scaffold

gagggcctatttcccatgattccttcatatttgcatatacgatacaaggctgttagagagataattagaattaatttgactgtaaacacaaagatattagtacaaaat
acgtgacgtagaaagtaataatttcttgggtagtttgcagttttaaaattatgttttaaaatggactatcatatgcttaccgtaacttgaaagtatttcgatttcttggcttt
atatatcttgtggaaaggacgaaacaccgAATTTCTACTCTTGTAGAT/20bp-oligo/ttttattttt

- Expression of the Cas9 FE2.1 sgRNA：

Red: U6 promoter, blue: Cas9 FE2.1 scaffold

gagggcctatttcccatgattccttcatatttgcatatacgatacaaggctgttagagagataattagaattaatttgactgtaaacacaaagatattagtacaaaat
acgtgacgtagaaagtaataatttcttgggtagtttgcagttttaaaattatgttttaaaatggactatcatatgcttaccgtaacttgaaagtatttcgatttcttggcttt
atatatcttgtggaaaggacgaaacacc/g+19bp-oligo/GTTTAAGAGCTATGCTGGAAACAGCATAGCAAGTTTAAATAAGG
CTAGTCCGTTATCAACTTTGCTGGAAACAGCAAAGTGGCACCGAGTCGGTGCTTTTTTT

- Expression of the AsCas12f1 nuclease：

Orange: SV40 NLS, blue: AsCas12f1, violet: nucleoplasmin NLS, green: 3XHA, yellow: P2A, Red: mCherry

CCAAAGAAGAAGCGGAAGGTCATGATCAAGGTGTACAGATACGAGATCGTGAAGCCTCTGGACCTGGACTG
GAAGGAGTTCGGCACCATCCTGAGACAGCTGCAGCAGGAAACCAGATTCGCCCTGAATAAGGCCACACAGC
TGGCCTGGGAGTGGATGGGCTTCAGCAGCGACTACAAGGATAACCACGGCGAGTACCCCAAGAGCAAGGA
CATCCTGGGCTACACCAACGTGCACGGCTACGCCTACCACACCATCAAGACAAAGGCCTACAGACTGAACT
CTGGAAATCTGAGCCAGACCATCAAGAGAGCCACAGACAGGTTCAAGGCCTACCAGAAGGAGATCCTGCGC
GGCGACATGTCTATCCCCAGCTACAAGAGGGACATCCCCCTGGACCTGATCAAGGAGAACATCTCCGTGAA
CAGGATGAATCACGGCGACTACATCGCCAGCCTGTCTCTGCTGAGCAACCCCGCCAAGCAGGAGATGAACG
TGAAGAGAAAGATCTCCGTGATCATCATCGTGAGGGGCGCCGGCAAGACCATCATGGACAGAATCCTGTCC
GGCGAGTACCAGGTGAGCGCCAGCCAGATTATCCACGACGACCGGAAGAACAAGTGGTACCTGAACATCAG
CTACGACTTCGAGCCACAGACCAGAGTGCTGGACCTGAACAAGATCATGGGCATTGACCTGGGCGTGGCC
GTGGCCGTGTACATGGCCTTCCAGCACACCCCCGCCAGGTACAAGCTGGAGGGCGGCGAGATTGAGAACT
TCAGGAGGCAGGTGGAGAGCCGGCGCATCTCCATGCTGAGACAGGGCAAGTACGCCGGCGGCGCCAGGG
GCGGCCACGGCAGAGACAAGAGAATCAAGCCCATTGAGCAGCTGAGGGATAAGATCGCCAATTTCAGAGAC
ACCACCAATCACCGGTACAGCAGATACATCGTGGACATGGCCATCAAGGAGGGCTGCGGCACAATCCAGAT
GGAGGATCTGACAAACATCAGAGACATCGGCAGCAGATTCCTGCAGAACTGGACCTACTACGACCTGCAGC
AGAAGATCATCTACAAGGCCGAGGAGGCCGGCATCAAAGTGATCAAGATCGACCCCCAGTACACCAGCCAG
AGATGCTCCGAGTGCGGCAACATCGACTCCGGCAACAGAATCGGCCAGGCCATCTTTAAGTGCCGGGCCT
GCGGCTACGAGGCCAACGCCGACTACAACGCCGCCCGGAATATCGCCATCCCCAACATCGACAAGATCATC
GCCGAGAGCATTAAGAAAAGGCCGGCGGCCACGAAAAAGGCCGGCCAGGCAAAAAAGAAAAAGGGATCCT
ACCCATACGATGTTCCAGATTACGCTTATCCCTACGACGTGCCTGATTATGCATACCCATACGATGTCCCCGAC
TATGCCCTCGAGAGCACCGGTGGCAGCGGAGCTACTAACTTCAGCCTGCTGAAGCAGGCTGGAGACGTGG
AGGAGAACCCTGGACCTGCCGGTATGGTGAGCAAGGGCGAGGAGGATAACATGGCCATCATCAAGGAGTTC
ATGCGCTTCAAGGTGCACATGGAGGGCTCCGTGAACGGCCACGAGTTCGAGATCGAGGGCGAGGGCGAG
GGCCGCCCCTACGAGGGCACCCAGACCGCCAAGCTGAAGGTGACCAAGGGTGGCCCCCTGCCCTTCGCC
TGGGACATCCTGTCCCCTCAGTTCATGTACGGCTCCAAGGCCTACGTGAAGCACCCCGCCGACATCCCCGA
CTACTTGAAGCTGTCCTTCCCCGAGGGCTTCAAGTGGGAGCGCGTGATGAACTTCGAGGACGGCGGCGTG
GTGACCGTGACCCAGGACTCCTCCCTGCAGGACGGCGAGTTCATCTACAAGGTGAAGCTGCGCGGCACCA
ACTTCCCCTCCGACGGCCCCGTAATGCAGAAGAAGACCATGGGCTGGGAGGCCTCCTCCGAGCGGATGTA
CCCCGAGGACGGCGCCCTGAAGGGCGAGATCAAGCAGAGGCTGAAGCTGAAGGACGGCGGCCACTACGA

CGCTGAGGTCAAGACCACCTACAAGGCCAAGAAGCCCGTGCAGCTGCCCGGCGCCTACAACGTCAACATC
AAGTTGGACATCACCTCCCACAACGAGGACTACACCATCGTGGAACAGTACGAACGCGCCGAGGGCCGCC
ACTCCACCGGCGGCATGGACGAGCTGTACAAGTAA

- Expression of the Un1Cas12f1-WT nuclease：

Orange: SV40 NLS, blue: Un1Cas12f1-WT, violet: nucleoplasmin NLS, green: 3XHA, yellow: P2A, Red: mCherry
CCAAAGAAGAAGCGGAAGGTCGGTATCCACGGAGTCCCAGCAGCCATGGCCAAGAACACAATTACAAAGAC
ACTGAAGCTGAGGATCGTGAGACCATACAACAGCGCTGAGGTCGAGAAGATTGTGGCTGATGAAAAGAACA
ACAGGGAAAAGATCGCCCTCGAGAAGAACAAGGATAAGGTGAAGGAGGCCTGCTCTAAGCACCTGAAAGTG
GCCGCCTACTGCACCACACAGGTGGAGAGGAACGCCTGTCTGTTTTGTAAAGCTCGGAAGCTGGATGATAA
GTTTTACCAGAAGCTGCGGGGCCAGTTCCCCGATGCCGTCTTTTGGCAGGAGATTAGCGAGATCTTCAGAC
AGCTGCAGAAGCAGGCCGCCGAGATCTACAACCAGAGCCTGATCGAGCTCTACTACGAGATCTTCATCAAG
GGCAAGGGCATTGCCAACGCCTCCTCCGTGGAGCACTACCTGAGCGACGTGTGCTACACAAGAGCCGCCG
AGCTCTTTAAGAACGCCGCTATCGCTTCCGGGCTGAGGAGCAAGATTAAGAGTAACTTCCGGCTCAAGGAG
CTGAAGAACATGAAGAGCGGCCTGCCCACTACAAAGAGCGACAACTTCCCAATTCCACTGGTGAAGCAGAA
GGGGGGCCAGTACACAGGGTTCGAGATTTCCAACCACAACAGCGACTTTATTATTAAGATCCCCTTTGGCAG
GTGGCAGGTCAAGAAGGAGATTGACAAGTACAGGCCCTGGGAGAAGTTTGATTTCGAGCAGGTGCAGAAGA
GCCCCAAGCCTATTTCCCTGCTGCTGTCCACACAGCGGCGGAAGAGGAACAAGGGGTGGTCTAAGGATGA
GGGGACCGAGGCCGAGATTAAGAAAGTGATGAACGGCGACTACCAGACAAGCTACATCGAGGTCAAGCGG
GGCAGTAAGATTTGCGAGAAGAGCGCCTGGATGCTGAACCTGAGCATTGACGTGCCAAAGATTGATAAGGG
CGTGGACCCCAGCATCATCGGAGGGATCGATGTGGGGGTCAAGAGCCCCCTCGTGTGCGCCATCAACAAC
GCCTTCAGCAGGTACAGCATCTCCGATAACGACCTGTTCCACTTTAACAAGAAGATGTTCGCCCGGCGGAG
GATTTTGCTCAAGAAGAACCGGCACAAGCGGGCCGGACACGGGGCCAAGAACAAGCTCAAGCCCATCACT
ATCCTGACCGAGAAGAGCGAGAGGTTCAGGAAGAAGCTCATCGAGAGATGGGCCTGCGAGATCGCCGATTT
CTTTATTAAGAACAAGGTCGGAACAGTGCAGATGGAGAACCTCGAGAGCATGAAGAGGAAGGAGGATTCCTA
CTTCAACATTCGGCTGAGGGGGTTCTGGCCCTACGCTGAGATGCAGAACAAGATTGAGTTTAAGCTGAAGC
AGTACGGGATTGAGATCCGGAAGGTGGCCCCCAACAACACCAGCAAGACCTGCAGCAAGTGCGGGCACCT
CAACAACTACTTCAACTTCGAGTACCGGAAGAAGAACAAGTTCCCACACTTCAAGTGCGAGAAGTGCAACTT
TAAGGAGAACGCCGATTACAACGCCGCCCTGAACATCAGCAACCCTAAGCTGAAGAGCACTAAGGAGGAGC
CCAAAAGGCCGGCGGCCACGAAAAAGGCCGGCCAGGCAAAAAAGAAAAAGGGATCCTACCCATACGATGTT
CCAGATTACGCTTATCCCTACGACGTGCCTGATTATGCATACCCATACGATGTCCCCGACTATGCCCTCGAGA
GCACCGGTGGCAGCGGAGCTACTAACTTCAGCCTGCTGAAGCAGGCTGGAGACGTGGAGGAGAACCCTG
GACCTGCCGGTATGGTGAGCAAGGGCGAGGAGGATAACATGGCCATCATCAAGGAGTTCATGCGCTTCAAG
GTGCACATGGAGGGCTCCGTGAACGGCCACGAGTTCGAGATCGAGGGCGAGGGCGAGGGCCGCCCCTAC
GAGGGCACCCAGACCGCCAAGCTGAAGGTGACCAAGGGTGGCCCCCTGCCCTTCGCCTGGGACATCCTG
TCCCCTCAGTTCATGTACGGCTCCAAGGCCTACGTGAAGCACCCCGCCGACATCCCCGACTACTTGAAGCT
GTCCTTCCCCGAGGGCTTCAAGTGGGAGCGCGTGATGAACTTCGAGGACGGCGGCGTGGTGACCGTGAC
CCAGGACTCCTCCCTGCAGGACGGCGAGTTCATCTACAAGGTGAAGCTGCGCGGCACCAACTTCCCCTCC
GACGGCCCCGTAATGCAGAAGAAGACCATGGGCTGGGAGGCCTCCTCCGAGCGGATGTACCCCGAGGACG
GCGCCCTGAAGGGCGAGATCAAGCAGAGGCTGAAGCTGAAGGACGGCGGCCACTACGACGCTGAGGTCA
AGACCACCTACAAGGCCAAGAAGCCCGTGCAGCTGCCCGGCGCCTACAACGTCAACATCAAGTTGGACATC
ACCTCCCACAACGAGGACTACACCATCGTGGAACAGTACGAACGCGCCGAGGGCCGCCACTCCACCGGCG
GCATGGACGAGCTGTACAAGTAA

- Expression of the Un1Cas12f1-V3.1 nuclease：

Orange: SV40 NLS, blue: Un1Cas12f1-V3.1(D143R/T147R/E151A), violet: nucleoplasmin NLS, green: 3XHA, yellow: P2A, Red: mCherry
CCAAAGAAGAAGCGGAAGGTCGGTATCCACGGAGTCCCAGCAGCCATGGCCAAGAACACAATTACAAAGAC
ACTGAAGCTGAGGATCGTGAGACCATACAACAGCGCTGAGGTCGAGAAGATTGTGGCTGATGAAAAGAACA

ACAGGGAAAAGATCGCCCTCGAGAAGAACAAGGATAAGGTGAAGGAGGCCTGCTCTAAGCACCTGAAAGTG
GCCGCCTACTGCACCACACAGGTGGAGAGGAACGCCTGTCTGTTTTGTAAAGCTCGGAAGCTGGATGATAA
GTTTTACCAGAAGCTGCGGGGCCAGTTCCCCGATGCCGTCTTTTGGCAGGAGATTAGCGAGATCTTCAGAC
AGCTGCAGAAGCAGGCCGCCGAGATCTACAACCAGAGCCTGATCGAGCTCTACTACGAGATCTTCATCAAG
GGCAAGGGCATTGCCAACGCCTCCTCCGTGGAGCACTACCTGAGCAGAGTGTGCTACAGAAGAGCCGCCG
CTCTCTTTAAGAACGCCGCTATCGCTTCCGGGCTGAGGAGCAAGATTAAGAGTAACTTCCGGCTCAAGGAGC
TGAAGAACATGAAGAGCGGCCTGCCCACTACAAAGAGCGACAACTTCCCAATTCCACTGGTGAAGCAGAAG
GGGGGCCAGTACACAGGGTTCGAGATTTCCAACCACAACAGCGACTTTATTATTAAGATCCCCTTTGGCAGG
TGGCAGGTCAAGAAGGAGATTGACAAGTACAGGCCCTGGGAGAAGTTTGATTTCGAGCAGGTGCAGAAGAG
CCCCAAGCCTATTTCCCTGCTGCTGTCCACACAGCGGCGGAAGAGGAACAAGGGGTGGTCTAAGGATGAG
GGGACCGAGGCCGAGATTAAGAAAGTGATGAACGGCGACTACCAGACAAGCTACATCGAGGTCAAGCGGG
GCAGTAAGATTTGCGAGAAGAGCGCCTGGATGCTGAACCTGAGCATTGACGTGCCAAAGATTGATAAGGGC
GTGGACCCCAGCATCATCGGAGGGATCGATGTGGGGGTCAAGAGCCCCCTCGTGTGCGCCATCAACAACG
CCTTCAGCAGGTACAGCATCTCCGATAACGACCTGTTCCACTTTAACAAGAAGATGTTCGCCCGGCGGAGGA
TTTTGCTCAAGAAGAACCGGCACAAGCGGGCCGGACACGGGGCCAAGAACAAGCTCAAGCCCATCACTATC
CTGACCGAGAAGAGCGAGAGGTTCAGGAAGAAGCTCATCGAGAGATGGGCCTGCGAGATCGCCGATTTCTT
TATTAAGAACAAGGTCGGAACAGTGCAGATGGAGAACCTCGAGAGCATGAAGAGGAAGGAGGATTCCTACTT
CAACATTCGGCTGAGGGGGTTCTGGCCCTACGCTGAGATGCAGAACAAGATTGAGTTTAAGCTGAAGCAGT
ACGGGATTGAGATCCGGAAGGTGGCCCCCAACAACACCAGCAAGACCTGCAGCAAGTGCGGGCACCTCAA
CAACTACTTCAACTTCGAGTACCGGAAGAAGAACAAGTTCCCACACTTCAAGTGCGAGAAGTGCAACTTTAA
GGAGAACGCCGATTACAACGCCGCCCTGAACATCAGCAACCCTAAGCTGAAGAGCACTAAGGAGGAGCCCA
AAAGGCCGGCGGCCACGAAAAAGGCCGGCCAGGCAAAAAAGAAAAAGGGATCCTACCCATACGATGTTCCA
GATTACGCTTATCCCTACGACGTGCCTGATTATGCATACCCATACGATGTCCCCGACTATGCCCTCGAGAGCA
CCGGTGGCAGCGGAGCTACTAACTTCAGCCTGCTGAAGCAGGCTGGAGACGTGGAGGAGAACCCTGGACC
TGCCGGTATGGTGAGCAAGGGCGAGGAGGATAACATGGCCATCATCAAGGAGTTCATGCGCTTCAAGGTGC
ACATGGAGGGCTCCGTGAACGGCCACGAGTTCGAGATCGAGGGCGAGGGCGAGGGCCGCCCCTACGAGG
GCACCCAGACCGCCAAGCTGAAGGTGACCAAGGGTGGCCCCCTGCCCTTCGCCTGGGACATCCTGTCCCC
TCAGTTCATGTACGGCTCCAAGGCCTACGTGAAGCACCCCGCCGACATCCCCGACTACTTGAAGCTGTCCT
TCCCCGAGGGCTTCAAGTGGGAGCGCGTGATGAACTTCGAGGACGGCGGCGTGGTGACCGTGACCCAGG
ACTCCTCCCTGCAGGACGGCGAGTTCATCTACAAGGTGAAGCTGCGCGGCACCAACTTCCCCTCCGACGG
CCCCGTAATGCAGAAGAAGACCATGGGCTGGGAGGCCTCCTCCGAGCGGATGTACCCCGAGGACGGCGCC
CTGAAGGGCGAGATCAAGCAGAGGCTGAAGCTGAAGGACGGCGGCCACTACGACGCTGAGGTCAAGACC
ACCTACAAGGCCAAGAAGCCCGTGCAGCTGCCCGGCGCCTACAACGTCAACATCAAGTTGGACATCACCTC
CCACAACGAGGACTACACCATCGTGGAACAGTACGAACGCGCCGAGGGCCGCCACTCCACCGGCGGCATG
GACGAGCTGTACAAGTAA

- Expression of the AsCas12a nuclease：

Blue: AsCas12a, violet: nucleoplasmin NLS, green: 3XHA, yellow: P2A, Red: mCherry
ATGACACAGTTCGAGGGCTTTACCAACCTGTATCAGGTGAGCAAGACACTGCGGTTTGAGCTGATCCCACA
GGGCAAGACCCTGAAGCACATCCAGGAGCAGGGCTTCATCGAGGAGGACAAGGCCCGCAATGATCACTAC
AAGGAGCTGAAGCCCATCATCGATCGGATCTACAAGACCTATGCCGACCAGTGCCTGCAGCTGGTGCAGCT
GGATTGGGAGAACCTGAGCGCCGCCATCGACTCCTATAGAAAGGAGAAAACCGAGGAGACAAGGAACGCC
CTGATCGAGGAGCAGGCCACATATCGCAATGCCATCCACGACTACTTCATCGGCCGGACAGACAACCTGAC
CGATGCCATCAATAAGAGACACGCCGAGATCTACAAGGGCCTGTTCAAGGCCGAGCTGTTTAATGGCAAGGT
GCTGAAGCAGCTGGGCACCGTGACCACAACCGAGCACGAGAACGCCCTGCTGCGGAGCTTCGACAAGTTT
ACAACCTACTTCTCCGGCTTTTATGAGAACAGGAAGAACGTGTTCAGCGCCGAGGATATCAGCACAGCCATC
CCACACCGCATCGTGCAGGACAACTTCCCCAAGTTTAAGGAGAATTGTCACATCTTCACACGCCTGATCACC
GCCGTGCCCAGCCTGCGGGAGCACTTTGAGAACGTGAAGAAGGCCATCGGCATCTTCGTGAGCACCTCCA
TCGAGGAGGTGTTTTCCTTCCCTTTTTATAACCAGCTGCTGACACAGACCCAGATCGACCTGTATAACCAGCT

GCTGGGAGGAATCTCTCGGGAGGCAGGCACCGAGAAGATCAAGGGCCTGAACGAGGTGCTGAATCTGGCC
ATCCAGAAGAATGATGAGACAGCCCACATCATCGCCTCCCTGCCACACAGATTCATCCCCCTGTTTAAGCAG
ATCCTGTCCGATAGGAACACCCTGTCTTTCATCCTGGAGGAGTTTAAGAGCGACGAGGAAGTGATCCAGTCC
TTCTGCAAGTACAAGACACTGCTGAGAAACGAGAACGTGCTGGAGACAGCCGAGGCCCTGTTTAACGAGCT
GAACAGCATCGACCTGACACACATCTTCATCAGCCACAAGAAGCTGGAGACAATCAGCAGCGCCCTGTGCG
ACCACTGGGATACACTGAGGAATGCCCTGTATGAGCGGAGAATCTCCGAGCTGACAGGCAAGATCACCAAG
TCTGCCAAGGAGAAGGTGCAGCGCAGCCTGAAGCACGAGGATATCAACCTGCAGGAGATCATCTCTGCCGC
AGGCAAGGAGCTGAGCGAGGCCTTCAAGCAGAAAACCAGCGAGATCCTGTCCCACGCACACGCCGCCCTG
GATCAGCCACTGCCTACAACCCTGAAGAAGCAGGAGGAGAAGGAGATCCTGAAGTCTCAGCTGGACAGCCT
GCTGGGCCTGTACCACCTGCTGGACTGGTTTGCCGTGGATGAGTCCAACGAGGTGGACCCCGAGTTCTCT
GCCCGGCTGACCGGCATCAAGCTGGAGATGGAGCCTTCTCTGAGCTTCTACAACAAGGCCAGAAATTATGC
CACCAAGAAGCCCTACTCCGTGGAGAAGTTCAAGCTGAACTTTCAGATGCCTACACTGGCCTCTGGCTGGG
ACGTGAATAAGGAGAAGAACAATGGCGCCATCCTGTTTGTGAAGAACGGCCTGTACTATCTGGGCATCATGC
CAAAGCAGAAGGGCAGGTATAAGGCCCTGAGCTTCGAGCCCACAGAGAAAACCAGCGAGGGCTTTGATAAG
ATGTACTATGACTACTTCCCTGATGCCGCCAAGATGATCCCAAAGTGCAGCACCCAGCTGAAGGCCGTGACA
GCCCACTTTCAGACCCACACAACCCCCATCCTGCTGTCCAACAATTTCATCGAGCCTCTGGAGATCACAAAG
GAGATCTACGACCTGAACAATCCTGAGAAGGAGCCAAAGAAGTTTCAGACAGCCTACGCCAAGAAAACCGG
CGACCAGAAGGGCTACAGAGAGGCCCTGTGCAAGTGGATCGACTTCACAAGGGATTTTCTGTCCAAGTATA
CCAAGACAACCTCTATCGATCTGTCTAGCCTGCGGCCATCCTCTCAGTATAAGGACCTGGGCGAGTACTATG
CCGAGCTGAATCCCCTGCTGTACCACATCAGCTTCCAGAGAATCGCCGAGAAGGAGATCATGGATGCCGTG
GAGACAGGCAAGCTGTACCTGTTCCAGATCTATAACAAGGACTTTGCCAAGGGCCACCACGGCAAGCCTAAT
CTGCACACACTGTATTGGACCGGCCTGTTTTCTCCAGAGAACCTGGCCAAGACAAGCATCAAGCTGAATGG
CCAGGCCGAGCTGTTCTACCGCCCTAAGTCCAGGATGAAGAGGATGGCACACCGGCTGGGAGAGAAGATG
CTGAACAAGAAGCTGAAGGATCAGAAAACCCCAATCCCCGACACCCTGTACCAGGAGCTGTACGACTATGT
GAATCACAGACTGTCCCACGACCTGTCTGATGAGGCCAGGGCCCTGCTGCCCAACGTGATCACCAAGGAG
GTGTCTCACGAGATCATCAAGGATAGGCGCTTTACCAGCGACAAGTTCTTTTTCCACGTGCCTATCACACTGA
ACTATCAGGCCGCCAATTCCCCATCTAAGTTCAACCAGAGGGTGAATGCCTACCTGAAGGAGCACCCCGAG
ACACCTATCATCGGCATCGATCGGGGCGAGAGAAACCTGATCTATATCACAGTGATCGACTCCACCGGCAAG
ATCCTGGAGCAGCGGAGCCTGAACACCATCCAGCAGTTTGATTACCAGAAGAAGCTGGACAACAGGGAGAA
GGAGAGGGTGGCAGCAAGGCAGGCCTGGTCTGTGGTGGGCACAATCAAGGATCTGAAGCAGGGCTATCTG
AGCCAGGTCATCCACGAGATCGTGGACCTGATGATCCACTACCAGGCCGTGGTGGTGCTGGAGAACCTGAA
TTTCGGCTTTAAGAGCAAGAGGACCGGCATCGCCGAGAAGGCCGTGTACCAGCAGTTCGAGAAGATGCTGA
TCGATAAGCTGAATTGCCTGGTGCTGAAGGACTATCCAGCAGAGAAAGTGGGAGGCGTGCTGAACCCATAC
CAGCTGACAGACCAGTTCACCTCCTTTGCCAAGATGGGCACCCAGTCTGGCTTCCTGTTTTACGTGCCTGC
CCCATATACATCTAAGATCGATCCCCTGACCGGCTTCGTGGACCCCTTCGTGTGGAAAACCATCAAGAATCAC
GAGAGCCGCAAGCACTTCCTGGAGGGCTTCGACTTTCTGCACTACGACGTGAAAACCGGCGACTTCATCCT
GCACTTTAAGATGAACAGAAATCTGTCCTTCCAGAGGGGCCTGCCCGGCTTTATGCCTGCATGGGATATCGT
GTTCGAGAAGAACGAGACACAGTTTGACGCCAAGGGCACCCCTTTCATCGCCGGCAAGAGAATCGTGCCA
GTGATCGAGAATCACAGATTCACCGGCAGATACCGGGACCTGTATCCTGCCAACGAGCTGATCGCCCTGCT
GGAGGAGAAGGGCATCGTGTTCAGGGATGGCTCCAACATCCTGCCAAAGCTGCTGGAGAATGACGATTCTC
ACGCCATCGACACCATGGTGGCCCTGATCCGCAGCGTGCTGCAGATGCGGAACTCCAATGCCGCCACAGG
CGAGGACTATATCAACAGCCCCGTGCGCGATCTGAATGGCGTGTGCTTCGACTCCCGGTTTCAGAACCCAG
AGTGGCCCATGGACGCCGATGCCAATGGCGCCTACCACATCGCCCTGAAGGGCCAGCTGCTGCTGAATCA
CCTGAAGGAGAGCAAGGATCTGAAGCTGCAGAACGGCATCTCCAATCAGGACTGGCTGGCCTACATCCAGG
AGCTGCGCAACAAAAGGCCGGCGGCCACGAAAAAGGCCGGCCAGGCAAAAAAGAAAAAGGGATCCTACCC
ATACGATGTTCCAGATTACGCTTATCCCTACGACGTGCCTGATTATGCATACCCATACGATGTCCCCGACTATG
CCCTCGAGAGCACCGGTGGCAGCGGAGCTACTAACTTCAGCCTGCTGAAGCAGGCTGGAGACGTGGAGGA
GAACCCTGGACCTGCCGGTATGGTGAGCAAGGGCGAGGAGGATAACATGGCCATCATCAAGGAGTTCATGC
GCTTCAAGGTGCACATGGAGGGCTCCGTGAACGGCCACGAGTTCGAGATCGAGGGCGAGGGCGAGGGCC

GCCCCTACGAGGGCACCCAGACCGCCAAGCTGAAGGTGACCAAGGGTGGCCCCCTGCCCTTCGCCTGGG
ACATCCTGTCCCCTCAGTTCATGTACGGCTCCAAGGCCTACGTGAAGCACCCCGCCGACATCCCCGACTAC
TTGAAGCTGTCCTTCCCCGAGGGCTTCAAGTGGGAGCGCGTGATGAACTTCGAGGACGGCGGCGTGGTGA
CCGTGACCCAGGACTCCTCCCTGCAGGACGGCGAGTTCATCTACAAGGTGAAGCTGCGCGGCACCAACTT
CCCCTCCGACGGCCCCGTAATGCAGAAGAAGACCATGGGCTGGGAGGCCTCCTCCGAGCGGATGTACCCC
GAGGACGGCGCCCTGAAGGGCGAGATCAAGCAGAGGCTGAAGCTGAAGGACGGCGGCCACTACGACGCT
GAGGTCAAGACCACCTACAAGGCCAAGAAGCCCGTGCAGCTGCCCGGCGCCTACAACGTCAACATCAAGTT
GGACATCACCTCCCACAACGAGGACTACACCATCGTGGAACAGTACGAACGCGCCGAGGGCCGCCACTCC
ACCGGCGGCATGGACGAGCTGTACAAGTAA

- Expression of the LbCas12a nuclease：

Blue: LbCas12a, violet: nucleoplasmin NLS, green: 3XHA, yellow: P2A, Red: mCherry

ATGAGCAAGCTGGAGAAGTTTACAAACTGCTACTCCCTGTCTAAGACCCTGAGGTTCAAGGCCATCCCTGTG
GGCAAGACCCAGGAGAACATCGACAATAAGCGGCTGCTGGTGGAGGACGAGAAGAGAGCCGAGGATTATA
AGGGCGTGAAGAAGCTGCTGGATCGCTACTATCTGTCTTTTATCAACGACGTGCTGCACAGCATCAAGCTGA
AGAATCTGAACAATTACATCAGCCTGTTCCGGAAGAAAACCAGAACCGAGAAGGAGAATAAGGAGCTGGAG
AACCTGGAGATCAATCTGCGGAAGGAGATCGCCAAGGCCTTCAAGGGCAACGAGGGCTACAAGTCCCTGTT
TAAGAAGGATATCATCGAGACAATCCTGCCAGAGTTCCTGGACGATAAGGACGAGATCGCCCTGGTGAACAG
CTTCAATGGCTTTACCACAGCCTTCACCGGCTTCTTTGATAACAGAGAGAATATGTTTTCCGAGGAGGCCAAG
AGCACATCCATCGCCTTCAGGTGTATCAACGAGAATCTGACCCGCTACATCTCTAATATGGACATCTTCGAGA
AGGTGGACGCCATCTTTGATAAGCACGAGGTGCAGGAGATCAAGGAGAAGATCCTGAACAGCGACTATGAT
GTGGAGGATTTCTTTGAGGGCGAGTTCTTTAACTTTGTGCTGACACAGGAGGGCATCGACGTGTATAACGCC
ATCATCGGCGGCTTCGTGACCGAGAGCGGCGAGAAGATCAAGGGCCTGAACGAGTACATCAACCTGTATAA
TCAGAAAACCAAGCAGAAGCTGCCTAAGTTTAAGCCACTGTATAAGCAGGTGCTGAGCGATCGGGAGTCTCT
GAGCTTCTACGGCGAGGGCTATACATCCGATGAGGAGGTGCTGGAGGTGTTTAGAAACACCCTGAACAAGA
ACAGCGAGATCTTCAGCTCCATCAAGAAGCTGGAGAAGCTGTTCAAGAATTTTGACGAGTACTCTAGCGCCG
GCATCTTTGTGAAGAACGGCCCCGCCATCAGCACAATCTCCAAGGATATCTTCGGCGAGTGGAACGTGATCC
GGGACAAGTGGAATGCCGAGTATGACGATATCCACCTGAAGAAGAAGGCCGTGGTGACCGAGAAGTACGAG
GACGATCGGAGAAAGTCCTTCAAGAAGATCGGCTCCTTTTCTCTGGAGCAGCTGCAGGAGTACGCCGACGC
CGATCTGTCTGTGGTGGAGAAGCTGAAGGAGATCATCATCCAGAAGGTGGATGAGATCTACAAGGTGTATGG
CTCCTCTGAGAAGCTGTTCGACGCCGATTTTGTGCTGGAGAAGAGCCTGAAGAAGAACGACGCCGTGGTG
GCCATCATGAAGGACCTGCTGGATTCTGTGAAGAGCTTCGAGAATTACATCAAGGCCTTCTTTGGCGAGGGC
AAGGAGACAAACAGGGACGAGTCCTTCTATGGCGATTTTGTGCTGGCCTACGACATCCTGCTGAAGGTGGA
CCACATCTACGATGCCATCCGCAATTATGTGACCCAGAAGCCCTACTCTAAGGATAAGTTCAAGCTGTATTTTC
AGAACCCTCAGTTCATGGGCGGCTGGGACAAGGATAAGGAGACAGACTATCGGGCCACCATCCTGAGATAC
GGCTCCAAGTACTATCTGGCCATCATGGATAAGAAGTACGCCAAGTGCCTGCAGAAGATCGACAAGGACGAT
GTGAACGGCAATTACGAGAAGATCAACTATAAGCTGCTGCCCGGCCCTAATAAGATGCTGCCAAAGGTGTTC
TTTTCTAAGAAGTGGATGGCCTACTATAACCCCAGCGAGGACATCCAGAAGATCTACAAGAATGGCACATTCA
AGAAGGGCGATATGTTTAACCTGAATGACTGTCACAAGCTGATCGACTTCTTTAAGGATAGCATCTCCCGGTA
TCCAAAGTGGTCCAATGCCTACGATTTCAACTTTTCTGAGACAGAGAAGTATAAGGACATCGCCGGCTTTTAC
AGAGAGGTGGAGGAGCAGGGCTATAAGGTGAGCTTCGAGTCTGCCAGCAAGAAGGAGGTGGATAAGCTGG
TGGAGGAGGGCAAGCTGTATATGTTCCAGATCTATAACAAGGACTTTTCCGATAAGTCTCACGGCACACCCAA
TCTGCACACCATGTACTTCAAGCTGCTGTTTGACGAGAACAATCACGGACAGATCAGGCTGAGCGGAGGAG
CAGAGCTGTTCATGAGGCGCGCCTCCCTGAAGAAGGAGGAGCTGGTGGTGCACCCAGCCAACTCCCCTAT
CGCCAACAAGAATCCAGATAATCCCAAGAAAACCACAACCCTGTCCTACGACGTGTATAAGGATAAGAGGTTT
TCTGAGGACCAGTACGAGCTGCACATCCCAATCGCCATCAATAAGTGCCCCAAGAACATCTTCAAGATCAATA
CAGAGGTGCGCGTGCTGCTGAAGCACGACGATAACCCCTATGTGATCGGCATCGATAGGGGCGAGCGCAAT
CTGCTGTATATCGTGGTGGTGGACGGCAAGGGCAACATCGTGGAGCAGTATTCCCTGAACGAGATCATCAAC
AACTTCAACGGCATCAGGATCAAGACAGATTACCACTCTCTGCTGGACAAGAAGGAGAAGGAGAGGTTCGA

GGCCCGCCAGAACTGGACCTCCATCGAGAATATCAAGGAGCTGAAGGCCGGCTATATCTCTCAGGTGGTGC
ACAAGATCTGCGAGCTGGTGGAGAAGTACGATGCCGTGATCGCCCTGGAGGACCTGAACTCTGGCTTTAAG
AATAGCCGCGTGAAGGTGGAGAAGCAGGTGTATCAGAAGTTCGAGAAGATGCTGATCGATAAGCTGAACTAC
ATGGTGGACAAGAAGTCTAATCCTTGTGCAACAGGCGGCGCCCTGAAGGGCTATCAGATCACCAATAAGTTC
GAGAGCTTTAAGTCCATGTCTACCCAGAACGGCTTCATCTTTTACATCCCTGCCTGGCTGACATCCAAGATCG
ATCCATCTACCGGCTTTGTGAACCTGCTGAAAACCAAGTATACCAGCATCGCCGATTCCAAGAAGTTCATCAG
CTCCTTTGACAGGATCATGTACGTGCCCGAGGAGGATCTGTTCGAGTTTGCCCTGGACTATAAGAACTTCTC
TCGCACAGACGCCGATTACATCAAGAAGTGGAAGCTGTACTCCTACGGCAACCGGATCAGAATCTTCCGGAA
TCCTAAGAAGAACAACGTGTTCGACTGGGAGGAGGTGTGCCTGACCAGCGCCTATAAGGAGCTGTTCAACA
AGTACGGCATCAATTATCAGCAGGGCGATATCAGAGCCCTGCTGTGCGAGCAGTCCGACAAGGCCTTCTACT
CTAGCTTTATGGCCCTGATGAGCCTGATGCTGCAGATGCGGAACAGCATCACAGGCCGCACCGACGTGGAT
TTTCTGATCAGCCCTGTGAAGAACTCCGACGGCATCTTCTACGATAGCCGGAACTATGAGGCCCAGGAGAAT
GCCATCCTGCCAAAGAACGCCGACGCCAATGGCGCCTATAACATCGCCAGAAAGGTGCTGTGGGCCATCGG
CCAGTTCAAGAAGGCCGAGGACGAGAAGCTGGATAAGGTGAAGATCGCCATCTCTAACAAGGAGTGGCTGG
AGTACGCCCAGACCAGCGTGAAGCACAAAAGGCCGGCGGCCACGAAAAAGGCCGGCCAGGCAAAAAAGA
AAAAGGGATCCTACCCATACGATGTTCCAGATTACGCTTATCCCTACGACGTGCCTGATTATGCATACCCATAC
GATGTCCCCGACTATGCCCTCGAGAGCACCGGTGGCAGCGGAGCTACTAACTTCAGCCTGCTGAAGCAGG
CTGGAGACGTGGAGGAGAACCCTGGACCTGCCGGTATGGTGAGCAAGGGCGAGGAGGATAACATGGCCAT
CATCAAGGAGTTCATGCGCTTCAAGGTGCACATGGAGGGCTCCGTGAACGGCCACGAGTTCGAGATCGAG
GGCGAGGGCGAGGGCCGCCCCTACGAGGGCACCCAGACCGCCAAGCTGAAGGTGACCAAGGGTGGCCC
CCTGCCCTTCGCCTGGGACATCCTGTCCCCTCAGTTCATGTACGGCTCCAAGGCCTACGTGAAGCACCCCG
CCGACATCCCCGACTACTTGAAGCTGTCCTTCCCCGAGGGCTTCAAGTGGGAGCGCGTGATGAACTTCGAG
GACGGCGGCGTGGTGACCGTGACCCAGGACTCCTCCCTGCAGGACGGCGAGTTCATCTACAAGGTGAAGC
TGCGCGGCACCAACTTCCCCTCCGACGGCCCCGTAATGCAGAAGAAGACCATGGGCTGGGAGGCCTCCTC
CGAGCGGATGTACCCCGAGGACGGCGCCCTGAAGGGCGAGATCAAGCAGAGGCTGAAGCTGAAGGACGG
CGGCCACTACGACGCTGAGGTCAAGACCACCTACAAGGCCAAGAAGCCCGTGCAGCTGCCCGGCGCCTAC
AACGTCAACATCAAGTTGGACATCACCTCCCACAACGAGGACTACACCATCGTGGAACAGTACGAACGCGC
CGAGGGCCGCCACTCCACCGGCGGCATGGACGAGCTGTACAAGTAA

- Expression of the SpCas9 nuclease：

Green: 3XFlag, Orange: SV40 NLS, blue: SpCas9, violet: nucleoplasmin NLS, yellow: P2A, Red: mCherry
GACTACAAAGACCATGACGGTGATTATAAAGATCATGACATCGATTACAAGGATGACGATGACAAGATGGCCC
CCAAGAAGAAGAGGAAGGTGGGCATTCACCGCGGGGTACCCATGGACAAGAAGTACTCCATTGGGCTCGAT
ATCGGCACAAACAGCGTCGGCTGGGCCGTCATTACGGACGAGTACAAGGTGCCGAGCAAAAAATTCAAAGT
TCTGGGCAATACCGATCGCCACAGCATAAAGAAGAACCTCATTGGCGCCCTCCTGTTCGACTCCGGGGAGA
CGGCCGAAGCCACGCGGCTCAAAAGAACAGCACGGCGCAGATATACCCGCAGAAAGAATCGGATCTGCTAC
CTGCAGGAGATCTTTAGTAATGAGATGGCTAAGGTGGATGACTCTTTCTTCCATAGGCTGGAGGAGTCCTTTT
TGGTGGAGGAGGATAAAAAGCACGAGCGCCACCCAATCTTTGGCAATATCGTGGACGAGGTGGCGTACCAT
GAAAAGTACCCAACCATATATCATCTGAGGAAGAAGCTTGTAGACAGTACTGATAAGGCTGACTTGCGGTTGA
TCTATCTCGCGCTGGCGCATATGATCAAATTTCGGGGACACTTCCTCATCGAGGGGGACCTGAACCCAGACA
ACAGCGATGTCGACAAACTCTTTATCCAACTGGTTCAGACTTACAATCAGCTTTTCGAAGAGAACCCGATCAA
CGCATCCGGAGTTGACGCCAAAGCAATCCTGAGCGCTAGGCTGTCCAAATCCCGGCGGCTCGAAAACCTCA
TCGCACAGCTCCCTGGGGAGAAGAAGAACGGCCTGTTTGGTAATCTTATCGCCCTGTCACTCGGGCTGACC
CCCAACTTTAAATCTAACTTCGACCTGGCCGAAGATGCCAAGCTTCAACTGAGCAAAGACACCTACGATGAT
GATCTCGACAATCTGCTGGCCCAGATCGGCGACCAGTACGCAGACCTTTTTTTGGCGGCAAAGAACCTGTC
AGACGCCATTCTGCTGAGTGATATTCTGCGAGTGAACACGGAGATCACCAAAGCTCCGCTGAGCGCTAGTAT
GATCAAGCGCTATGATGAGCACCACCAAGACTTGACTTTGCTGAAGGCCCTTGTCAGACAGCAACTGCCTG
AGAAGTACAAGGAAATTTTCTTCGATCAGTCTAAAAATGGCTACGCCGGATACATTGACGGCGGAGCAAGCC
AGGAGGAATTTTACAAATTTATTAAGCCCATCTTGGAAAAAATGGACGGCACCGAGGAGCTGCTGGTAAAGCT

TAACAGAGAAGATCTGTTGCGCAAACAGCGCACTTTCGACAATGGAAGCATCCCCCACCAGATTCACCTGG
GCGAACTGCACGCTATCCTCAGGCGGCAAGAGGATTTCTACCCCTTTTTGAAAGATAACAGGGAAAAGATTG
AGAAAATCCTCACATTTCGGATACCCTACTATGTAGGCCCCCTCGCCCGGGGAAATTCCAGATTCGCGTGGA
TGACTCGCAAATCAGAAGAGACCATCACTCCCTGGAACTTCGAGGAAGTCGTGGATAAGGGGGCCTCTGCC
CAGTCCTTCATCGAAAGGATGACTAACTTTGATAAAAATCTGCCTAACGAAAAGGTGCTTCCTAAACACTCTC
TGCTGTACGAGTACTTCACAGTTTATAACGAGCTCACCAAGGTCAAATACGTCACAGAAGGGATGAGAAAGC
CAGCATTCCTGTCTGGAGAGCAGAAGAAAGCTATCGTGGACCTCCTCTTCAAGACGAACCGGAAAGTTACC
GTGAAACAGCTCAAAGAAGACTATTTCAAAAAGATTGAATGTTTCGACTCTGTTGAAATCAGCGGAGTGGAG
GATCGCTTCAACGCATCCCTGGGAACGTATCACGATCTCCTGAAAATCATTAAAGACAAGGACTTCCTGGAC
AATGAGGAGAACGAGGACATTCTTGAGGACATTGTCCTCACCCTTACGTTGTTTGAAGATAGGGAGATGATT
GAAGAACGCTTGAAAACTTACGCTCATCTCTTCGACGACAAAGTCATGAAACAGCTCAAGAGGCGCCGATAT
ACAGGATGGGGGCGGCTGTCAAGAAAACTGATCAATGGGATCCGAGACAAGCAGAGTGGAAAGACAATCCT
GGATTTTCTTAAGTCCGATGGATTTGCCAACCGGAACTTCATGCAGTTGATCCATGATGACTCTCTCACCTTTA
AGGAGGACATCCAGAAAGCACAAGTTTCTGGCCAGGGGGACAGTCTTCACGAGCACATCGCTAATCTTGCA
GGTAGCCCAGCTATCAAAAAGGGAATACTGCAGACCGTTAAGGTCGTGGATGAACTCGTCAAAGTAATGGGA
AGGCATAAGCCCGAGAATATCGTTATCGAGATGGCCCGAGAGAACCAAACTACCCAGAAGGGACAGAAGAA
CAGTAGGGAAAGGATGAAGAGGATTGAAGAGGGTATAAAAGAACTGGGGTCCCAAATCCTTAAGGAACACC
CAGTTGAAAACACCCAGCTTCAGAATGAGAAGCTCTACCTGTACTACCTGCAGAACGGCAGGGACATGTACG
TGGATCAGGAACTGGACATCAATCGGCTCTCCGACTACGACGTGGATCATATCGTGCCCCAGTCTTTTCTCA
AAGATGATTCTATTGATAATAAAGTGTTGACAAGATCCGATAAAAATAGAGGGAAGAGTGATAACGTCCCCTCA
GAAGAAGTTGTCAAGAAAATGAAAAATTATTGGCGGCAGCTGCTGAACGCCAAACTGATCACACAACGGAAG
TTCGATAATCTGACTAAGGCTGAACGAGGTGGCCTGTCTGAGTTGGATAAAGCCGGCTTCATCAAAAGGCAG
CTTGTTGAGACACGCCAGATCACCAAGCACGTGGCCCAAATTCTCGATTCACGCATGAACACCAAGTACGAT
GAAAATGACAAACTGATTCGAGAGGTGAAAGTTATTACTCTGAAGTCTAAGCTGGTCTCAGATTTCAGAAAGG
ACTTTCAGTTTTATAAGGTGAGAGAGATCAACAATTACCACCATGCGCATGATGCCTACCTGAATGCAGTGGT
AGGCACTGCACTTATCAAAAAATATCCCAAGCTTGAATCTGAATTTGTTTACGGAGACTATAAAGTGTACGATG
TTAGGAAAATGATCGCAAAGTCTGAGCAGGAAATAGGCAAGGCCACCGCTAAGTACTTCTTTTACAGCAATAT
TATGAATTTTTTCAAGACCGAGATTACACTGGCCAATGGAGAGATTCGGAAGCGACCACTTATCGAAACAAAC
GGAGAAACAGGAGAAATCGTGTGGGACAAGGGTAGGGATTTCGCGACAGTCCGGAAGGTCCTGTCCATGC
CGCAGGTGAACATCGTTAAAAAGACCGAAGTACAGACCGGAGGCTTCTCCAAGGAAAGTATCCTCCCGAAA
AGGAACAGCGACAAGCTGATCGCACGCAAAAAAGATTGGGACCCCAAGAAATACGGCGGATTCGATTCTCC
TACAGTCGCTTACAGTGTACTGGTTGTGGCCAAAGTGGAGAAAGGGAAGTCTAAAAAACTCAAAAGCGTCAA
GGAACTGCTGGGCATCACAATCATGGAGCGATCAAGCTTCGAAAAAAACCCCATCGACTTTCTCGAGGCGA
AAGGATATAAAGAGGTCAAAAAAGACCTCATCATTAAGCTTCCCAAGTACTCTCTCTTTGAGCTTGAAAACGG
CCGGAAACGAATGCTCGCTAGTGCGGGCGAGCTGCAGAAAGGTAACGAGCTGGCACTGCCCTCTAAATACG
TTAATTTCTTGTATCTGGCCAGCCACTATGAAAAGCTCAAAGGGTCTCCCGAAGATAATGAGCAGAAGCAGCT
GTTCGTGGAACAACACAAACACTACCTTGATGAGATCATCGAGCAAATAAGCGAATTCTCCAAAAGAGTGATC
CTCGCCGACGCTAACCTCGATAAGGTGCTTTCTGCTTACAATAAGCACAGGGATAAGCCCATCAGGGAGCAG
GCAGAAAACATTATCCACTTGTTTACTCTGACCAACTTGGGCGCGCCTGCAGCCTTCAAGTACTTCGACACC
ACCATAGACAGAAAGCGGTACACCTCTACAAAGGAGGTCCTGGACGCCACACTGATTCATCAGTCAATTACG
GGGCTCTATGAAACAAGAATCGACCTCTCTCAGCTCGGTGGAGACAGCAGGGCTGACCCCAAGAAGAAGA
GGAAGGTGACCGGTGGCAGCGGAGCTACTAACTTCAGCCTGCTGAAGCAGGCTGGAGACGTGGAGGAGA
ACCCTGGACCTGCCGGTGCCGGTATGGTGAGCAAGGGCGAGGAGGATAACATGGCCATCATCAAGGAGTT
CATGCGCTTCAAGGTGCACATGGAGGGCTCCGTGAACGGCCACGAGTTCGAGATCGAGGGCGAGGGCGA
GGGCCGCCCCTACGAGGGCACCCAGACCGCCAAGCTGAAGGTGACCAAGGGTGGCCCCCTGCCCTTCGC
CTGGGACATCCTGTCCCCTCAGTTCATGTACGGCTCCAAGGCCTACGTGAAGCACCCCGCCGACATCCCCG
ACTACTTGAAGCTGTCCTTCCCCGAGGGCTTCAAGTGGGAGCGCGTGATGAACTTCGAGGACGGCGGCGT
GGTGACCGTGACCCAGGACTCCTCCCTGCAGGACGGCGAGTTCATCTACAAGGTGAAGCTGCGCGGCACC
AACTTCCCCTCCGACGGCCCCGTAATGCAGAAGAAGACCATGGGCTGGGAGGCCTCCTCCGAGCGGATGT

ACCCCGAGGACGGCGCCCTGAAGGGCGAGATCAAGCAGAGGCTGAAGCTGAAGGACGGCGGCCACTACG
ACGCTGAGGTCAAGACCACCTACAAGGCCAAGAAGCCCGTGCAGCTGCCCGGCGCCTACAACGTCAACAT
CAAGTTGGACATCACCTCCCACAACGAGGACTACACCATCGTGGAACAGTACGAACGCGCCGAGGGCCGC
CACTCCACCGGCGGCATGGACGAGCTGTACAAGTAA
